# Gut microbiota analysis of Blenniidae fishes including an algae-eating fish and clear boundary formation among isolated *Vibrio* strains

**DOI:** 10.1038/s41598-022-08511-7

**Published:** 2022-03-17

**Authors:** Masa-aki Yoshida, Takuma Tanabe, Hideo Akiyoshi, Makoto Kawamukai

**Affiliations:** 1 Oki Marine Biological Station, Faculty of Life and Environmental Sciences, Shimane University, Oki, Shimane Japan; 2 Faculty of Life and Environmental Sciences, Shimane University, Matsue, Shimane Japan

**Keywords:** Microbiome, Microbiology, Comparative genomics

## Abstract

Some marine fishes are algae-feeding, and the microorganisms in their digestive tracts produce carbohydrate hydrolyzing enzymes such as agarose and fucosidase, which are potentially interesting resource for new functional enzymes. The purpose of this study was to establish a method for identifying and utilizing characteristic bacteria from the intestines of two algae-eating fish species: *Andamia tetradactylus*, which exclusively eats algae on the rock surface, and stellar rockskipper *Entomacrodus stellifer*, which feeds on both algae and invertebrates. We tested the species composition of the intestinal bacterial flora and found that Proteobacteria were commonly found both in species as in the common gut communities of marine fish, whereas Spirochaetes and Tenericutes occupied the flora of *A. tetradactylus*. We then performed anaerobic and aerobic cultures and isolated 34 and 44 strains including 48 strains belonged to *Vibrio* species from *A. tetradactylus* and *E. stellifer*. We observed that some *Vibrio* strains formed a clear boundary to avoid contacting other strains of bacteria. Whole-genome sequencing of such two *Vibrio alginolyticus* strains revealed two cyclic chromosomes commonly found in the genome of *Vibrio* species, and some unique genes encoding alginate lyase, chitinases, and type I-F CRISPR-associated endoribonuclease for the first time in *Vibrio alginolyticus*.

## Introduction

Gut flora generally includes bacteria, fungi, viruses (bacteriophages), protozoa, and parasites. Microbes that influence their host organisms are particularly important. The intestinal microbiome has been a subject of an emerging interest, since accumulating evidence indicates that the gut flora of humans significantly affects their health. Gut bacteria provide benefits for host cells by supplying essential nutrients such as vitamin K^1,2^. Some bacteria may provide a calorific boost to the host by breaking down ingested plant carbohydrates such as porphyrin, a sulfated carbohydrate derived from red algae that human enzymes cannot utilize^3^. *Bacteroides plebeius,* which breaks down agarose, is predominantly found in human populations that favor algae as a regular diet^4^. The composition of the gut microbiota is governed by a combination of environmental factors, (including diet and drugs) and host genetics.

Although many studies have been focused on human intestinal bacteria, little has been studied in intestinal bacteria of fish. Studies of culturable species do not reflect their actual composition, and non-culturable species are important to consider as a whole. Direct amplification of microbial DNA from gut extracts is a useful method for identifying gut flora. In previous studies, fish gut samples showed that Proteobacteria and Firmicutes were the most dominant phyla^5–7^. Meta-analysis showed that gut bacterial communities of 25 individual fish species exist depending on trophic level (herbivores, carnivores, or omnivores), habitats (saltwater, freshwater, estuarine, or migratory), and sampling methods^6^. For example, the effect of osmotic pressure on gut microbiota in the environment has been investigated. *Oryzias* is a broad-salinity fish genus that has been used as a model organism for progressive hypotonic transfer experiments^8^. The gut of *Oryzias* fish comprised of *Vibrio* at the genus level, but this was replaced by *Pseudomonas* after fish were transferred to the freshwater. The understanding of intestinal bacteria in these fish species has been gradually progressing, and is currently being applied to efficient aquaculture.

The digestive system plays an essential role in ingesting, digesting, and absorbing nutrients from food. The digestive systems of teleost fishes, composed of a digestive tract and accessory glands, are generally similar to those of other vertebrates^9–11^. The digestive tract of most teleosts is composed of the esophagus, stomach, pyloric caeca, and intestine^12–14^. In teleosts, the stomach is usually well developed, although it is absent in some forms. Stomachless fishes have no gastric glands or pyloric caeca. Unexpectedly, among the representative fish species whose genomes have already been sequenced, four are stomachless (tiger pufferfish, green pufferfish, medaka, and zebrafish) as opposed to gastric species (itoyo, tilapia, and cod). Zebrafish rapidly has become a well-recognized animal model for studying host-microbe-immune interactions because germ-free (GF) individuals can be reared to study host-microbe interactions^15^. 16S rRNA amplicon analysis showed abundant bacterial groups represented by 21 operational taxonomic units (OTUs) in normal zebrafish, dominated by members of the Proteobacteria (genera *Aeromonas* and *Shewanella*), followed by Fusobacteria, Firmicutes, Actinobacteria and Bacteroidetes^5^. Several generations of GF zebrafish larvae were found to be mono-associated with *Aeromonas veronii*^16^. In addition, reciprocal gut microbiota transplants between zebrafish and mice have been archived as GF recipients^17^. After transfer of the mouse microbiota into GF zebrafish, the relative abundance of Proteobacteria increased toward the microbiota composition of zebrafish. It appears that the host gut environment shapes the microbiota.

**Figure 1:**
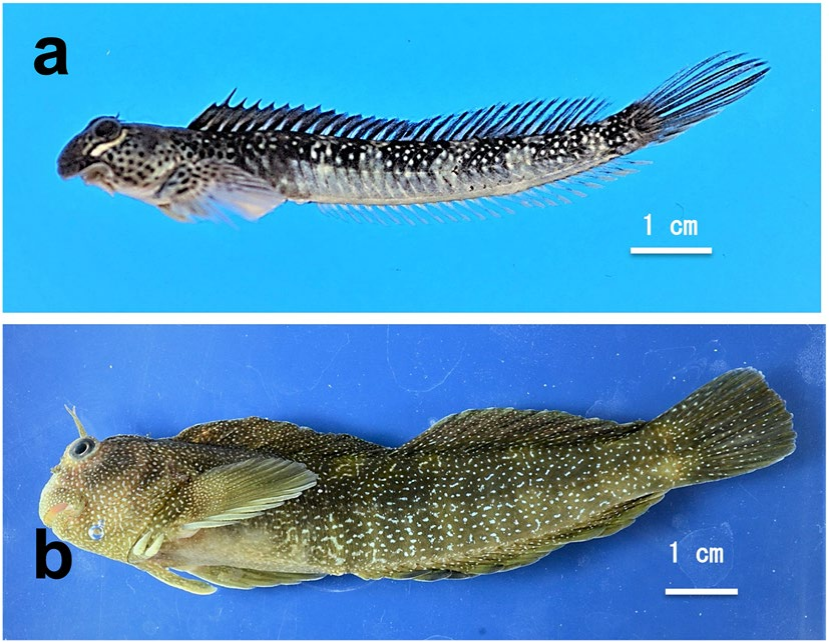
(**a**) *Andamia tetradactylus,* 82 mm. **(b**) *Entomacrodus stellifer,* 108 mm.

**Figure 2:**
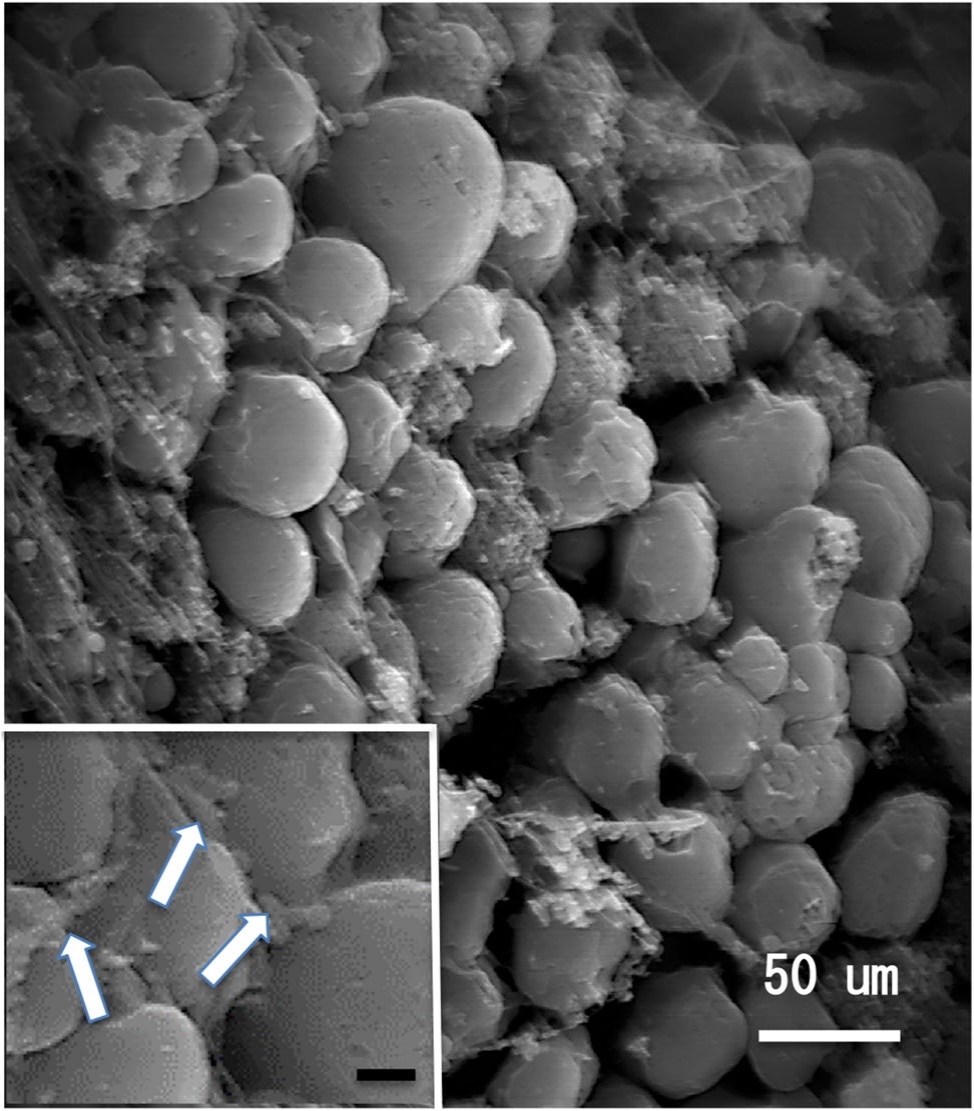
Scanning electron microscopic image of diatoms in the intestine of *Andamia tetradactylus*. Inset: The club-shaped bacteria (arrows) were seen on the surfaces of diatoms. Bar = 20 um.

In the marine environment, a variety of bacteria utilize enzymes to degrade such polysaccharides. Bacteria species, such as *Bacillus*, *Vibrio, Pseudomonas*, and *Microbulbifer* are sources of alginate lyases^18^ and the industrially essential enzymes used in food, biofuel, and biomedical industries^19^. Seaweed is also an effective source of various high-molecular polysaccharides, such as fucoidan and is a useful resource for probiotic products. Degraded microorganisms may be attached to consumed food as they enter the intestine. The long intestine is a special digestive tract that enables a symbiotic environment with these microorganisms, and is an incubator for microorganisms that decompose food, providing sufficient time for decomposition. Blenniidae is unique fish that feed on algae and invertebrates. The Stellar rockskipper, *Entomacrodus stellifer*, feeds on both algae and invertebrates in seawater. They were observed preying on small crustaceans during aquarium keeping. On the other hand, the rockskipper, *Andamia tetradactylus*, is characteristic as to exclusively eats algae on the rock surface (Fig. 1a). The rockskipper is found in the intertidal to supratidal zones of rocky shore washed by waves. They feed exclusively on algae where there is little competition from other fishes. The gastrointestinal tract also specializes in herbivory. The digestive system of Blenniidae fish, such as rockskippers, lack a stomach and are composed of almost spiral and longer intestines. Electron microscopic observation of the gut of the rockskipper showed that a large number of rod and short rod bacteria, which might contribute to the degradation of algae, were present on the surface of the gut contents (Fig. 2). Since the gut flora of the herbivorous fish have not been studied*,* we aimed to classify and identify the microorganisms in the intestines of the herbivorous fish species *A. tetradactylus* and *E. stellifer*. Using these two species as a model case, the first step was to identify the characteristic bacteria in their intestines and establish a culture method for them. The composition of the intestinal bacterial flora from the two herbivorous fish species was examined by 16S rDNA amplicon sequencing. We then cultivated bacteria from the two fish species on agar plates, and unexpectedly, found that *Vibiro alginolyticus* strains formed a clear line between species on the agar plate. Finally, whole-genome sequencing of two *V. alginolyticus* strains was conducted, and genes coding alginate lyases were found.

## Results

### Intestinal flora of herbivorous fish

**Figure 3:**
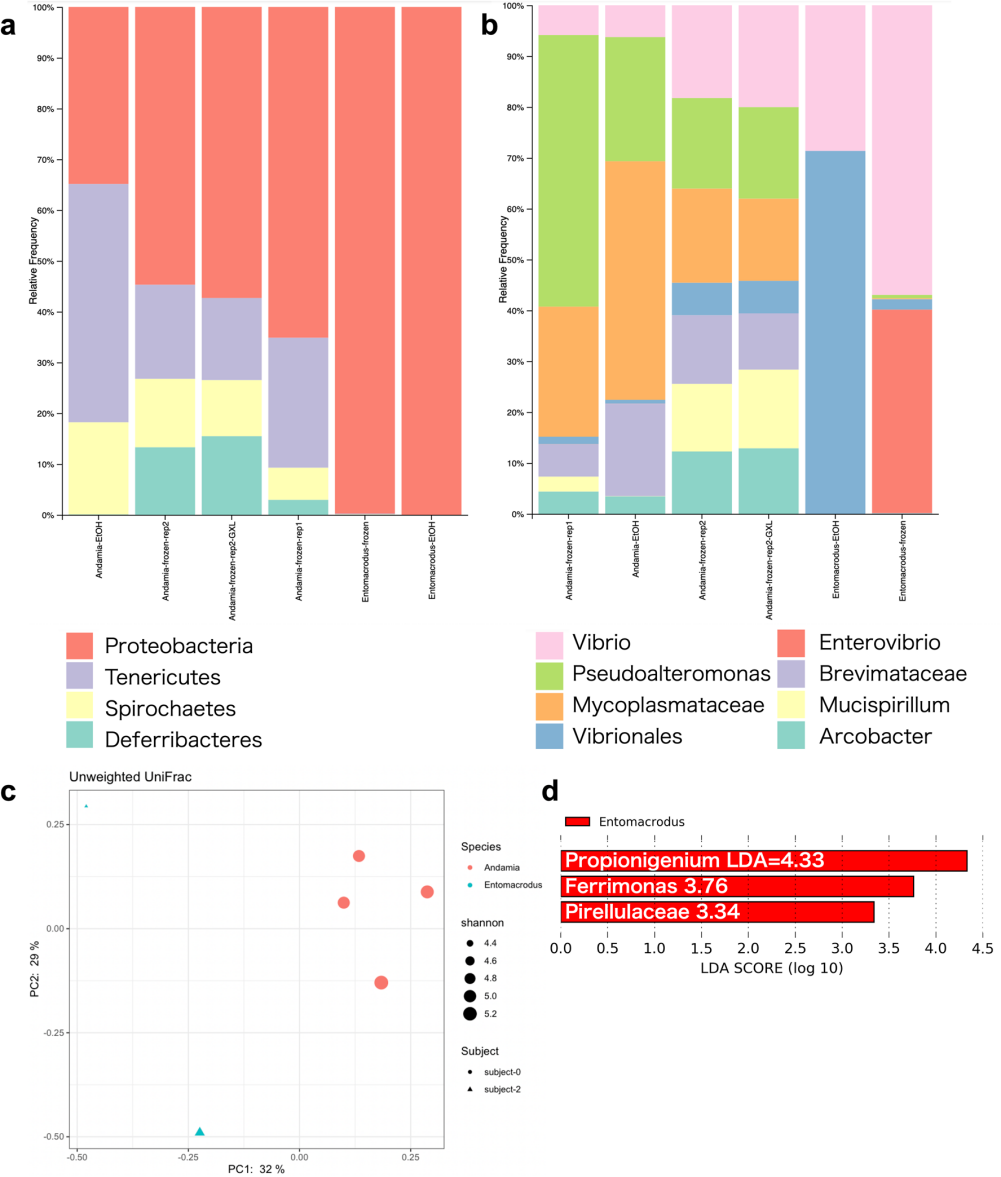
Major groups of bacteria detected by 16S rDNA amplification from *Andamia tetradactylus* and *Entomacrodus stellifer*. **(a)** Relative abundance of phylum (class 2) category was shown in individual gut microbiome samples. Categories with less than 10,000 reads are not shown to represent a major proportion of the bacterial population. **(b)** Relative abundance of genus (class 6) category was shown in individual gut microbiome samples. For the three groups, Mycoplasmataceae, Vibrionales, and Brevimataceae, their family or order names are given since they have not yet been identified as genera. **(c)** Principal Coordinate Analysis (PCoA) plots using distance matrices for beta-diversity (unweighted UniFrac distance). **(d)** A forest plot showing differentially abundant bacterial taxa that were significantly different between species groups as determined using the Kruskal–Wallis test. LDA score (effect size) indicating significant differences in bacterial taxa (LDA score > 3.0; alpha value *p* < 0.05).

High-throughput sequencing yielded 89,615–128,023-reads for each sample. The QIIME 2-based microbial community analysis yielded 749 representative OTUs. Based on phylogenetic classification using the Greengenes 13_8 16S rRNA gene database, most reads were classified as Proteobacteria, Spirochaetes, and Tenericutes  (class 2, Fig. 3). A rarefaction analysis was carried out at species level. When numbers of observed OTUs plotted against numbers of reads, all samples except for the ethanol-fixed sample of *E. stellifer* reached a plateau, and the difference between samples was small (Fig. S1). This indicates that the sequencing depth was sufficient to carry out a thorough description of each sample. To statistically analyze the diversity of the herbivorous gut microbial communities, we examined alpha and beta diversity metrics implemented in QIIME 2. Community dissimilarity between individuals was statistically examined between the following two categories: species (*A. tetradactylum* or *E. stellifer*) and fixation method (ethanol or frozen). According to the diversity analysis of QIIME2, there was no significant difference in diversity between species or fixation (Fig. S2). While the number of observed OTUs was higher in the ethanol-fixed sample (p = 0.046, Kruskal–Wallis test; Fig. S2b), Shannon diversity for *A. tetradactylum* and *E. stellifer* was not significantly different (p = 0.83, Fig. S2d). Thus, *A. tetradactylum* and *E. stellifer* have gut microbiomes that contain similar numbers of OTUs.

In the *A. tetradactylum* microbial community, Proteobacteria, Spirochaetes, and Tenericutes (class 2) were dominant, accounting for more than 80% of the bacterial diversity in all samples. In contrast, *E. stellifer* was particularly rich in Proteobacteria (Fig. 3a). Among Proteobacteria, Vibrionales was found to be the most abundant in both hosts, Vibrionales alone accounted for more than 20% of the total bacterial flora in both hosts, regardless of the presence or absence of fixation (Fig. S3). Among the Proteobacteria, Rhodbacterales was the second most abundant (Fig. S4). Campylobacterales were also abundant, but they were characterized by a high variability among the samples.

We attempted to determine how similar are the samples obtained from the different sites in terms of their identified microbial communities. The fecal microbiota for the *A. tetradactylum* and *E. stellifer* groups were clearly separated by Principal Coordinate Analysis (PCoA) plots of beta diversity of unweighted UniFrac distance (Fig. 3b). *Andamia* samples were generally similar regardless of the individual or experimental technique, whereas *Entomacrodus* showed a different microflora profile. We also used linear discriminant analysis (LDA) of effect size (LEfSe) to determine the taxa that most likely explained the differences between the *A. tetradactylum* and *E. stellifer*. When performing the LEfSe analysis, we found that the two genera *Propionigenium* and *Ferrimonas* were significantly enriched in the samples of *E. stellifer* (Fig. 3d, LDA scores = 4.33 and 3.76, respectively, and *p* < 0.05). The family Pirellulaceae and associated unknown genus also showed enrichment with a high LDA score in the *E. stellifer* samples (Fig. 3d, LDA score = 3.34 and *p* < 0.05)). Although undetectable in LEfSe analysis, we note that the following two families showed significant variations in beta-diversity metrics: Brachyspiraceae, and Spirochaetaceae (class 5, P < 0.05, ANCOM results). The two families of Spirochaetes were found only in *A. tetradactylum* (Figs. S5, S6), consistent with the prominence of Spirochaetes in *A. tetradactylum*. The high frequency of Tenericutes in *A. tetradactylum* was due to the abundance of Mycoplasma species (Fig. S7); however, this difference was not statistically significant.

To evaluate differences in community functional attributes, we used PICRUSt2. We found 118 EC gene families with statistically significant differences between the two groups of species (FDR *q* < 0.05) (Fig. S8). EC:2.7.7.71 (biosynthesis of GDP-D-glycero-alpha-D-manno-heptose) was most significantly increased in the *Entomacrodes* samples. We also found 132 COG with statistically significant differences between the two groups of species (FDR *q* < 0.05) (Fig. S9). COG4637 (Predicted ATPase) was most significantly increased in the *Entomacrodes* samples.

### Isolation of bacteria by culturing on agar plates

**Figure 4:**
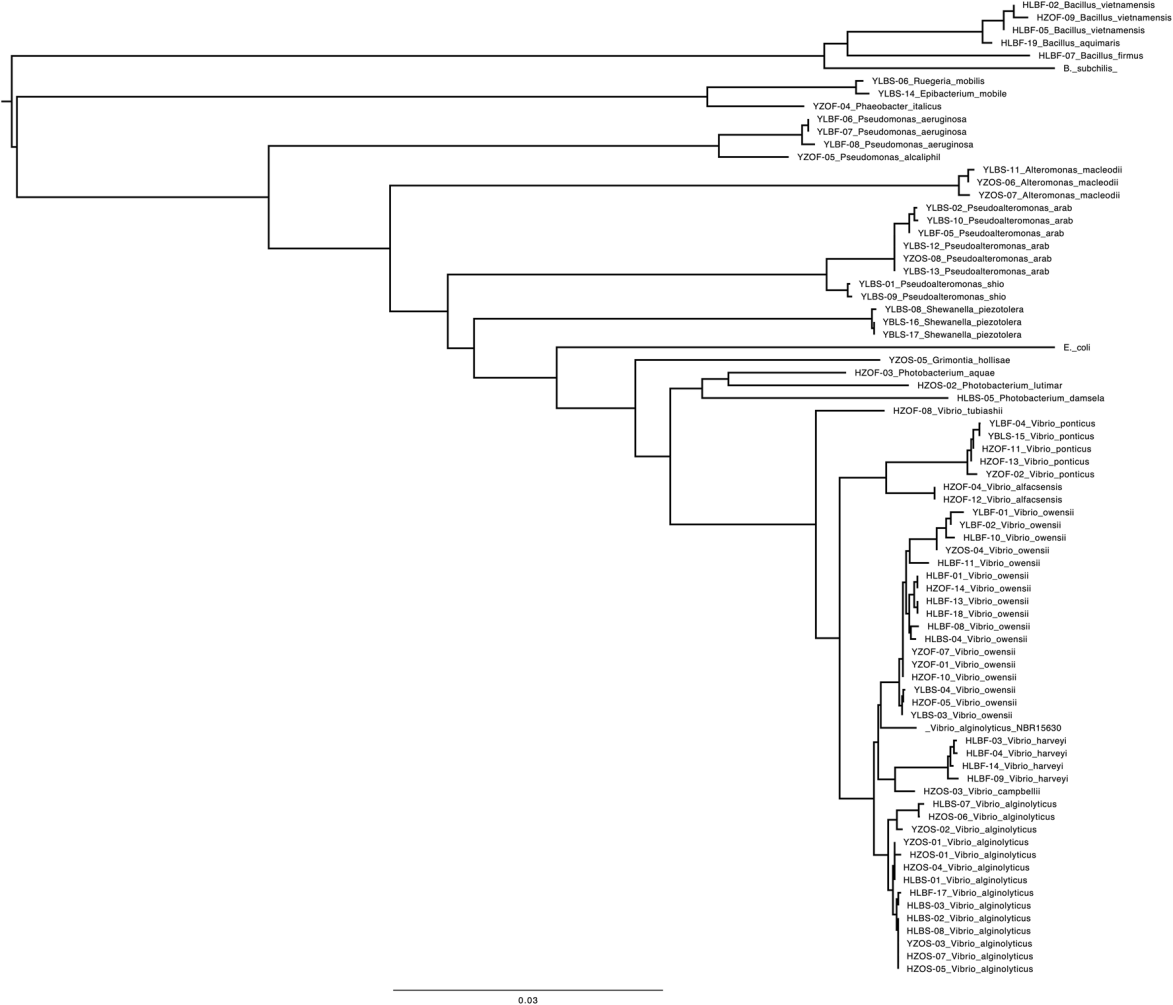
Phylogeny of isolated bacteria from *Andamia tetradactylus* and *Entomacrodus stellifer*. The 35 and 40 strains were isolated from *A. tetradactylus* and *E. stellifer,* respectively.16S rDNA of those strains were amplified by PCR and determined by sequencing. Based on those sequences, the phylogenetic tree was constructed by the ClustalW program (DDBJ, Japan).

We cultured bacteria from the intestines of *A. tetradactylus* and *E. stellifer* on Zobell and LB plates. Bacterial colonies were picked and isolated as single colonies. Single colonies were then examined for 16S rDNA after amplification of the 16S rDNA region by PCR and sequencing. A total of 35 and 40 strains were isolated from *A. tetradactylus* (Table S1) and *E. stellifer* (Table S2), respectively. Some strains were indistinguishable from other strains only by 16S rDNA sequences and indicated, as in Tables S1 and S2. The phylogenetic tree of the isolated bacteria is shown in Fig. 4. Most of the *A. tetradactylus* guts were concentrated in four genera: *Vibrio* spp., *Pseudoalteromonas* spp., *Pseudomonas* spp., and *Shewanella* spp., whereas *E. stellifer* isolates were concentrated in three genera: *Vibrio* spp., *Photobacterium* spp., and *Bacillus* spp. There were also single species isolates, such as *Alteromonas macleodii, Paeobacter italicus, Ruegeria mobillis, Epibacterium mobile,* and *Grimontia* sp. from *A. tetradactylus*. The number of isolated species were larger in *A. tetradactylus* than in *E. stellifer,* although the numbers of isolates were opposite. A wide range of *Vibrio* spp. including *V. alginolyticus, V. harvei, V. owensii, V. alfacsensis,* and *V. ponticus,* were found in the guts of both fish species. *Vibrio* spp. have been frequently identified in other marine organisms. We previously isolated an agar-degrading bacterium from the gastrointestinal tracts of *A. tetradactylus*, but to the date, no agar-degrading bacteria have been isolated from the intestine.

**Figure 5:**
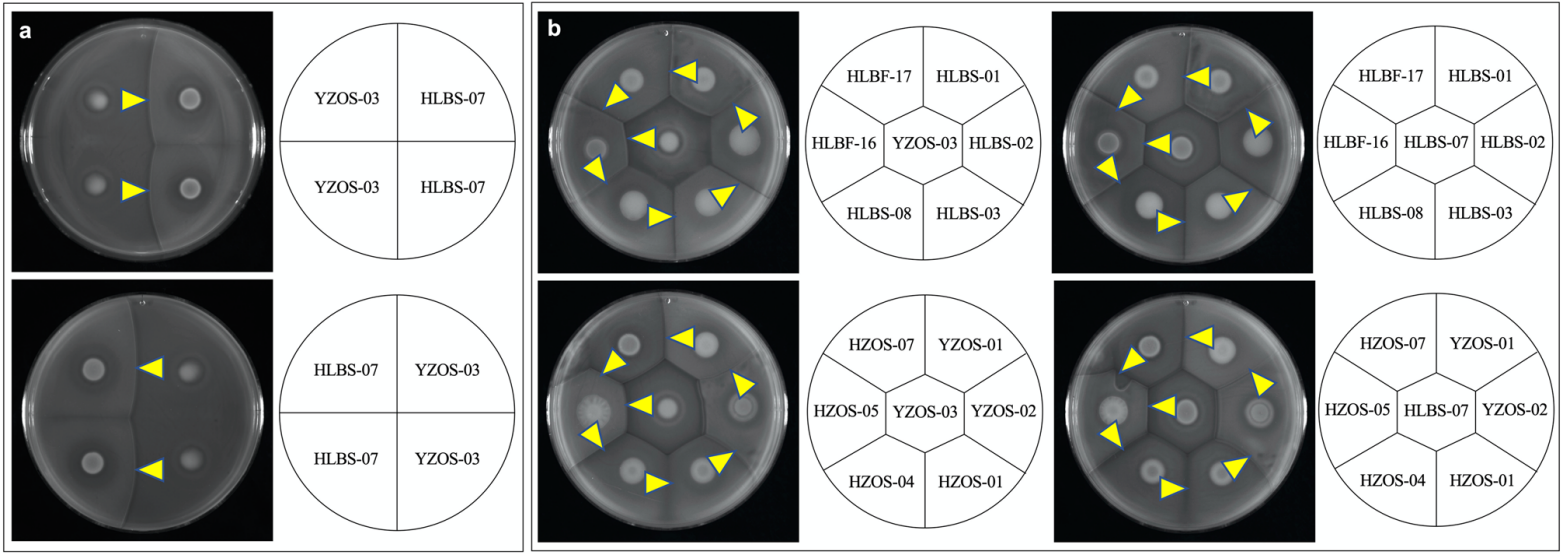
The formation of territory among the bacteria isolated from *Andamia tetradactylus* and *Entomacrodus stellifer*. (**a**) Two individual colonies of HLBS-07 and YZOS-03 strains belonging to *V. alginolyticus* were grown on Zobell medium at 30 °C for 2 days. (**b**) The indicated strains belonging to *V. alginolyticus* were grown on Zobell medium at 30 °C for 2 days. Arrowheads point to the boundary between strains.

Among the postulated *Vibrio* spp. isolated in this study, 14 strains are migratory. The isolated *Vibrio* spp. were spotted on the agar plate and they started moving on the agar plate by flagella until they met with other strains (Fig. 5a). We found that these *Vibrio* strains exhibited characteristic phenotypes of their boundary formation. HLBS-07 and YZOS-03 formed a very clear boundary line (Fig. 5a). They did not invade the territories of the other strains not like HLBS-07 itself or YZOS-3 itself (Fig. 5a). We then tested the boundary formation of YZOS-03 and HLBS-07 with HLBS-01, HLBS-02, HLBS-03, HLBS-08, HLBF-16, HLBF-17, YZOS-01, YZOS-02, HZOS-04, HZOS-05 and HZOS-07 strains, which all belong to *Vibrio* spp. All strains formed boundary lines with HLBS-07 and YZOS-03 on the Zobell medium (Fig. 5b). These boundary lines were less clear between HLBS-01 and HLBS-02 strains, or between YZOS-01, and YZOS-02 strains. Clear territory formation occurred only between different isolates and not within the same *Vibrio* isolates.

### Complete genome sequencing of *Vibrio* strains cloned from the herbivorous fish

For YZOS-03, 1,427,401 nanopore reads (11,170,207,911 bp) were obtained with an N50 length of 13.9 kbp. For HLBS-07, 1,150,246 reads (9,836,928,300 bp) were obtained with an N50 length of 15.0 kbp. The subsampled reads of YZOS-03 were assembled with Canu v2.0 into three circular contigs (3,351,300, 1,901,164 and 95,862 bp). We discarded the shorter contig because this contig was 100% identical to one part of the largest contig. Therefore, we selected Canu assembly for subsequent steps. Consensus assembly was called using the MarginPolish with 1,427,401 reads, followed by the Medaka polishing. Racon polishing worsened the BUSCO score and was not used (Table 1). We compared the BUSCO score with that of the Flye assembly and selected the Canu + MarginPolish + Medaka polished assembly for the subsequent analysis (Table 1). After the polishing process, the total nucleotide genome of YZOS-03 was 5,145,821 bp, with a G + C content of 44.7% and BUSCO score of 98.9%. The subsampled reads of HLBS-07 were also assembled with Canu v2.0 into two contigs (3,794,970 and 11,408 bp). The longer contig was circularized by searching overlapping fragments from the raw reads, although the shorter contigs was circular. After the polishing process, the total nucleotide genome of HLBS-07 was 5,338,402 bp with a G + C content of 44.5%.

**Table 1:** Comparison of BUSCO completeness of genome assemblies.

Assembler + Polishers	YZOS-03					HLBS-07		
	Canu	Flye	Canu + Racon	Canu + MarginPolish	Canu + MarginPolish + Medaka	Canu	Canu + MarginPolish	Canu + MarginPolish + Medaka
# contigs	3	2	3	3	3	2	2	2
Total length	5,195,096	5,145,162	5,195,786	5,196,640	5,196,188	5,336,637	5,338,931	5,338,402
BUSCO score*	94.7	97.4	87.6	95.5	98.9	91.5	95.3	98.5
# Complete	1368	1407	1265	1379	1429	1322	1377	1423
#Fragmented	49	21	107	43	9	83	43	13
#Missing	28	17	73	23	7	40	25	9

*Results from dataset vibrionales_odb.

**Figure 6:**
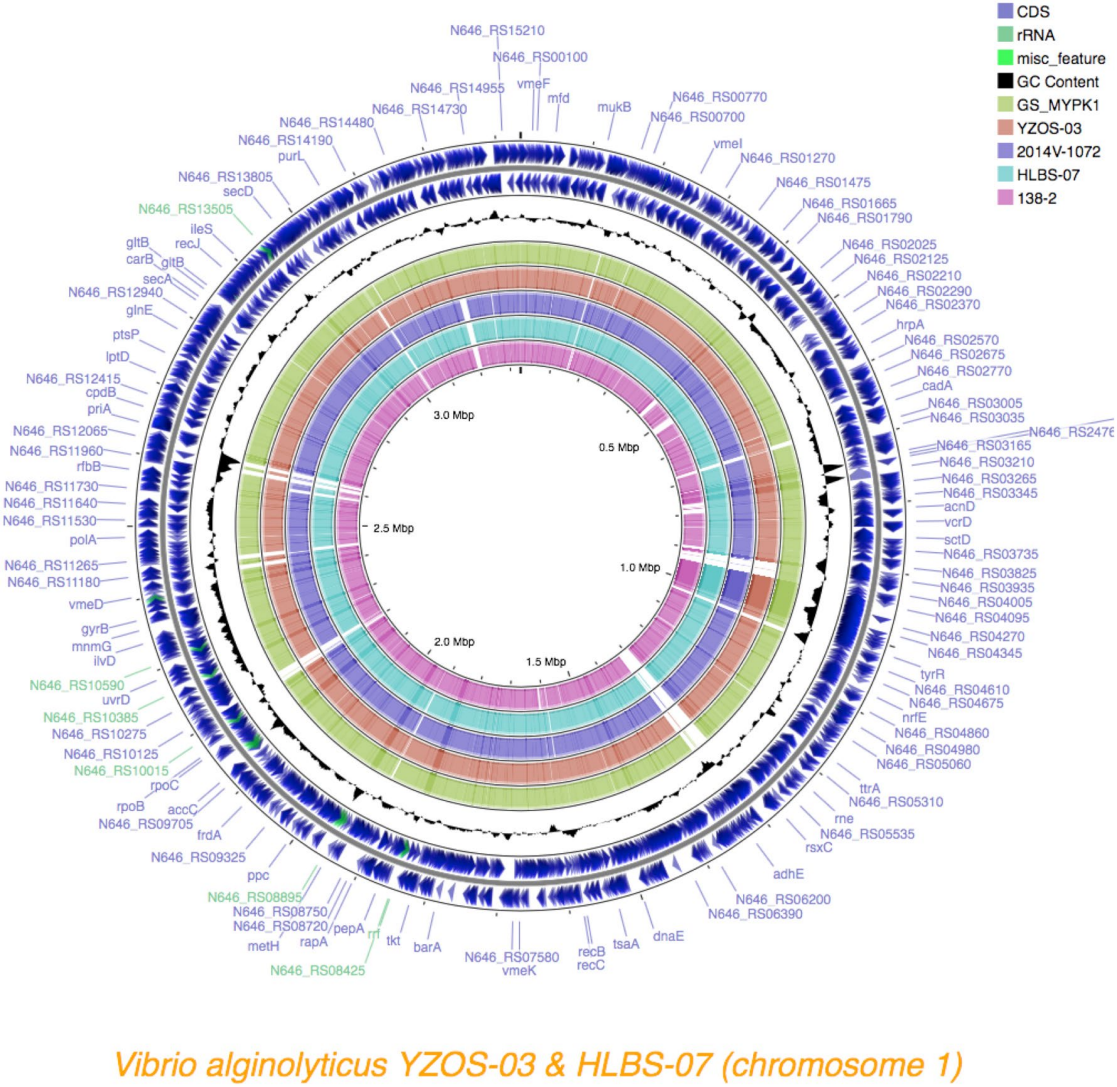
Structure of chromosome1 of *Vibrio alginolyticus*. Chromosomal sequences of *V. alginolyticus* G3_MYPK1, 2014V-1072,138-2 HLBS-07 and YZOS-03 strains were mapped based on NBRC15630. CDSs were estimated with Prokka analysis on the CGview.

**Figure 7:**
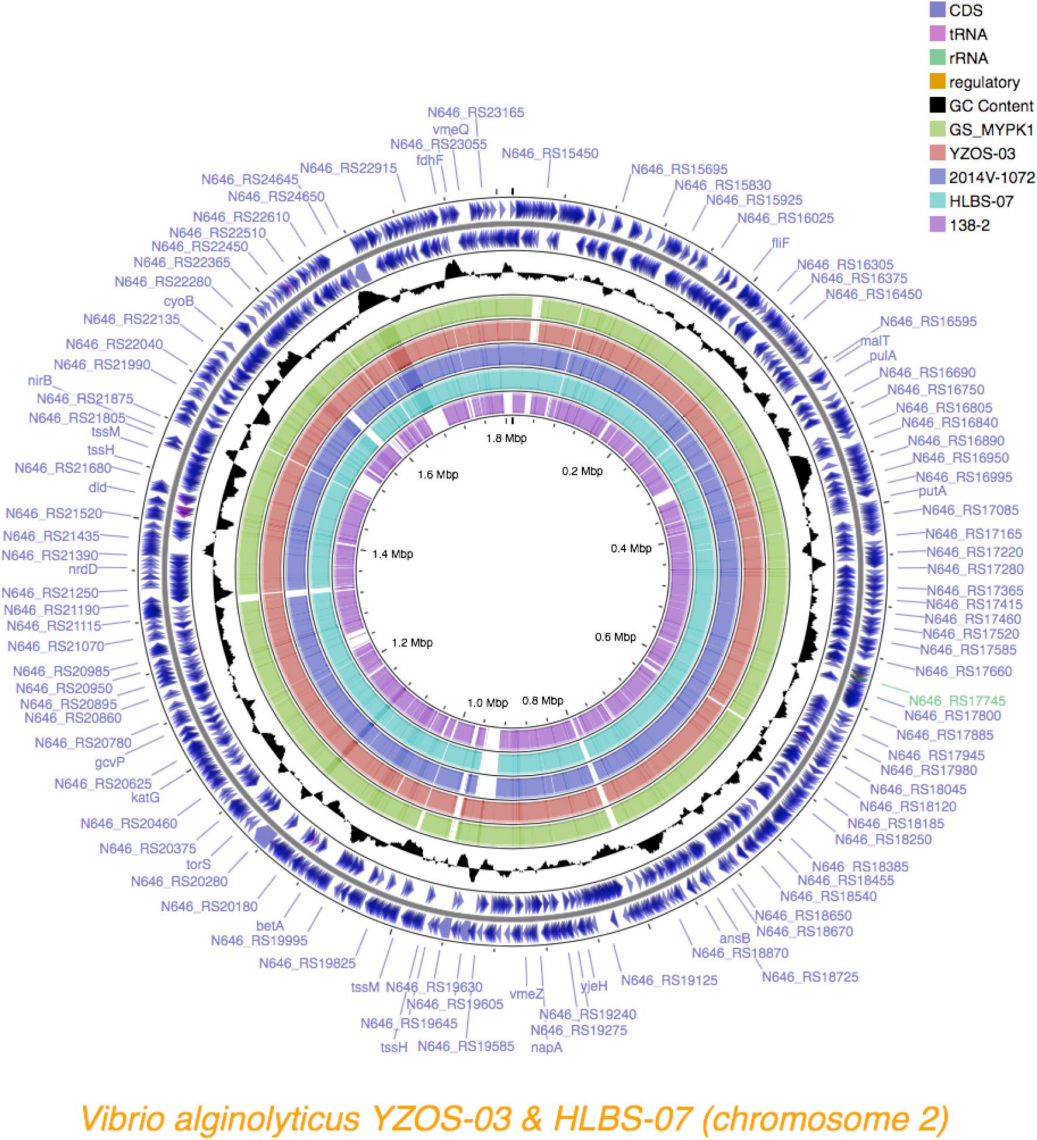
Structure of chromosome2 of *Vibrio alginolyticus*. Chromosomal sequences of *V. alginolyticus* G3_MYPK1, 2014V-1072,138-2 HLBS-07 and YZOS-03 strains were mapped based on NBRC15630. CDSs were estimated with Prokka on the CGview.

DFAST identified 4631 predicted protein-coding sequences, 37 rRNA genes, and 129 tRNA genes for YZOS-03. In addition, 4856 predicted protein-coding sequences, 40 rRNA genes, and 130 tRNA genes were identified for HLBS-07. Circular visualization of the genomes was carried out on the CGview server v1.0 (Figs. 6, 7). Comparison of genomic sequences of YZOS-03 and HLBS-07 with the type strain of *V. alginolyticus* NBRC 15630 (= ATCC 17749, accession numbers NC_022349.1 and NC_022359.1) revealed that HLBS-07 had a more similar overall nucleotide identity (99.14% for Chr1, 98.23% for Chr2) than YZOS-03 (98.66% for Chr1, 98.46% for Chr2). The closest strain to HLBS-07 based on chromosome identity was 2014 V-1072 (99.62% for Chr1, 98.88% for Chr2, Fig. 6) and that of YZOS-03 was G3_MYPK1 (99.08% for Chr1, 98.62% for Chr2, Fig. 7).

Next, we analyzed genes that were common to YZOS-03 and HLBS-07, but not in the genome of the type strain *V. alginolyticu*s NBRC15630 (GCF_000354175.2). Among the genes in HLBS-07, 234 genes (e-value threshold 1e−10) were not detected in BLASTP in either the YZOS-03 or NBRC15630 strains. Similarly, 181 genes were unique to YZOS-03. We found that both strains encoded two alginate lyases, which hydrolyze the major polysaccharides in seaweed^18^. Both strains encoded five different chitinases, which are also widely found in many other species^20,21^. Complete sets of genes encoding menaquinone- and ubiquinone-synthesizing enzymes are present in both strains^22^. We found both YZOS-03 and NBRC15630 had gene encoding a cellulose synthase operon (*BcsB, A, E*) and endoglucanase (*BcsZ*), but HLBS-07 did not. Orthologues of *fepB, fepG, entS, fes*, and *murB* were missing in both the HLBS-07 and YZOS-03 strains, but were present in NBRC15630. Ten *tnpA* (8 on ChrI and 2 on ChrII) encoding transposases were missing in YZOS-03, but all were present in HLBS-07 and NBRC15630. The *ulaA* and *fruA* genes encoding PTS enzymes, the *fatA, B, C, and D* genes encoding siderophore ABC transporters, as well as *gspA* were missing in YZOS-03 but present in HLBS-07. The *wecA*, and *ampC* genes were missing in HLBS-07, but were present in YZOS-03. There were many hypothetical proteins that are differently present among YZOS-03, HLBS-07 and NBRC15630 strains.

## Discussion

In this study, we surveyed the microbiota of the intestine of two fish species, *A*. *tetradactylus* and *E*. *stellifer*. *A. tetradactylus* is a stomachless and herbivorous fish, whereas *E. stellifer* is stomachless and omnivorous. We expected that studying microbiota in these two fish species and comparison with that in other species would reflect the differences in their food habits. Indeed, our analysis of 16S rDNA amplicon sequencing revealed different flora in the two fish species. Bacteria belonging to Proteobacteria were commonly found in both fish species, whereas those belonging to Spirochaetes and Tenericutes were predominant in *A. tetradactylus* but not in *E. stellifer.* The results of the QIIME2 analysis showed that Mycoplasma is the preferred bacterium in the Tenericutes phylum in *Andamia*, although this was not statistically significant. Mycoplasma are small and predominantly anaerobic, and is thought to reside deep in the intestinal tract. The differences with *E. stellifer* may be due to the effect of suspension of the intestinal tract during extraction. Fusobacteria were observed in the EtOH-fixed samples but not in frozen samples. This may stem from difference in the sampling methods. The presence or absence of ethanol-fixation and type of PCR enzymes were not significant. In particular, in the case of *E. stellifer*, the highest diversity was detected in ethanol-fixed samples, indicating that the similar detection is possible with ethanol-fixed fish intestinal contents.

Previous studies have indicated that Proteobacteria, in addition to Bacteroidetes and Firmicutes, comprise 90% of the fish intestinal microbiota^23^. Actinobacteria are also found at high frequencies^24^. The gut of *A. tetradactylus*, which is rich in Spirochaetes and Tenericutes, is very different from these previous findings. In marine fish, the genera *Vibrio*, *Photobacterium*, and *Clostridium* were often reported to be dominant. Proteobaceria are commonly found in herbivorous and carnivorous fish^6^. A meta-analysis of the gut communities of marine fish revealed that Vibrionales bacteria, which include the genera *Vibrio* and *Photobacterium*, accounted for 70% of sequence reads^6^. It is reasonable to consider that freshwater fish do not harbor *Vibrio* spp. in their guts, because *Vibrio* are generally found in seawater. In the present study, Firmicutes containing *Clostridium* were only found in very small numbers in half of the six samples, and were rarely found in the algae-eating fish in this study. The other genera identified included *Aeromonas*, *Photobacterium*, *Pseudomonas*, which have all been previously identified in fish gut microbiota that might aid digestion^25^. *Aeromonas* and *Pseudomonas* are notable in the fish gut as being both pathogenic and probiotic bacteria, but were only found in very small numbers in the two species in our study. In summary, the absence of Firmicutes and Actinobacteria was what distinguished the algae-eating fish from other species. However, it is known that *Clostridia* also dominate the gut microbial flora in different marine herbivorous fish species^26^. Among all fish species tested, marine herbivorous fishes have been shown to have a high diversity of gut bacteria^6^. This is in good agreement with the diversity of gut bacteria in the absolute algae-eating *Andamia*, where even the most abundant species of the bacterial phyla are as low as 30%. Marine herbivorous fishes also harbor few of the typical environmental bacteria, yet many close relatives of bacteria from mammalian guts^6^. The presence of short-chain fatty acids in the gut of marine herbivores suggests that herbivorous fish and mammals are similar in the process of enteric fermentation^27^.

Le and Wang^28^ found that Brevinemataceae (phylum Spirochaetes) and Mycoplasmataceae (phylum Tenericutes) are the most abundant species in the gut microbiota of the mullet. Diverse Spirochaetes are regularly found deep in marine sediments and soils, and in the digestive tracts of arthropods and several species of mammals. Spirochaetes are commonly detected at low concentrations in the gut microbiota of fish; however, Givens et al.^29^ reported that one of the three barracuda individuals contained a community comprised of 99% Spirochaetes and the three *Mahi mahi* (common dolphinfish) individuals contained 64–98% Spirochaetes. This feature has also been reported in old gilthead snappers^30^ and puffer fish^31^. The common diet of these fish species is difficult to clarify, but may be related to the predation of the whole sediment.

The composition of Fusobacteria and Proteobacteria in *E. stellifer* is similar to that found in other marine fish, such as *Silurus* and *Carassium*^24^, and to results reported for freshwater garpike^32^. A few studies have shown Fusobacteria to be the dominant members of the gut microbiota of freshwater fish^33,34^. Although *E. stellifer* is stomachless, this species is considered to be an omnivore, and the above tendency is similar to that of zooplankton-eating fish species. In addition, Fusobacteria are anaerobic gram-negative rods that produce butyric acid^35^, a short-chain fatty acid that is the end product of fermentation of carbohydrates, including those found in mucins^36,37^. This fatty acid has been found in the guts of herbivorous and omnivorous fish^27,38^. Nuez-Ortin et al.^39^ demonstrated the ability of butyric acid to inhibit potential pathogens in freshwater fish, and butyrate sodium is now marketed as a food additive to promote fish health and growth. This is likely to be the case for similar probiotics in marine fish, and further validation of their efficacy is expected.

Interestingly, the novel Mycoplasma phylotype was found to be predominant in wild Atlantic salmon in Scotland and in fish raised in pens, while *Acinetobacter junii* was predominant in farmed fish in Norway. The farmed fish in these two locations were fed different diets. In another study that examined changes in the gut microbiota of salmon over their life cycle, it was observed that Proteobacteria were predominant at all stages, with an enrichment of Tenericutes (especially *Mycoplasma* spp.^40^). Tenericutes predominant gut microbiota is also found in some fish species, but is difficult to culture and has not yet been studied.

In this study, many *Vibrio* spp. were isolated, in addition to some strains of *Shewanella*, *Photobacterium,* and *Bacillus*, which were obtained only in *E. stellifer*. In comparison with NGS, minor strains were isolated, but strains that matched the culture conditions may have been prioritized. Among the strains, we found a very clear boundary that resembled an artificial line drawn that did not merge among the isolated *Vibrio* spp. The coordinated behavior of swarming was known to enable *Vibrio* spp. to colonize surfaces, coordinate behavior, and form multicellular communities^41^. The peritrichous lateral flagella is essential for swarming and the expression of flagella forming genes are regulated by the quorum sensing regulators *aphA* and *opaR*^42^. We found *opaR* in the genomes of *V. alginolyticus* YZOS-03 and HLBS-07, annotated as *luxR*, and the sequence similarity between these strains is 100% among *Vibrio* spp. On the other hand, *aphA* is apparently absent both in the YZOS-03 and HLBS-07 strains. Swarming ability of *Vibrio* spp. including YZOS-03 and HLBS-07 strains appears to be necessary for boundary formation, but its mechanistic analysis in *Vibrio* spp. has not been conducted. The molecular mechanism of boundary formation as a self-recognition mechanism has been studied mostly in *Proteus mirabilis*. A six-gene operon, *idsA-idsF,* of *P. mirabilis* has been identified as important for self versus non-self discrimination by forming a boundary between two strains^43^. We did not find apparent orthologs of *idsA-F* genes in the genomes of YZOS-03 and HLBS-07. Curiously, we found type I-F CRISPR-associated endoribonuclease Cas6/Csy4 and CRISPR-associated protein Cys3 in HLBS-07, but not in YZOS-03 or any other *V. alginolyticus* strains. This is a unique feature of the HLBS-07 strain.

*V. alginolyticus* is a halophilic anaerobic gram-negative bacterium frequently found in marine environments, and some species cause epidemic vibriosis. Whole genome sequencing of *V. alginolyticus* has been previously reported^44–48^. The genome size of 71 reported *V. alginolyticus* strains varied between 6.17 and 3.94 Mb with predicted ORF between 5619 and 3908. Among the 71 published *V. alginolyticus* genomes, YZOS-03 (5.14 Mb) is relatively small and HLBS-07 (5.33 Mb) is a medium-sized strain. Among these *Vibrio* genomes, the YZOS-03 and HLBS-07 strains were close to G3_MYPK1 and 2014V-1072, respectively. YZOS-03 and HLBS-07 were 98.7% identical. Both strains encoded two alginate lyases, which are commonly found in many *Vibrio* spp.^18^. The genes encoding alginate lyases, amylases, chitinases, and mannosidase are present in both strains, suggesting that the bacteria assist in the digestion of polysaccharides.

In summary, we investigated the bacteria of two stomachless fish species, *A. tetradactylus* and *E. stellifer,* and found a diversity in their habitats based on the direct sequencing of 16S rDNA. The isolated bacteria had some specific features including boundary formation in the two species. Combined with genomic DNA analysis of representative two *Vibrio* spp., the current analysis is useful for understanding of such mechanisms and for future application in the biotechnology field.

## Materials and methods

### Sample collection and rearing

We collected twenty Rockskipper, *A. tetradactyla*, from the coast of the Iriomote Island, Okinawa Prefecture, twelve Stellar Rockskipper, *E. stellifer*, from in the coast of Shimane Peninsula, Shimane Pref., in Japan.

To control for the influence of seasonal differences and growth stages, only adult-stage specimens were used. Because they are wild animals, it was difficult to randomize the sex and age of the animals. All specimens were caught using hand nets between the months of June and November from 2007 to 2019. The Stellar Rockskipper were maintained in 60 cm glass tanks (60 L) with running water under optimal oxygenation conditions, and the temperature was maintained at 22.0 ± 1.0 °C throughout the 3-day experimental period. The study protocol was approved by the animal ethics committee of Shimane University, and was conducted in strict adherence with the guidelines for the care and use of research animals set out by the committee and in compliance with the ARRIVE guidelines v1.0 since our experiments have done prior to ARRIVE guidelines v2.0 published on 2020^49^. Our experiments have done are planned with the following points in mind. To avoid overexploitation of wild populations, we used a minimal experimental scale including preliminary experiments, 2–3 individuals each for morphological observations, gut microbe isolation and metagenomic analysis, and totally 10 individuals for both species. The main purpose of this study was to describe the gut bacteria, and no study was conducted that required a control group. The age of the fish was 1 year old adult fish, and egg-carrying individuals were not used in the experiment, so that the individuals used in the experiment were males. After ice-cold anesthesia, the animals were quickly decapitated and subjected to humane killing.

### Tissue preparation

Excised samples of digestive tracts fixed with 1.5% glutaraldehyde were rinsed, dehydrated, conductive-stained by 2.0% tannic acid and 1.0% osmium tetroxide, and freeze-dried with t-butyl alcohol. Samples were subsequently coated with platinum and observed under a digital scanning electron microscope (S-4800; Hitachi High-Technologies Corp.).

### Flora analysis

Whole flesh frozen bodies of *A*. *tetradactylus* and *E*. *stellifer* were utilized for feces collection. For the ethanol-fixed samples, frozen fish was directly immersed in the 70% ethanol/milliQ water. Before dissection, tweezers were sterilized with 70% ethanol and UV light irradiation for 10 min. Fish bodies were dissected under a binocular microscope. The feces were roughly homogenized using tweezers in the C1 buffer included in the DNA Powersoil kit (QIAGEN). For the small *Andamia* fish, the whole gut was resuspended in the Powerbeads tube and utilized for the following procedure. DNA extraction was performed according to the manufacturer’s instruction.

The V4 region of the bacterial 16S rRNA gene was amplified using a 2 ng of DNA template with primers 515 F (5′-GTGCCAGCMGCCGCGGTAA-3′) and 806R (5′-GGACTACHVGGGTWTCTAAT-3′). PCR reactions were performed in 20 μL with ExTaq HS (Takara Bio Inc.) or PrimeSTAR GXL DNA Polymerase (Takara Bio Inc.) at an annealing temperature of 50 °C for 20 cycles. The PCR products were purified with the FastGene PCR Extraction kit and sent to Fasmac Inc, Japan. The amplicon library was sequenced by Illumina MiSeq 2 × 300 bp paired-end platform according to the manufacturer’s instruction. The MiSeq fastq reads are available in the DNA Databank of Japan (DDBJ) Sequence Read Archive (DRA) under the accession number DRA011079.

Read data was analyzed using QIIME 2^50^. Paired-end sequences imported into QIIME 2 were quality-controlled and combined using DADA2 (–p-trunc-len-f 240 –p-trunc-len-r 200 –p-trim-left-f 19 –p-trim-left-r 20)^51^. The settings for quality control were based on the reads’ quality distribution along the length of the sequence. Alpha rarefaction analysis, OTU, alpha diversity (number of observed OTUs, Shannon diversity, Faith phylogenetic diversity), and beta diversity (Jaccard distance, Bray–Curtis distance, unweighted and weighted UniFrac distance) were analyzed using QIIME 2. To evaluate the impact of the difference in the number of reads between samples, the alpha rarefaction curve was plotted with 5000 sampling depths. For taxonomic classification, the “Greengenes 13_8 99% OTUs” dataset^52^ was utilized as 16S rRNA gene databases. In order to make a comparison excluding bacteria with low frequency of appearance, we analyzed the readings with low frequency of appearance less than 1,500 times (–p-min-frequency 1500). The beta diversity metric is an estimation of the between-sample diversity of the microbial profile and it was calculated by the QIIME 2 “diversity beta-group-significance” script. The PCoA analysis of has been done with the qiime2R scripts (qiime2R: Importing QIIME2 artifacts and associated data into R sessions. Jordan E Bisanz (2018) https://github.com/jbisanz/qiime2R.) Statistical analyses for diversity metrics and ANCOM were also done through QIIME 2. Box plots in a Qurro visualization are generated by Altair-4.1.0^53^ with vega-datasets-0.9.0^54^ in Qurro’s Python code.

Linear discriminant analysis Effect Size (LefSe) was used to identify OTUs that explain differences between treatments using Kruskal–Wallis tests and a Linear Discriminant Analysis (LDA) threshold of 3.0^55^. p-values below 0.05 were considered significant. For functional inferences of the microbial community, we conducted Phylogenetic Investigation of Communities by Reconstruction of Unobserved States 2 (PICRUSt2) (v2.2.0-b)^56^ with ASVs according to the instructions published at https://github.com/picrust/picrust2/wiki. PICRUSt2 predictions were supported by Enzyme Classification numbers (EC numbers) and COGs—Clusters of Orthologous Groups (as of 13 Dec 2021). We generated PICRUSt2 EC and COG gene family predictions. The results were visualized in statistical analysis of taxonomic and functional profiles (STAMP) version 2.1.3^57^ and tested using Welch’s *t* test for two groups of species. All predictions were corrected for multiple testing (Benjamini–Hochberg method, FDR *q* < 0.05).

### Isolation of intestinal bacteria from the gastrointestinal tract of *Andamia tetradactylus* and *Entomacrodus stellifer*

Isolation of bacteria from *A. tetradactylum* was carried out on 15th December in 2017 and 3rd April in 2018, and it was on 20th July and 6th August in 2018 for *E. stellifer.* The gastrointestinal tracts were processed as follows. The gastrointestinal tract was finely chopped with scissors and suspended in 10 mL of seawater diluent and vortexed. 100 µL of them was spread in LB and Zobell solid media. They were incubated at 25 °C for about 2–7 days. Several colonies of distinct color and colony shape were isolated. The isolated strains were stocked in LB 15% Glycerol medium or Zobell 15% Glycerol medium at − 80 °C.

As a result of bacterial isolation, a total of 78 bacterial strains were isolated including 20 and 14 strains in the LB and Zobell media, respectively, from *A. tetradactylus,* and 24 and 20 strains in the LB and Zobell media, respectively, from *E. stellifer*. From these strains, genome DNA was extracted and a region of 16S rDNA was amplified to determine the species using the following primer set (5′-AGAGTTTGATCCTGGCTCAG-3′ and 5′-GGTTACCTTGTTACGACTT-3′). A phylogenetic tree was constructed by the ClustalW program (DDBJ, Japan) and visualized by FigTree^58^.

### Genome sequencing

Genomic DNAs of YZOS-03 isolated from *A. tetradactylus* and HLBS-07 strain isolated from *E. stellifer* were purified by zirconia bead crushing and subsequent ethanol precipitation. Sequencing libraries were prepared using the Rapid Sequencing Kit (SQK-RAD004, Oxford Nanopore Technologies (ONT)) from unfragmented genomic DNA (approximately 400 ng input). Long-read sequencing was performed using the minION platform with an R9.4.1 flow cell (FLO-MIN106). Sequencing quality was monitored with the MinKNOW interface 19.12.5. After 24 h sequencing output fast5 files were basecalled using the Guppy software v3.6.0 (via docker GenomicParisCentre/guppy-gpu^59^).

For the assembly process, a total of 1.0 Gbps reads were quality-filtered and subsampled by Filtlong v0.2.0 to filter by lengths of 5000 bp (-min_length 5000) and quality of 1 (-min_mean_q 90), resulting in around 200× expected coverage. The subsampled reads were assembled with Canu v2.0^60^ or Flye v.2.7.1^61^. Consensus assembly was called using MarginPolish v1.3.dev-5492204^62^ or Racon (four times repetition^63^) with a total of raw reads, followed by Medaka v0.11.5, sequence correction software provided by ONT Research (via docker nanozoo/medaka, https://github.com/nanoporetech/medaka). Completeness of the assemblies of each step was tested with BUSCO v4.6.0 with lineage option “vibrionales_odb10”. Annotation was performed using DFAST v. 1.2.4 (2019.11.7) from the DDBJ^64^. Circular visualization of the genomes was carried out in the CGview server v1.0^65^.

## Supplementary Information

Supplementary Information.

## Data Availability

The MiSeq fastq reads for Flora analysis are available in the DRA under the accession number DRA011079. The chromosome sequences were deposited in DDBJ under the accession numbers AP023185 and AP023186 for YZOS-03, and AP023187 and AP023188 for HLBS-07. Raw ONT reads are also available in the DRA under the accession number DRA010239 and the BioProject accession number PRJDB9917 for YZOS-03, and DRA010240 and the BioProject accession number PRJDB9917 for HLBS-07.

## References

[1] Ramotar K, Conly JM, Chubb H, Louie TJ (1984). Production of menaquinones by intestinal anaerobes. J. Infect. Dis..

[2] Conly JM, Stein K (1992). The production of menaquinones (vitamin K2) by intestinal bacteria and their role in maintaining coagulation homeostasis. Prog. Food Nutr. Sci..

[3] Duttaroy AK, Duttaroy AK (2021). Chapter 9—Polysaccharides on the gut microbiome and epigenome. Evidence-Based Nutrition and Clinical Evidence of Bioactive Foods in Human Health and Disease.

[4] Hehemann JH, Correc G, Barbeyron T, Helbert W, Czjzek M, Michel G (2010). Transfer of carbohydrate-active enzymes from marine bacteria to Japanese gut microbiota. Nature.

[5] Roeselers G (2011). Evidence for a core gut microbiota in the zebrafish. ISME J..

[6] Sullam KE (2012). Environmental and ecological factors that shape the gut bacterial communities of fish: A meta-analysis. Mol. Ecol..

[7] Wu S, Wang G, Angert ER, Wang W, Li W, Zou H (2012). Composition, diversity, and origin of the bacterial community in grass carp intestine. PLoS ONE.

[8] Lai KP (2020). Osmotic stress induces gut microbiota community shift in fish. Environ. Microbiol..

[9] Akiyoshi H, Inoue A (2004). Comparative histological study of teleost livers in relation to phylogeny. Zool. Sci..

[10] Kimura S (2010). New Atlas of Fish Anatomy.

[11] Wilson JM, Castro LFC, Grosell M, Farrell AP, Braunaer CJ (2010). Morphological diversity of the gastrointestinal tract in fishes. Fish Physiology: The Multifunctional Gut of Fish.

[12] Hossain AM, Dutta HM, Munshi JSD, Dutta HM (1996). Phylogeny, ontogeny, structure and function of digestive tract appendages (caeca) in teleost fish. Fish Morphology Horizon of New Research.

[13] Kent GC, Carr RK, Kent GC, Carr RK (2000). Digestive system. Comparative Anatomy of the Vertebrates.

[14] Romer AS, Parsons TS, Romer AS, Parsons TS (2007). Digestive system. The Vertebrate Body.

[15] López Nadal A, Ikeda-Ohtsubo W, Sipkema D, Peggs D, McGurk C, Forlenza M, Wiegertjes GF, Brugman S (2020). Feed, microbiota, and Gut immunity: Using the zebrafish model to understand fish health. Front. Immunol..

[16] Robinson CD (2018). Experimental bacterial adaptation to the zebrafish gut reveals a primary role for immigration. PLoS Biol..

[17] Rawls JF, Mahowald MA, Ley RE, Gordon JI (2006). Reciprocal gut microbiota transplants from zebrafish and mice to germ-free recipients reveal host habitat selection. Cell.

[18] Kawamoto H (2006). Cloning and sequencing analysis of alginate lyase genes from the marine bacterium *Vibrio* sp. O2. Mar. Biotechnol. (New York).

[19] Dharani SR, Srinivasan R, Sarath R, Ramya M (2020). Recent progress on engineering microbial alginate lyases towards their versatile role in biotechnological applications. Folia Microbiol..

[20] Park J-K (1997). Molecular cloning, nucleotide sequencing, and regulation of the chiA gene encoding one of chitinases from *Enterobacter* sp. G-1. J Ferment Bioeng..

[21] Amakata D (2005). *Mitsuaria chitosanitabida* gen. nov., sp. Nov., an aerobic, chitosanase-producing member of the 'Betaproteobacteria'. Int. J. Syst. Evol. Microbiol..

[22] Kawamukai M (2018). Biosynthesis and applications of prenylquinones. Biosci. Biotechnol. Biochem..

[23] Ghanbari M, Kneifel W, Domig KJ (2015). A new view of the fish gut microbiome: Advances from next-generation sequencing. Aquaculture.

[24] Talwar C, Nagar S, Lal R, Negi RK (2018). Fish gut microbiome: Current approaches and future perspectives. Indian J. Microbiol..

[25] Ray AK, Ghosh K, Ringø E (2012). Enzyme-producing bacteria isolated from fish gut: A review. Aquac. Nutr..

[26] Clements KD, Pasch IBY, Moran D, Turner SJ (2007). Clostridia dominate 16S rRNA gene libraries prepared from the hindgut of temperate marine herbivorous fishes. Mar. Biol..

[27] Clements KD, Choat JH (1995). Fermentation in tropical marine herbivorous fishes. Physiol. Zool..

[28] Le MH, Wang D (2020). Structure and membership of gut microbial communities in multiple fish cryptic species under potential migratory effects. Sci. Rep..

[29] Givens CE, Ransom B, Bano N, Hollibaugh JT (2015). Comparison of the gut microbiomes of 12 bony fish and 3 shark species. Mar. Ecol. Prog. Ser..

[30] Piazzon MC, Naya-Català F, Simó-Mirabet P, Picard-Sánchez A, Roig FJ, Calduch-Giner JA, Sitjà-Bobadilla A, Pérez-Sánchez J (2019). Sex, age, and bacteria: How the intestinal microbiota is modulated in a protandrous hermaphrodite fish. Front. Microbiol..

[31] Li Z (2020). Puffer fish gut microbiota studies revealed unique bacterial co-occurrence patterns and new insights on tetrodotoxin producers. Mar. Drugs.

[32] Méndez-Pérez R (2020). High-throughput sequencing of the 16S rRNA gene to analyze the gut microbiome in juvenile and adult tropical gar (*Atractosteus tropicus*). Lat. Am. J. Aquat. Res..

[33] van Kessel MA (2011). Pyrosequencing of 16S rRNA gene amplicons to study the microbiota in the gastrointestinal tract of carp (*Cyprinus carpio* L.). AMB Express.

[34] Di Maiuta N, Schwarzentruber P, Schenker M, Schoelkopf J (2013). Microbial population dynamics in the faeces of wood-eating loricariid catfishes. Lett. Appl. Microbiol..

[35] Bennett KW, Eley A (1993). Fusobacteria: New taxonomy and related diseases. J. Med. Microbiol..

[36] Titus E, Ahearn GA (1988). Short-chain fatty acid transport in the intestine of a herbivorous teleost. J. Exp. Biol..

[37] von Engelhardt W, Bartels J, Kirschberger S, Meyer-zu-Düttingdorf HD, Busche R (1998). Role of short-chain fatty acids in the hind gut. Vet. Q..

[38] Clements KD, Gleeson VP, Slaytor M (1994). Short-chain fatty acid metabolism in temperate marine herbivorous fish. J. Comp. Physiol. B.

[39] Nuez-Ortin, W. G., Prado, S. & Toranzo, A. E. Antimicrobial properties of butyric acid and other organic acids against pathogenic bacteria affecting the main aquatic species. In *Conference proceedings aqua conference 2012, Prague, Czech Republic* (2012).

[40] Llewellyn MS (2016). The biogeography of the atlantic salmon (*Salmo salar*) gut microbiome. ISME J..

[41] McCarter LL (2001). Polar flagellar motility of the Vibrionaceae. Microbiol. Mol. Biol. Rev..

[42] Lu R (2021). The quorum sensing regulator OpaR is a repressor of polar flagellum genes in *Vibrio parahaemolyticus*. J. Microbiol. (Seoul)..

[43] Gibbs KA, Urbanowski ML, Greenberg EP (2008). Genetic determinants of self identity and social recognition in bacteria. Science (New York).

[44] Liu XF, Cao Y, Zhang HL, Chen YJ, Hu CJ (2015). Complete genome sequence of *Vibrio alginolyticus* ATCC 17749T. Genome Announc..

[45] Bhotra T, Singh DV (2016). Whole-genome sequence of *Vibrio alginolyticus* isolated from the Mucus of the Coral Fungia danai in the Andaman Sea, India. Genome Announc..

[46] Deng Y, Chen C, Zhao Z, Huang X, Yang Y, Ding X (2016). Complete genome sequence of *Vibrio alginolyticus* ZJ-T. Genome Announc..

[47] Wang P, Wen Z, Li B, Zeng Z, Wang X (2016). Complete genome sequence of *Vibrio alginolyticus* ATCC 33787(T) isolated from seawater with three native megaplasmids. Mar. Genomics.

[48] Gao M (2020). Characteristics and complete genome sequence of the virulent *Vibrio alginolyticus* phage VAP7, isolated in Hainan, China. Arch. Virol..

[49] Kilkenny C, Browne WJ, Cuthill IC, Emerson M, Altman DG (2010). Improving bioscience research reporting: The ARRIVE guidelines for reporting animal research. PLoS Biol..

[50] Bolyen E (2019). Reproducible, interactive, scalable and extensible microbiome data science using QIIME 2. Nat. Biotechnol..

[51] Callahan BJ, McMurdie PJ, Rosen MJ, Han AW, Johnson AJ, Holmes SP (2016). DADA2: High-resolution sample inference from Illumina amplicon data. Nat. Methods.

[52] DeSantis TZ (2006). Greengenes, a chimera-checked 16S rRNA gene database and workbench compatible with ARB. Appl. Environ. Microbiol..

[53] VanderPlas J (2018). Altair: Interactive statistical visualizations for python. J. Open Source Softw..

[54] Fedarko MW (2020). Visualizing 'omic feature rankings and log-ratios using Qurro. NAR Genomics Bioinform..

[55] Segata N, Izard J, Waldron L, Gevers D, Miropolsky L, Garrett WS, Huttenhower C (2011). Metagenomic biomarker discovery and explanation. Genome Biol..

[56] Douglas GM (2020). PICRUSt2: An improved and customizable approach for metagenome inference. BioRxiv..

[57] Parks DH, Tyson GW, Hugenholtz P, Beiko RG (2014). STAMP: Statistical analysis of taxonomic and functional profiles. Bioinformatics.

[58] Rambaut, A. *FigTree v1.4.2. Computer Program* (2009). Retrieved 4 Oct 2016, from http://tree.bio.ed.ac.uk/software/figtree/.

[59] Wick RR, Judd LM, Holt KE (2019). Performance of neural network basecalling tools for Oxford Nanopore sequencing. Genome Biol..

[60] Koren S, Walenz BP, Berlin K, Miller JR, Bergman NH, Phillippy AM (2017). Canu: Scalable and accurate long-read assembly via adaptive *k*-mer weighting and repeat separation. Genome Res..

[61] Kolmogorov M, Yuan J, Lin Y, Pevzner PA (2019). Assembly of long, error-prone reads using repeat graphs. Nat. Biotechnol..

[62] Shafin K (2020). Nanopore sequencing and the Shasta toolkit enable efficient de novo assembly of eleven human genomes. Nat. Biotechnol..

[63] Vaser R, Sović I, Nagarajan N, Šikić M (2017). Fast and accurate de novo genome assembly from long uncorrected reads. Genome Res..

[64] Tanizawa Y, Fujisawa T, Nakamura Y (2018). DFAST: A flexible prokaryotic genome annotation pipeline for faster genome publication. Bioinformatics (Oxford).

[65] Stothard P, Wishart DS (2005). Circular genome visualization and exploration using CGView. Bioinformatics (Oxford).

