# Author Correction: Gut microbiota analysis of Blenniidae fishes including an algae-eating fish and clear boundary formation among isolated *Vibrio* strains

**DOI:** 10.1038/s41598-023-36728-7

**Published:** 2023-06-19

**Authors:** Masa‑aki Yoshida, Takuma Tanabe, Hideo Akiyoshi, Makoto Kawamukai

**Affiliations:** 1 Oki Marine Biological Station, Faculty of Life and Environmental Sciences, Shimane University, Oki, Shimane Japan; 2 Faculty of Life and Environmental Sciences, Shimane University, Matsue, Shimane Japan

Correction to: *Scientific Reports* 10.1038/s41598-022-08511-7, published online 17 March 2022

The original version of this Article contained an error.

In the Introduction, the statement

"Blenniidae is unique fish that feed on algae and invertebrates; moreover, fish with algae-feeding habits are exclusively found in the Blenniidae family."

was inaccurate. To avoid misinterpretation by the readers, it was revised and now reads

"Blenniidae is unique fish that feed on algae and invertebrates."

The original Article has been corrected.

