# Supplementary material for: Gut microbiota analysis of Blenniidae fishes including an algae-eating fish and clear boundary formation among isolated Vibrio strains
Source: Sci Rep. 2022 Mar 17;12:4642. doi: 10.1038/s41598-022-08511-7 (PMC8930983; doi:10.1038/s41598-022-08511-7)
Supplement: Supplementary file 1 — Supplementary Information. [file 41598_2022_8511_MOESM1_ESM.pdf]

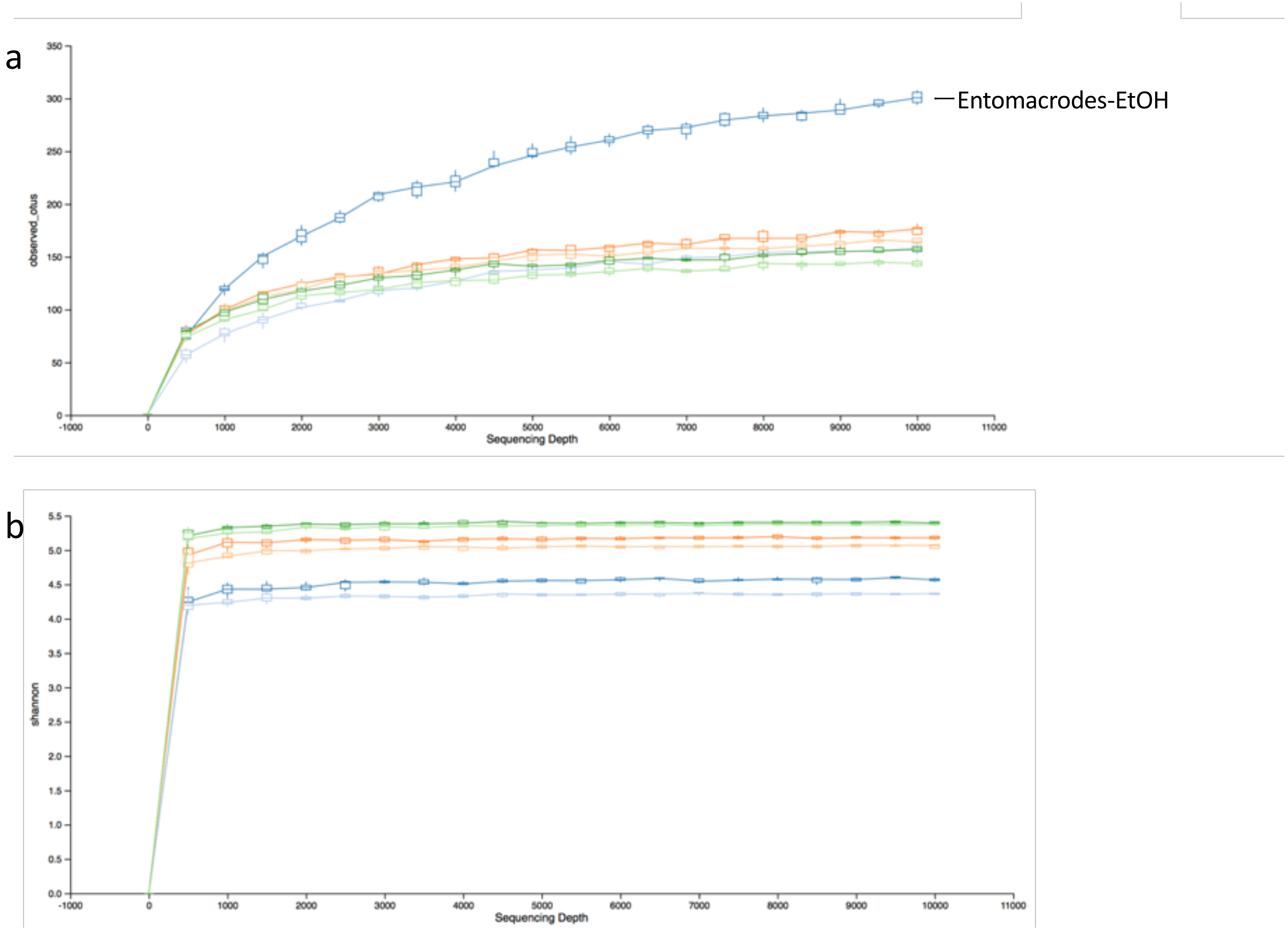

Figure S1. Alpha rarefaction curves of herbivorous fish gut microbiomes. The numbers of observed OTUs (a) and Shannon diversity (b) were plotted against read counts. Rarefaction curves were plotted at 500 sampling depths without iteration. Note that most samples reached plateau.

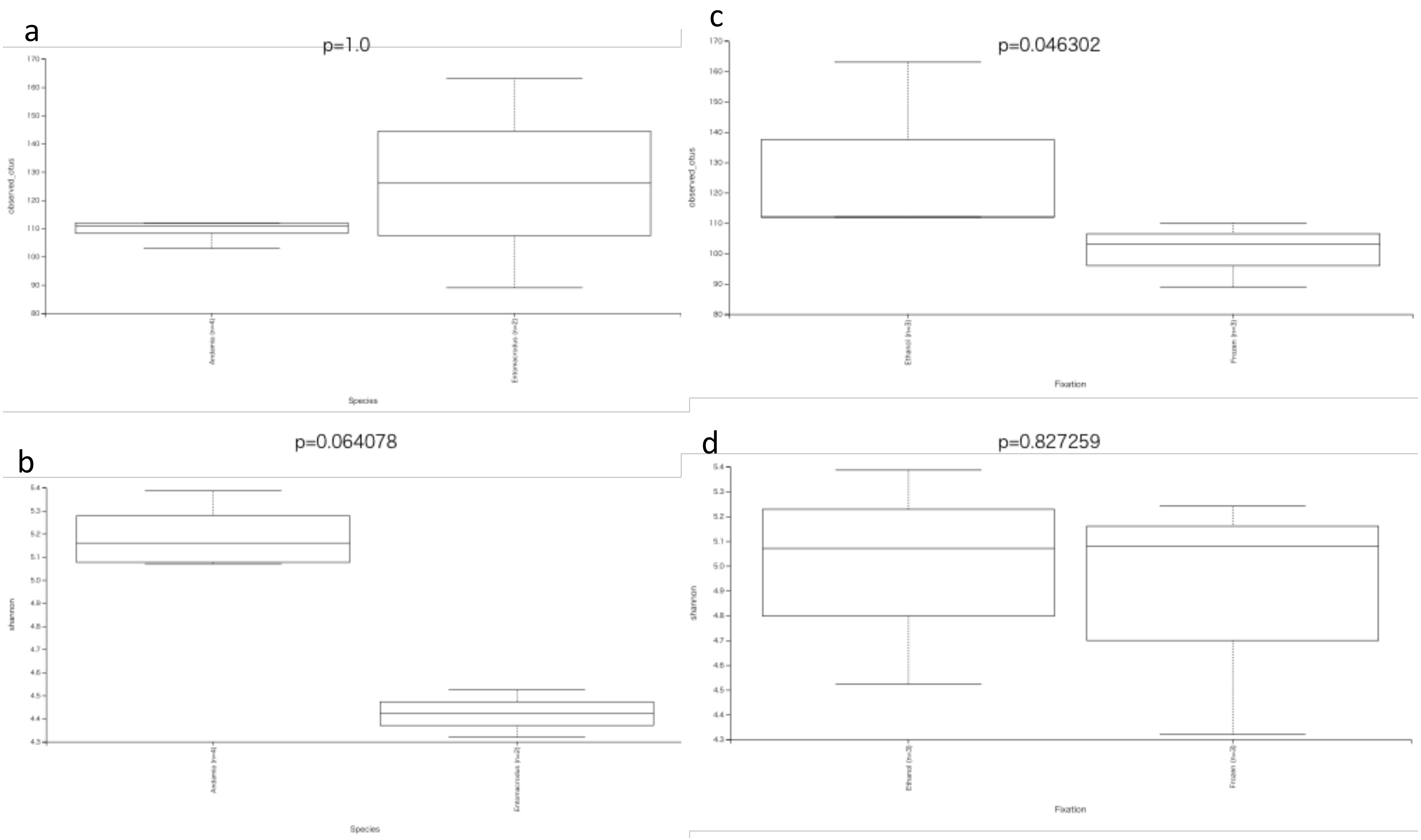

Figure S2. Comparison of alpha diversity metrics between categories of fixation (a, c) and Species (b, d). Box plots of observed OTUs (a, b) and Shannon diversity (c, d). Statistical significance was analyzed by Kruskal-Wallis test.

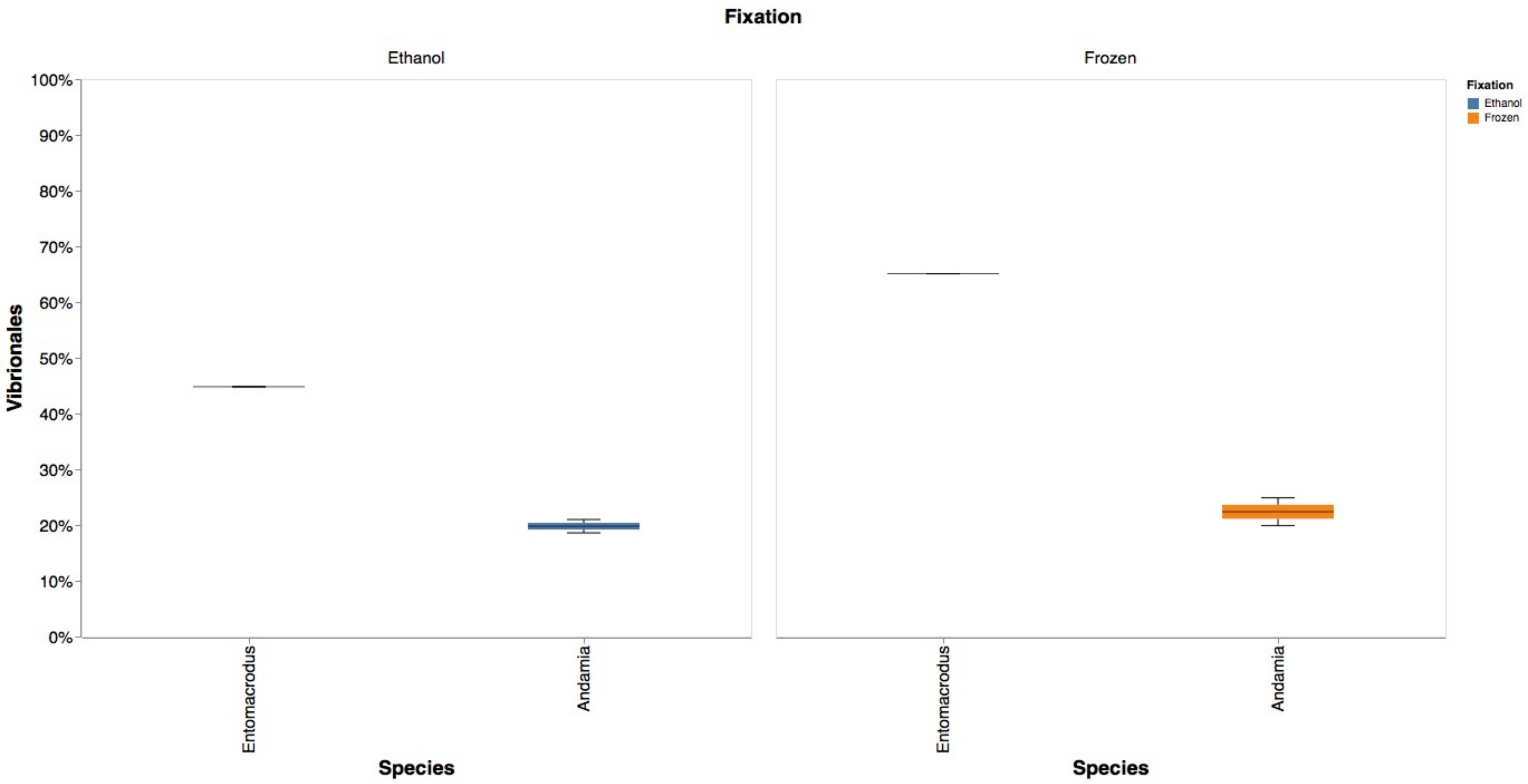

Figure S3. Box plots of the most abundant bacterial category, Vibrionales (class4).

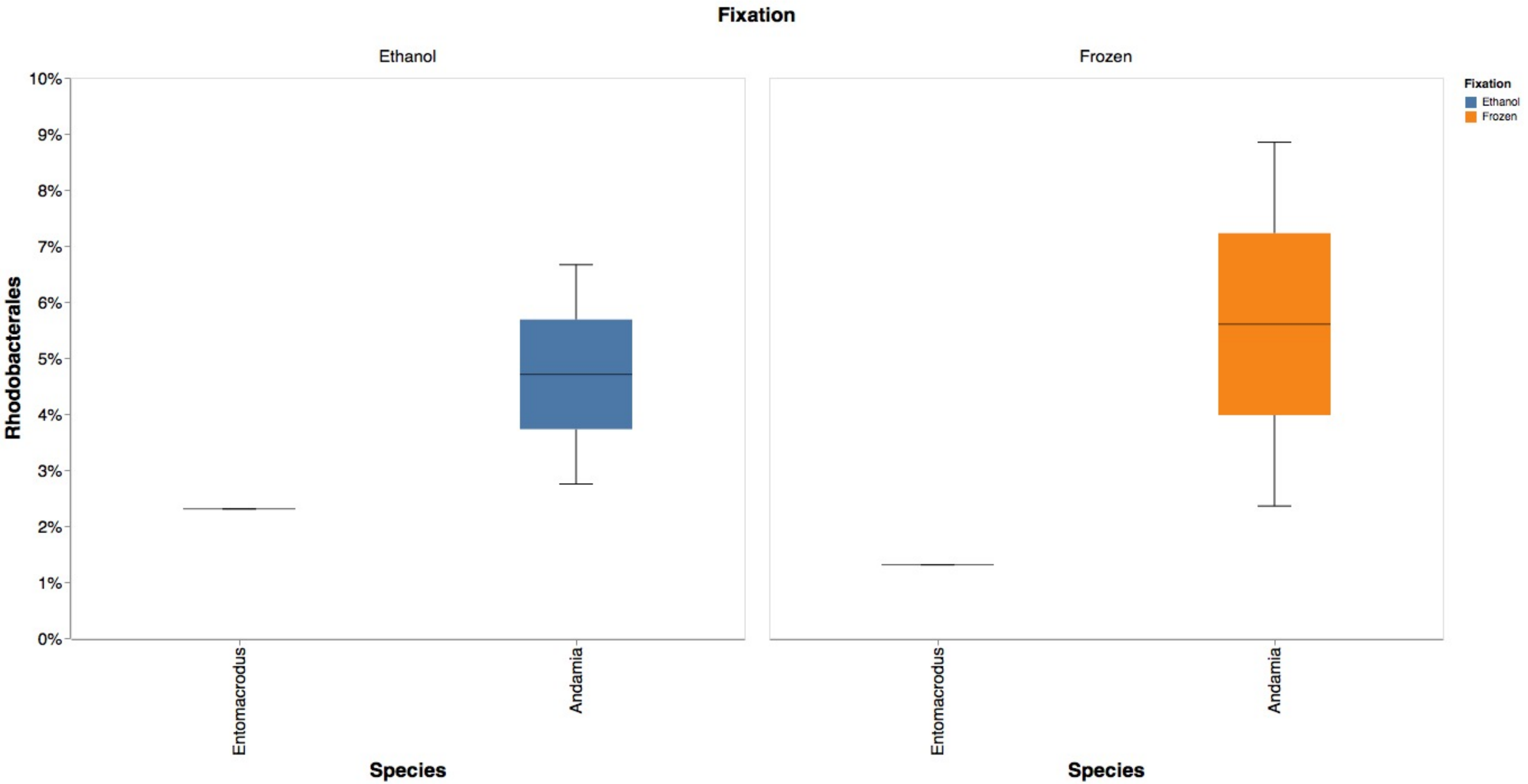

Figure S4. Box plots of highly abundant bacterial category, Rhodbacterales (class4).

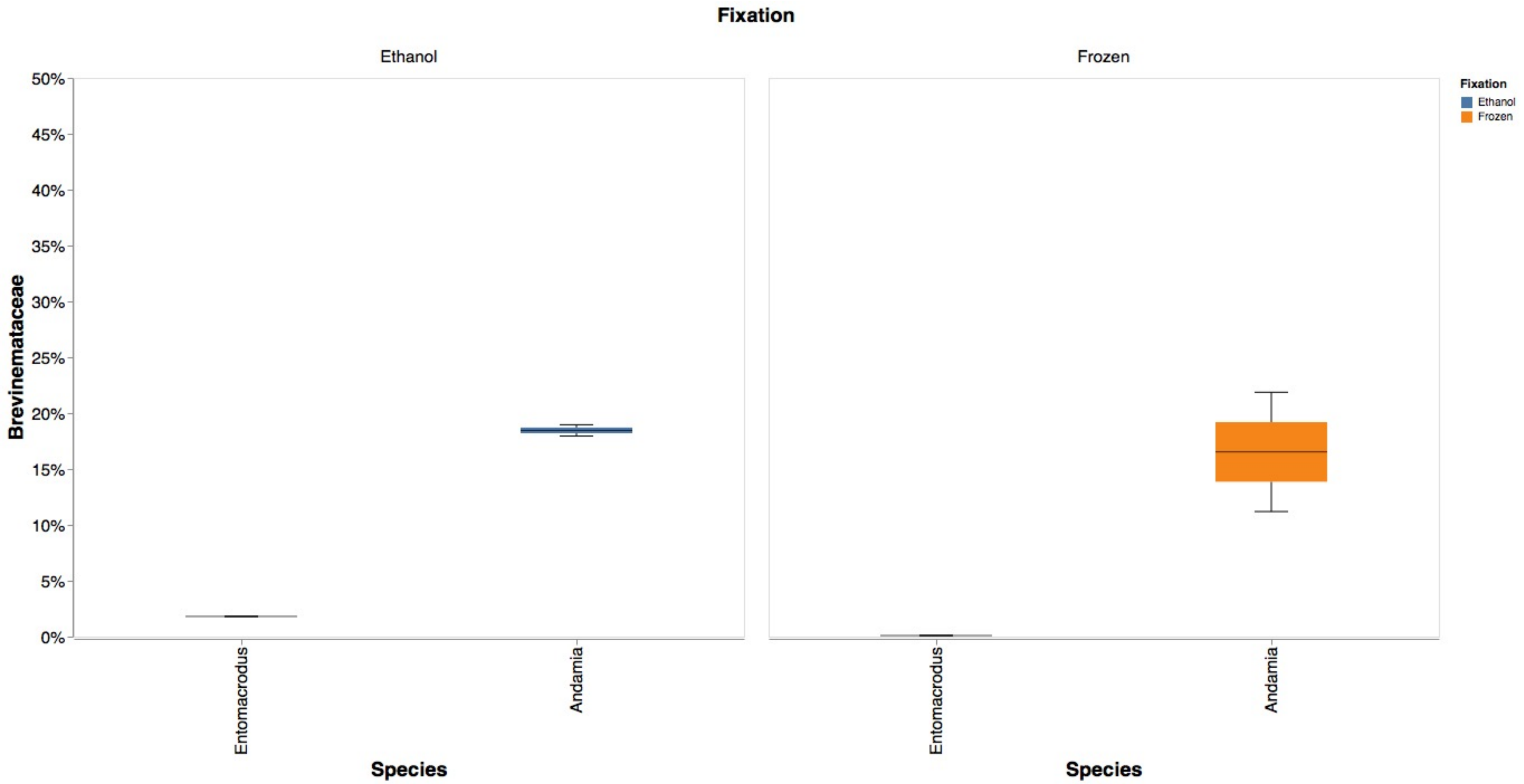

Figure S5. Box plots of specific bacterial category to *Andamia*.

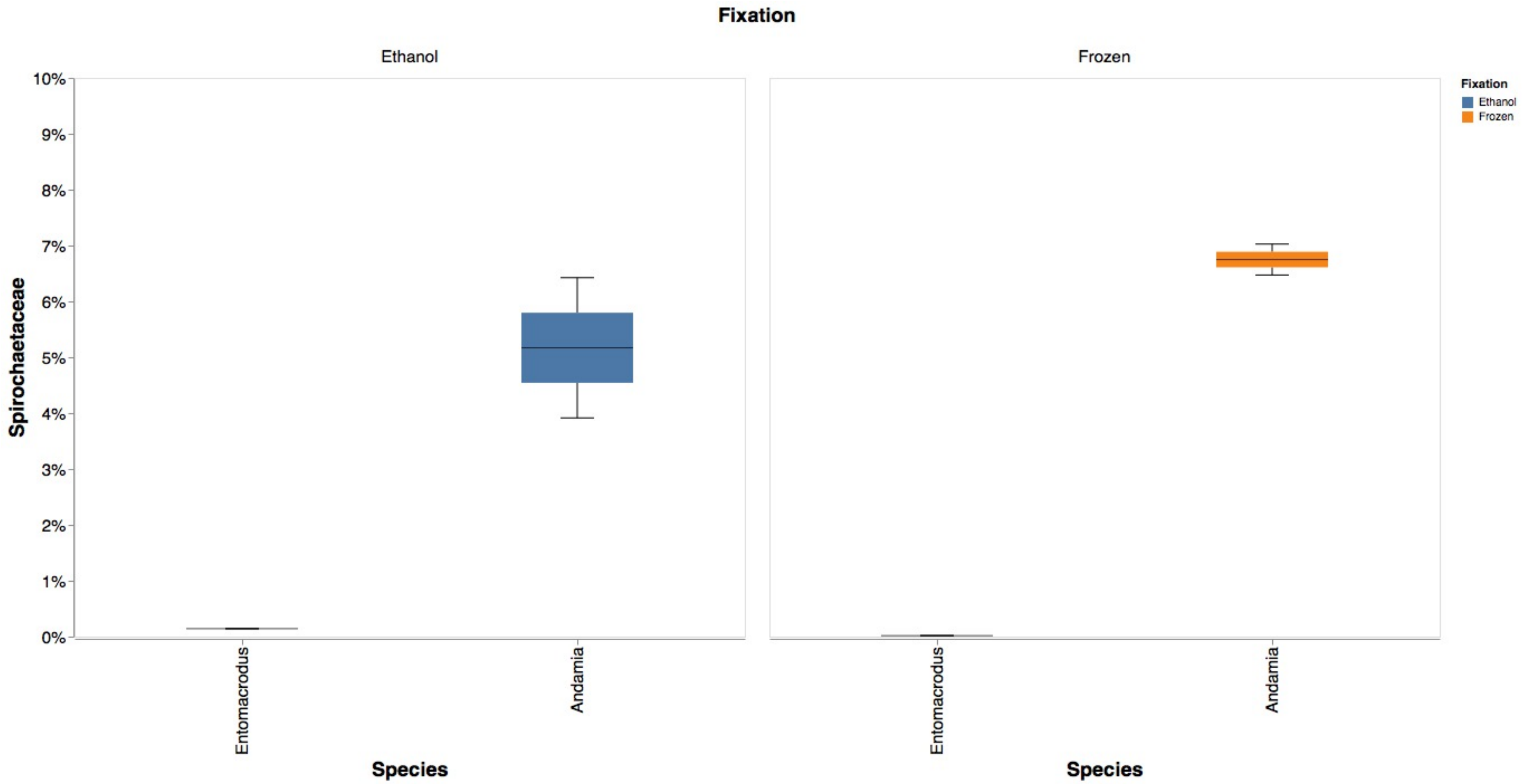

Figure S6. Box plots of specific bacterial category to *Andamia*.

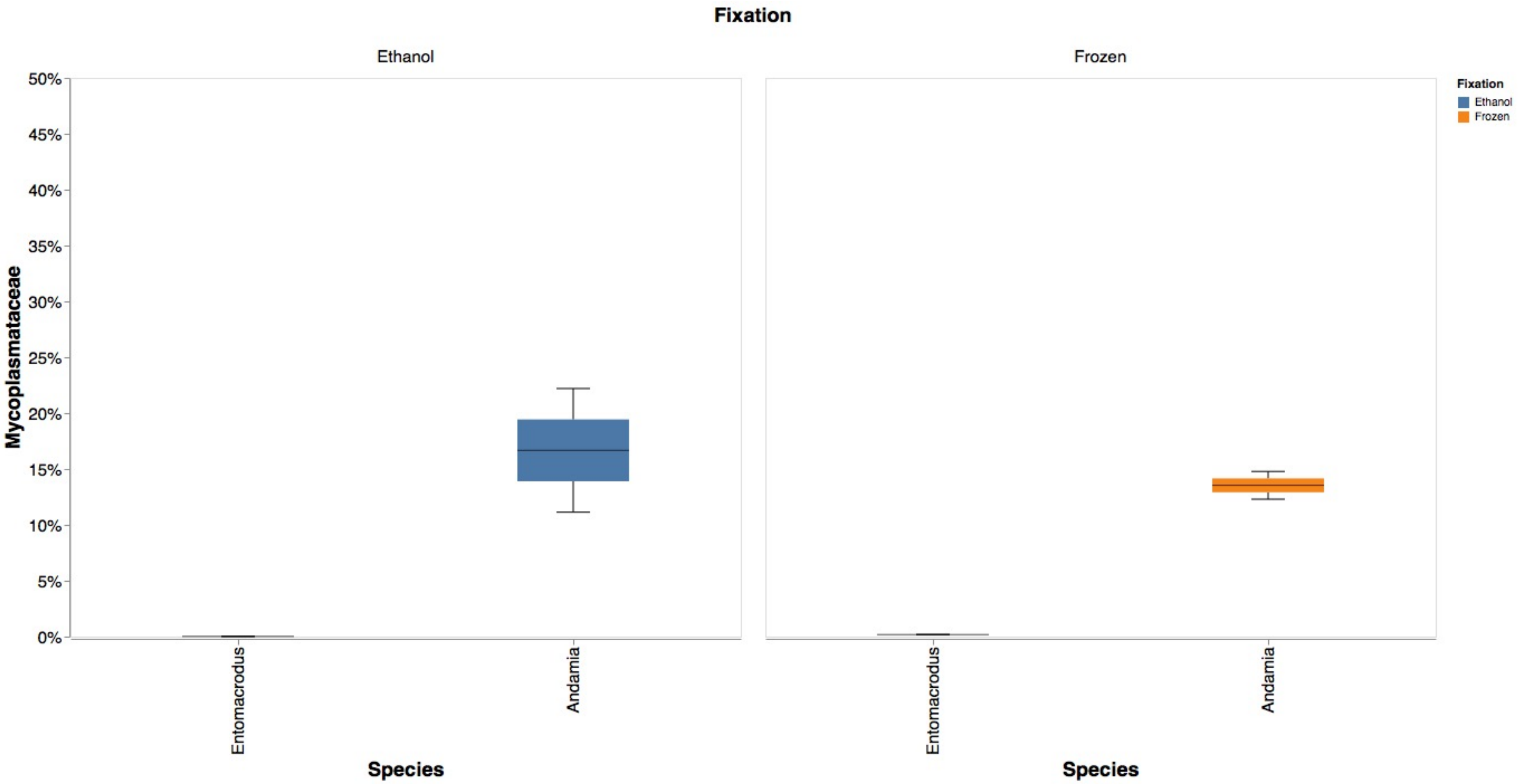

Figure S7. Box plots of specific bacterial category to *Andamia*.

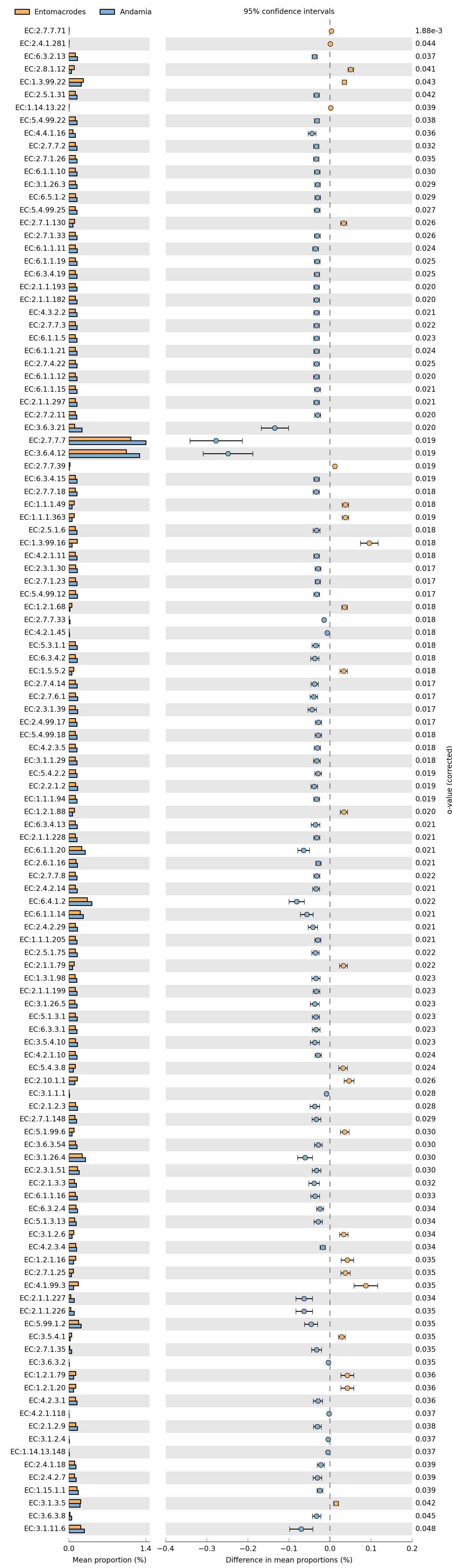

Fig.S8 Prediction of metagenome functional content correlated with two species groups using PICRUST2. Extended error bar plot for each enzymatic groups indicating differences in mean proportion for each pair of groups. Two-tailed Welch's *t* test produced a q < 0.05, which was adjusted using Benjamini-Hochberg method (FDR).

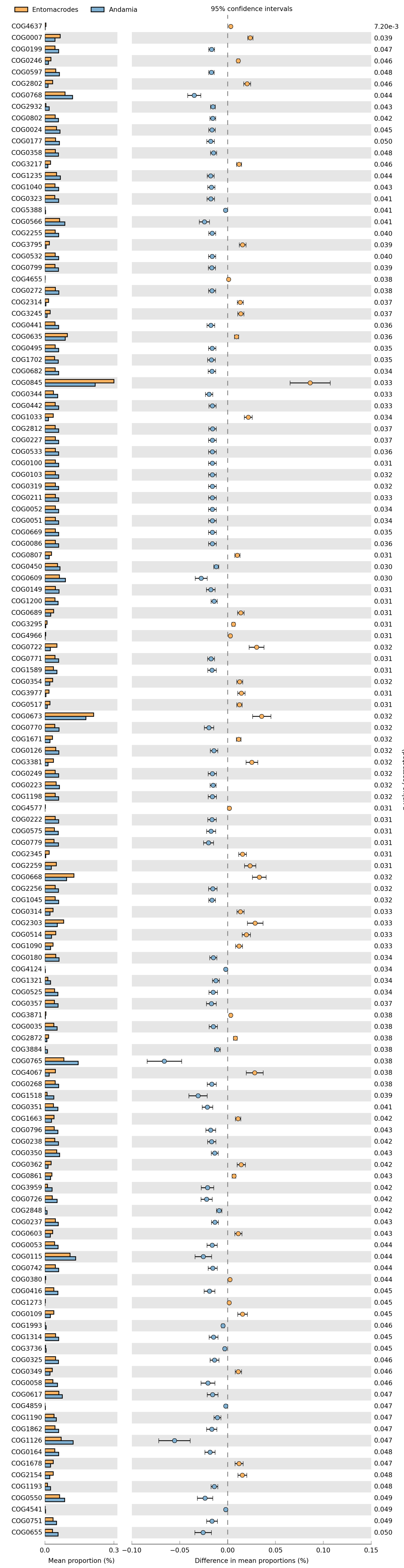

Fig.S9 Prediction of metagenome functional content correlated with two species groups using PICRUST2. Extended error bar plot for each COG (Cluster of Orthologous Groups of proteins) indicating differences in mean proportion for each pair of groups. Two-tailed Welch's *t* test produced a q < 0.05, which was adjusted using Benjamini-Hochberg method (FDR).

Supplementary Table 1

Isolation and culture from Intestinal flora of *Andamia tetradactylus*

YZOF-, YZOS- YLBF- and YLBS- means the isolated strains from *A. tetradactylus*. ZO stands for the bacterial isolates on Zobell medium and LB stands for LB media.

Strain	Species	Identity of closed species and length of determined DNA	16S rDNA sequence or DDBJ Accession number
YLBF-01	*Vibrio owensii*	99.67%,1413bp	AGCGGCGGACGGGTGAGTAATGCCTAGGAAATTGCCCTGATGTGGGGGATAACCATTGGAAA<br>CGATGGCTAATACCGCATAATGCCTACGGGCCAAAGAGGGGGACCTTCGGGCCTCTCGCGTC<br>AGGATATGCCTAGGTGGGATTAGCTAGTTGGTGAGGTAATGGCTCACCAAGGCGACGATCCC<br>TAGCTGGTCTGAGAGGATGATCAGCCACACTGGAACTGAGACACGGTCCAGACTCCTACGGG<br>AGGCAGCAGTGGGGAATATTGCACAATGGGCGCAAGCCTGATGCAGCCATGCCGCGTGTGTG<br>AAGAAGGCCTTCGGGTTGTAAAGCACTTTCAGTCGTGAGGAAGGTGGTRGWGTTAATAGCWS<br>YATYAYTTGACGTTAGCGACAGAAGAAGCACCGGCTAACTCCGTGCCAGCAGCCGCGGTAAT<br>ACGGAGGGTGCGAGCGTTAATCGGAATTACTGGGCGTAAAGCGCATGCAGGTGGTTTGTTAA<br>GTCAGATGTGAAAGCCCGGGGCTCAACCTCGGAATAGCATTTGAAACTGGCAGACTAGAGTA<br>CTGTAGAGGGGGGTAGAATTTCAGGTGTAGCGGTGAAATGCGTAGAGATCTGAAGGAATACC<br>GGTGGCGAAGGCGGCCCCCTGGACAGATACTGACACTCAGATGCGAAAGCGTGGGGAGCAA<br>ACAGGATTAGATACCCTGGTAGTCCACGCCGTAAACGATGTCTACTTGGAGGTTGTGGCCTTG<br>AGCCGTGGCTTTCGGAGCTAACGCGTTAAGTAGACCGCCTGGGGAGTACGGTCGCAAGATTA<br>AAACTCAAATGAATTGACGGGGGCCCGCACAAGCGGTGGAGCATGTGGTTTAATTCGATGCA<br>ACGCGAAGAACCTTACCTACTCTTGACATCCAGAGAACTTTCCAGAGATGGATTGGTGCCTTC<br>GGGAACTCTGAGACAGAAGCTGCATGGCTGTCGTCAGCTCGTGTTGTGAAATGTTGGGTTAA<br>GTCCCGCAACGAGCGCAACCCTTATCCTTGTTTGCCAGCGAGTAATGTCGGGAACTCCAGGG<br>AGACTGCCGGTGATAAACCGGAGGAAGGTGGGGACGACGTCAAGTCATCATGGCCCTTACGA<br>GTAGGGCTACACACGTGCTACAATGGCGCATACAGAGGGCAGCAAGCTAGCGATAGTGAGC<br>GAATCCCAAAAAGTGCGTCGTAGTCCGGATTGGAGTCTGCAACTCGACTCCATGAAGTCGGA<br>ATCGCTAGTAATCGTGGATCAGAATGCCACGGTGAATACGTTCCCGGGCCTTGTACACACCGC<br>CCGTCACACCATGGGAGTGGGCTGCAAAAGAAGTAGGTAGTTTAACCTTCGGGAGGACGCTT<br>ACCACTTTGTGGTTCATGACTGGGGTGAAGTCGTAAAAAGGTAACCCT

YLBF-02	*Vibrio owensii*	99.74%, 1411bp	AGCGGCGGACGGGTGAGTAATGCCTAGGAAATTGCCCTGATGTGGGGGATAACCATTGGAAA<br>CGATGGCTAATACCGCATAATGCCTACGGGCCAAAGAGGGGGACCTTCGGGCCTCTCGCGTC<br>AGGATATGCCTAGGTGGGATTAGCTAGTTGGTGAGGTAATGGCTCACCAAGGCGACGATCCC<br>TAGCTGGTCTGAGAGGATGATCAGCCACACTGGAACTGAGACACGGTCCAGACTCCTACGGG<br>AGGCAGCAGTGGGGAATATTGCACAATGGGCGCAAGCCTGATGCAGCCATGCCGCGTGTGTG<br>AAGAAGGCCTTCGGGTTGTAAAGCACTTTCAGTCGTGAGGAAGGTGGTRGWGTTAATAGCWS<br>YATYAYTTGACGTTAGCGACAGAAGAAGCACCGGCTAACTCCGTGCCAGCAGCCGCGGTAAT<br>ACGGAGGGTGCGAGCGTTAATCGGAATTACTGGGCGTAAAGCGCATGCAGGTGGTTTGTTAA<br>GTCAGATGTGAAAGCCCGGGGCTCAACCTCGGAATAGCATTTGAAACTGGCAGACTAGAGTA<br>CTGTAGAGGGGGGTAGAATTTCAGGTGTAGCGGTGAAATGCGTAGAGATCTGAAGGAATACC<br>GGTGGCGAAGGCGGCCCCCTGGACAGATACTGACACTCAGATGCGAAAGCGTGGGGAGCAA<br>ACAGGATTAGATACCCTGGTAGTCCACGCCGTAAACGATGTCTACTTGGAGGTTGTGGCCTTG<br>AGCCGTGGCTTTCGGAGCTAACGCGTTAAGTAGACCGCCTGGGGAGTACGGTCGCAAGATTA<br>AAACTCAAATGAATTGACGGGGGCCCGCACAAGCGGTGGAGCATGTGGTTTAATTCGATGCA<br>ACGCGAAGAACCTTACCTACTCTTGACATCCAGAGAACTTTCCAGAGATGGATTGGTGCCTTC<br>GGGAACTCTGAGACAGGTGCTGCATGGCTGTCGTCAGCTCGTGTTGTGAAATGTTGGGTTAAG<br>TCCCGCAACGAGCGCAACCCTTATCCTTGTTTGCCAGCGAGTAATGTCGGGAACTCCAGGGA<br>GACTGCCGGTGATAAACCGGAGGAAGGTGGGGACGACGTCAAGTCATCATGGCCCTTACGAG<br>TAGGGCTACACACGTGCTACAATGGCGCATACAGAGGGCAGCAAGCTAGCGATAGTGAGCG<br>AATCCCAAAAAGTGCGTCGTAGTCCGGATTGGAGTCTGCAACTCGACTCCATGAAGTCGGAA<br>TCGCTAGTAATCGTGGATCAGAATGCCACGGTGAATACGTTCCCGGGCCTTGTACACACCGCC<br>CGTCACACCATGGGAGTGGGCTGCAAAAGAAGTAGGTAGTTTAACCTTCGGGAGGACGCTTA<br>CCACTTTGTGGTTCATGACTGGGGTGAAGTCGTAAAAAGGTAACC
YLBF-04	*Vibrio ponticus*	99.47%, 1415bp	LC624710
YLBF-05	*Psudoalteromonas arabiensis*	99.33%,1325bp	LC624711
YLBF-06	*Pseudomonas aeruginosa*	99.87%,1411bp	LC624712
YLBF-07	*Pseudomonas aeruginosa*	99.87%,1411bp	*identical with YLBF-06
YLBF-08<br>YZOF-01	*Pseudomonas aeruginosa*<br>Vibrio owensii	99.60%,1411bp<br>99.92%,1324bp	LC624713<br>AGCGGCGGACGGGTGAGTAATGCCTAGGAAATTGCCCTGATGTGGGGGATAACCATTGGAAA<br>CGATGGCTAATACCGCATAATGCCTACGGGCCAAAGAGGGGGACCTTCGGGCCTCTCGCGTC<br>AGGATATGCCTAGGTGGGATTAGCTAGTTGGTGAGGTAATGGCTCACCAAGGCGACGATCCC<br>TAGCTGGTCTGAGAGGATGATCAGCCACACTGGAACTGAGACACGGTCCAGACTCCTACGGG

			AGGCAGCAGTGGGGAATATTGCACAATGGGCGCAAGCCTGATGCAGCCATGCCGCGTGTGTG<br>AAGAAGGCCTTCGGGTTGTAAAGCACTTTCAGTCGTGAGGAAGGTRGTGTAGTTAATAGCTG<br>CATTAYTTGACGTTAGCGACAGAAGAAGCACCGGCTAACTCCGTGCCAGCAGCCGCGGTAAT<br>ACGGAGGGTGCGAGCGTTAATCGGAATTACTGGGCGTAAAGCGCATGCAGGTGGTTTGTTAA<br>GTCAGATGTGAAAGCCCGGGGCTCAACCTCGGAATAGCATTTGAAACTGGCAGACTAGAGTA<br>CTGTAGAGGGGGGTAGAATTTCAGGTGTAGCGGTGAAATGCGTAGAGATCTGAAGGAATACC<br>GGTGGCGAAGGCGGCCCCCTGGACAGATACTGACACTCAGATGCGAAAGCGTGGGGAGCAA<br>ACAGGATTAGATACCCTGGTAGTCCACGCCGTAAACGATGTCTACTTGGAGGTTGTGGCCTTG<br>AGCCGTGGCTTTCGGAGCTAACGCGTTAAGTAGACCGCCTGGGGAGTACGGTCGCAAGATTA<br>AAACTCAAATGAATTGACGGGGGCCCGCACAAGCGGTGGAGCATGTGGTTTAATTCGATGCA<br>ACGCGAAGAACCTTACCTACTCTTGACATCCAGAGAACTTTCCAGAGATGGATTGGTGCCTTC<br>GGGAACTCTGAGACAGGTGCTGCATGGCTGTCGTCAGCTCGTGTTGTGAAATGTTGGGTTAAG<br>TCCCGCAACGAGCGCAACCCTTATCCTTGTTTGCCAGCGAGTAATGTCGGGAACTCCAGGGA<br>GACTGCCGGTGATAAACCGGAGGAAGGTGGGGACGACGTCAAGTCATCATGGCCCTTACGAG<br>TAGGGCTACACACGTGCTACAATGGCGCATACAGAGGGCAGCAAGCTAGCGATAGTGAGCG<br>AATCCCAAAAAGTGCGTCGTAGTCCGGATTGGAGTCTGCAACTCGACTCCATGAAGTCGGAA<br>TCGCTAGTAATCGTGGATCAGAATGCCACGGTGAATACGTTCCCGGGCCTTGTACACACCGCC<br>CGTCACACCATGGGAGTGGG
YZOF-02	*Vibrio ponticus*	99.85%,1327bp	LC624768
YZOF-04	*Phaeobacter porticola*	99.78% 1261bp	LC624714
YZOF-05	*Pseudomonas alcaliphila*	99.92%,1325bp	LC624715
YZOF-07	Vibrio owensii	99.92%, 1324bp	AGCGGCGGACGGGTGAGTAATGCCTAGGAAATTGCCCTGATGTGGGGGATAACCATTGGAAA<br>CGATGGCTAATACCGCATAATGCCTACGGGCCAAAGAGGGGGACCTTCGGGCCTCTCGCGTC<br>AGGATATGCCTAGGTGGGATTAGCTAGTTGGTGAGGTAATGGCTCACCAAGGCGACGATCCC<br>TAGCTGGTCTGAGAGGATGATCAGCCACACTGGAACTGAGACACGGTCCAGACTCCTACGGG<br>AGGCAGCAGTGGGGAATATTGCACAATGGGCGCAAGCCTGATGCAGCCATGCCGCGTGTGTG<br>AAGAAGGCCTTCGGGTTGTAAAGCACTTTCAGTCGTGAGGAAGGTRGTGTAGTTAATAGCTG<br>CATTAYTTGACGTTAGCGACAGAAGAAGCACCGGCTAACTCCGTGCCAGCAGCCGCGGTAAT<br>ACGGAGGGTGCGAGCGTTAATCGGAATTACTGGGCGTAAAGCGCATGCAGGTGGTTTGTTAA

			GTCAGATGTGAAAGCCCGGGGCTCAACCTCGGAATAGCATTTGAAACTGGCAGACTAGAGTA<br>CTGTAGAGGGGGGTAGAATTTCAGGTGTAGCGGTGAAATGCGTAGAGATCTGAAGGAATACC<br>GGTGGCGAAGGCGGCCCCCTGGACAGATACTGACACTCAGATGCGAAAGCGTGGGGAGCAA<br>ACAGGATTAGATACCCTGGTAGTCCACGCCGTAAACGATGTCTACTTGGAGGTTGTGGCCTTG<br>AGCCGTGGCTTTCGGAGCTAACGCGTTAAGTAGACCGCCTGGGGAGTACGGTCGCAAGATTA<br>AAACTCAAATGAATTGACGGGGGCCCGCACAAGCGGTGGAGCATGTGGTTTAATTCGATGCA<br>ACGCGAAGAACCTTACCTACTCTTGACATCCAGAGAACTTTCCAGAGATGGATTGGTGCCTTC<br>GGGAACTCTGAGACAGGTGCTGCATGGCTGTCGTCAGCTCGTGTTGTGAAATGTTGGGTTAAG<br>TCCCGCAACGAGCGCAACCCTTATCCTTGTTTGCCAGCGAGTAATGTCGGGAACTCCAGGGA<br>GACTGCCGGTGATAAACCGGAGGAAGGTGGGGACGACGTCAAGTCATCATGGCCCTTACGAG<br>TAGGGCTACACACGTGCTACAATGGCGCATACAGAGGGCAGCAAGCTAGCGATAGTGAGCG<br>AATCCCAAAAAGTGCGTCGTAGTCCGGATTGGAGTCTGCAACTCGACTCCATGAAGTCGGAA<br>TCGCTAGTAATCGTGGATCAGAATGCCACGGTGAATACGTTCCCGGGCCTTGTACACACCGCC<br>CGTCACACCATGGGAGTGGG
YLBS-01	*Pseudoalteromonas shioyasakiensis*	99.77%, 1297bp	LC624716
YLBS-02	*Pseudoalteromonas arabiensis*	99.93%,1360bp	LC624769
YLBS-03	*Vibrio owensii*	100%,1333bp	*identical with YLBS-04
YLBS-04	*Vibrio owensii*	100%,1363bp	LC624770
YLBS-06	*Epibacterium mobile*	99.77%,1301bp	LC624717
YLBS-08	*Shewanella piezotolerans*	98.39%,1364bp	AGCGGCGGACGGGTGAGTAATGCCTAGGTATCTGCCCAGTCGAGGGGGATAACAGTTGGAAA<br>CGACTGCTAATACCGCATACGCCCTACGGGGGAAAGGAGGGGACCTTCGGGCCTTCCGCGAT<br>TGGATGAGTCTAGGTGGGATTAGCTAGTTGGTGAGGTAATGGCTCACCAAGGCGACGATCCC<br>TAGCTGGTCTGAGAGGATGATCAGCCACACTGGAACTGAGACACGGTCCAGACTCCTACGGG<br>AGGCAGCAGTGGGGAATATTGCACAATGGGCGAAAGCCTGATGCAGCCATGCCGCGTGTGTG<br>AAGAAGGCCTTCGGGTTGTAAAGCACTTTCAGCGAGGAGGAAAGGTTAYTGGCTAATATCCA<br>RTAGCTGTGACGTTACTCGCAGAAGAAGCACCGGCTAACTTCGTGCCAGCAGCCGCGGTAAT<br>ACGAGGGGTGCAAGCGTTAATCGGAATTACTGGGCGTAAAGCGTACGCAGGCGGTTTGTTAA

			GCGAGATGTGAAAGCCCCGGGCTCAACCTGGGAACTGCATTTCGAACTGGCAAACTAGAGTC<br>TTGTAGAGGGGGGTAGAATTTCAGGTGTAGCGGTGAAATGCGTAGAGATCTGAAGGAATACC<br>GGTGGCGAAGGCGGCCCCCTGGACAAAGACTGACGCTCAGGTACGAAAGCGTGGGGAGCAA<br>ACAGGATTAGATACCCTGGTAGTCCACGCCGTAAACGATGTCTACTCGGAGTTTGGTGTCTTG<br>AACACTGGGCTCTCAAGCTAACGCATTAAGTAGACCGCCTGGGGAGTACGGCCGCAAGGTTA<br>AAACTCAAATGAATTGACGGGGGCCCGCACAAGCGGTGGAGCATGTGGTTTAATTCGATGCA<br>ACGCGAAGAACCTTACCTACTCTTGACATCCTCAGAACTTTCCAGAGATGGATTGGTGCCTTC<br>GGGAACTGAGAGACAGGTGCTGCATGGCTGTCGTCAGCTCGTGTTGTGAAATGTTGGGTTAA<br>GTCCCGCAACGAGCGCAACCCTTATCCTTATTTGCCAGCACGTAATGGTGGGAACTTTAGGGA<br>GACTGCCGGTGATAAACCGGAGGAAGGTGGGGACGACGTCAAGTCATCATGGCCCTTACGAG<br>TAGGGCTACACACGTGCTACAATGGTCGGTACAGAGGGTTGCGAAGCCGCGAGGTGGAGCTA<br>ATCTCACAAAGCCGGTCGTAGTCCGGATTGGAGTCTGCAACTCGACTCCATGAAGTCGGAAT<br>CGCTAGTAATCGTAGATCAGAATGCTACGGTGAATACGTTCCCGGGCCTTGTACACACCGCCC<br>GTCACACCATGGGAGTGGGCTGCACCAGAAGTAGATAGCTTAACCTTCGGGAGGGCGTT
YLBS-09	*Pseudoalteromonas shioyasakiensis*	98.73%,1337bp	LC624718
YLBS-10	*Pseudoalteromonas arabiensis*	99.63%,1334bp	LC624719
YLBS-11	*Alteromonas macleodii*	99.39% 1319bp	LC624720
YLBS-12	*Pseudoalteromonas arabiensis*	98.94%,1324bp	LC624721
YLBS-13	*Pseudoalteromonas arabiensis*	98.49%,1326bp	LC624722
YLBS-14	*Epibacterium mobile*	99.85%,1295bp	LC624723
YLBS-15	*Vibrio ponticus*	99.85%,1367bp	LC624724
YLBS-16	*Shewanella piezotolerans*	98.13%,1362bp	AGCGGCGGACGGGTGAGTAATGCCTAGGTATCTGCCCAGTCGAGGGGGATAACAGTTGGAAA<br>CGACTGCTAATACCGCATACGCCCTACGGGGGAAAGGAGGGGACCTTCGGGCCTTCCGCGAT<br>TGGATGAGTCTAGGTGGGATTAGCTAGTTGGTGAGGTAATGGCTCACCAAGGCGACGATCCC<br>TAGCTGGTCTGAGAGGATGATCAGCCACACTGGAACTGAGACACGGTCCAGACTCCTACGGG

			AGGCAGCAGTGGGGAATATTGCACAATGGGCGAAAGCCTGATGCAGCCATGCCGCGTGTGTG<br>AAGAAGGCCTTCGGGTTGTAAAGCACTTTCAGCGAGGAGGAAAGGTTAYTGGCTAATATCCA<br>RTAGCTGTGACGTTACTCGCAGAAGAAGCACCGGCTAACTTCGTGCCAGCAGCCGCGGTAAT<br>ACGAGGGGTGCAAGCGTTAATCGGAATTACTGGGCGTAAAGCGTACGCAGGCGGTTTGTTAA<br>GCGAGATGTGAAAGCCCCGGGCTCAACCTGGGAACTGCATTTCGAACTGGCAAACTAGAGTC<br>TTGTAGAGGGGGGTAGAATTTCAGGTGTAGCGGTGAAATGCGTAGAGATCTGAAGGAATACC<br>GGTGGCGAAGGCGGCCCCCTGGACAAAGACTGACGCTCAGGTACGAAAGCGTGGGGAGCAA<br>ACAGGATTAGATACCCTGGTAGTCCACGCCGTAAACGATGTCTACTCGGAGTTTGGTGTCTTG<br>AACACTGGGCTCTCAAGCTAACGCATTAAGTAGACCGCCTGGGGAGTACGGCCGCAAGGTTA<br>AAACTCAAATGAATTGACGGGGGCCCGCACAAGCGGTGGAGCATGTGGTTTAATTCGATGCA<br>ACGCGAAGAACCTTACCTACTCTTGACATCCTCAGAACTTTCCAGAGATGGATTGGTGCCTTC<br>GGGAACTGAGAGACAGGTGCTGCATGGCTGTCGTCAGCTCGTGTTGTGAAATGTTGGGTTAA<br>GTCCCGCAACGAGCGCAACCCTTATCCTTATTTGCCAGCACGTAATGGTGGGAACTTTAGGGA<br>GACTGCCGGTGATAAACCGGAGGAAGGTGGGGACGACGTCAAGTCATCATGGCCCTTACGAG<br>TAGGGCTACACACGTGCTACAATGGTCGGTACAGAGGGTTGCGAAGCCGCGAGGTGGAGCTA<br>ATCTCACAAAGCCGGTCGTAGTCCGGATTGGAGTCTGCAACTCGACTCCATGAAGTCGGAAT<br>CGCTAGTAATCGTAGATCAGAATGCTACGGTGAATACGTTCCCGGGCCTTGTACACACCGCCC<br>GTCACACCATGGGAGTGGGCTGCACCAGAAGTAGATAGCTTAACCTTCGGGAGGGCG
YLBS-17	*Shewanella piezotolerans*	98.31%,1362bp	LC624725
YZOS-01	*Vibrio alginolyticus*	100%,1363bp	AGCGGCGGACGGGTGAGTAATGCCTAGGAAATTGCCCTGATGTGGGGGATAACCATTGGAAA<br>CGATGGCTAATACCGCATGATGCCTACGGGCCAAAGAGGGGGACCTTCGGGCCTCTCGCGTC<br>AGGATATGCCTAGGTGGGATTAGCTAGTTGGTGAGGTAAGGGCTCACCAAGGCGACGATCCC<br>TAGCTGGTCTGAGAGGATGATCAGCCACACTGGAACTGAGACACGGTCCAGACTCCTACGGG<br>AGGCAGCAGTGGGGAATATTGCACAATGGGCGCAAGCCTGATGCAGCCATGCCGCGTGTRTG<br>AAGAAGGCCTTCGGGTTGTAAAGYACTTTCAGTCGTGAGGAAGGYRGYGMTGTTAATAGCKG<br>CRTTRTTTGACGTTAGCGACAGAAGAAGCACCGGCTAACTCCGTGCCAGCAGCCGCGGTAAT<br>ACGGAGGGTGCGAGCGTTAATCGGAATTACTGGGCGTAAAGCGCATGCAGGTGGTTTGTTAA<br>GTCAGATGTGAAAGCCCGGGGCTCAACCTCGGAATAGCATTTGAAACTGGCAGACTAGAGTA<br>CTGTAGAGGGGGGTAGAATTTCAGGTGTAGCGGTGAAATGCGTAGAGATCTGAAGGAATACC<br>GGTGGCGAAGGCGGCCCCCTGGACAGATACTGACACTCAGATGCGAAAGCGTGGGGAGCAA<br>ACAGGATTAGATACCCTGGTAGTCCACGCCGTAAACGATGTCTACTTGGAGGTTGTGGCCTTG

			AGCCGTGGCTTTCGGAGCTAACGCGTTAAGTAGACCGCCTGGGGAGTACGGTCGCAAGATTA<br>AAACTCAAATGAATTGACGGGGGCCCGCACAAGCGGTGGAGCATGTGGTTTAATTCGATGCA<br>ACGCGAAGAACCTTACCTACTCTTGACATCCAGAGAACTTTCCAGAGATGGATTGGTGCCTTC<br>GGGAACTCTGAGACAGGTGCTGCATGGCTGTCGTCAGCTCGTGTTGTGAAATGTTGGGTTAAG<br>TCCCGCAACGAGCGCAACCCTTATCCTTGTTTGCCAGCGAGTAATGTCGGGAACTCCAGGGA<br>GACTGCCGGTGATAAACCGGAGGAAGGTGGGGACGACGTCAAGTCATCATGGCCCTTACGAG<br>TAGGGCTACACACGTGCTACAATGGCGCATACAGAGGGCGGCCAACTTGCGAAAGTGAGCGA<br>ATCCCAAAAAGTGCGTCGTAGTCCGGATTGGAGTCTGCAACTCGACTCCATGAAGTCGGAAT<br>CGCTAGTAATCGTGGATCAGAATGCCACGGTGAATACGTTCCCGGGCCTTGTACACACCGCCC<br>GTCACACCATGGGAGTGGGCTGCAAAAGAAGTAGGTAGTTTAACCTTCGGGGGGACGC
YZOS-02	*Vibrio alginolyticus*	99.92%,1304bp	>YZOS-02 Vibrio alginolyticus<br>AGCGGCGGACGGGTGAGTAATGCCTAGGAAATTGCCCTGATGTGGGGGATAACCATTGGAAA<br>CGATGGCTAATACCGCATGATGCCTACGGGCCAAAGAGGGGGACCTTCGGGCCTCTCGCGTC<br>AGGATATGCCTAGGTGGGATTAGCTAGTTGGTGAGGTAAGGGCTCACCAAGGCGACGATCCC<br>TAGCTGGTCTGAGAGGATGATCAGCCACACTGGAACTGAGACACGGTCCAGACTCCTACGGG<br>AGGCAGCAGTGGGGAATATTGCACAATGGGCGCAAGCCTGATGCAGCCATGCCGCGTGTRTG<br>AAGAAGGCCTTCGGGTTGTAAAGYACTTTCAGTCGTGAGGAAGGTRGTRKWGTTAATAGCRS<br>YATYATTTGACGTTAGCGACAGAAGAAGCACCGGCTAACTCCGTGCCAGCAGCCGCGGTAAT<br>ACGGAGGGTGCGAGCGTTAATCGGAATTACTGGGCGTAAAGCGCATGCAGGTGGTTTGTTAA<br>GTCAGATGTGAAAGCCCGGGGCTCAACCTCGGAATAGCATTTGAAACTGGCAGACTAGAGTA<br>CTGTAGAGGGGGGTAGAATTTCAGGTGTAGCGGTGAAATGCGTAGAGATCTGAAGGAATACC<br>GGTGGCGAAGGCGGCCCCCTGGACAGATACTGACACTCAGATGCGAAAGCGTGGGGAGCAA<br>ACAGGATTAGATACCCTGGTAGTCCACGCCGTAAACGATGTCTACTTGGAGGTTGTGGCCTTG<br>AGCCGTGGCTTTCGGAGCTAACGCGTTAAGTAGACCGCCTGGGGAGTACGGTCGCAAGATTA<br>AAACTCAAATGAATTGACGGGGGCCCGCACAAGCGGTGGAGCATGTGGTTTAATTCGATGCA<br>ACGCGAAGAACCTTACCTACTCTTGACATCCAGAGAACTTTCCAGAGATGGATTGGTGCCTTC<br>GGGAACTCTGAGACAGGTGCTGCATGGCTGTCGTCAGCTCGTGTTGTGAAATGTTGGGTTAAG<br>TCCCGCAACGAGCGCAACCCTTATCCTTGTTTGCCAGCGAGTAATGTCGGGAACTCCAGGGA<br>GACTGCCGGTGATAAACCGGAGGAAGGTGGGGACGACGTCAAGTCATCATGGCCCTTACGAG<br>TAGGGCTACACACGTGCTACAATGGCGCATACAGAGGGCGGCCAACTTGCGAAAGTGAGCGA<br>ATCCCAAAAAGTGCGTCGTAGTCCGGATTGGAGTCTGCAACTCGACTCCATGAAGTCGGAAT<br>CGCTAGTAATCGTGGATCAGAATGCCACGGTGAATACGTTCCCGGGCCTTGTACACACCGCC

YZOS-03	*Vibrio alginolyticus*	99.77%,1363bp	For Genome analysis<br>AP023185 AP023186
YZOS-04	*Alteromonas macleodii*	100%, 1340bp	AGCGGCGGACGGGTGAGTAATGCCTAGGAAATTGCCCTGATGTGGGGGATAACCATTGGAAA<br>CGATGGCTAATACCGCATAATGCCTACGGGCCAAAGAGGGGGACCTTCGGGCCTCTCGCGTC<br>AGGATATGCCTAGGTGGGATTAGCTAGTTGGTGAGGTAATGGCTCACCAAGGCGACGATCCC<br>TAGCTGGTCTGAGAGGATGATCAGCCACACTGGAACTGAGACACGGTCCAGACTCCTACGGG<br>AGGCAGCAGTGGGGAATATTGCACAATGGGCGCAAGCCTGATGCAGCCATGCCGCGTGTGTG<br>AAGAAGGCCTTCGGGTTGTAAAGCACTTTCAGTCGTGAGGAAGRTRGTRKWGTTAATAGCWS<br>YATYATTTGACGTTAGCGACAGAAGAAGCACCGGCTAACTCCGTGCCAGCAGCCGCGGTAAT<br>ACGGAGGGTGCGAGCGTTAATCGGAATTACTGGGCGTAAAGCGCATGCAGGTGGTTTGTTAA<br>GTCAGATGTGAAAGCCCGGGGCTCAACCTCGGAATAGCATTTGAAACTGGCAGACTAGAGTA<br>CTGTAGAGGGGGGTAGAATTTCAGGTGTAGCGGTGAAATGCGTAGAGATCTGAAGGAATACC<br>GGTGGCGAAGGCGGCCCCCTGGACAGATACTGACACTCAGATGCGAAAGCGTGGGGAGCAA<br>ACAGGATTAGATACCCTGGTAGTCCACGCCGTAAACGATGTCTACTTGGAGGTTGTGGCCTTG<br>AGCCGTGGCTTTCGGAGCTAACGCGTTAAGTAGACCGCCTGGGGAGTACGGTCGCAAGATTA<br>AAACTCAAATGAATTGACGGGGGCCCGCACAAGCGGTGGAGCATGTGGTTTAATTCGATGCA<br>ACGCGAAGAACCTTACCTACTCTTGACATCCAGAGAACTTTCCAGAGATGGATTGGTGCCTTC<br>GGGAACTCTGAGACAGGTGCTGCATGGCTGTCGTCAGCTCGTGTTGTGAAATGTTGGGTTAAG<br>TCCCGCAACGAGCGCAACCCTTATCCTTGTTTGCCAGCGAGTAATGTCGGGAACTCCAGGGA<br>GACTGCCGGTGATAAACCGGAGGAAGGTGGGGACGACGTCAAGTCATCATGGCCCTTACGAG<br>TAGGGCTACACACGTGCTACAATGGCGCATACAGAGGGCAGCAAGCTAGCGATAGTGAGCG<br>AATCCCAAAAAGTGCGTCGTAGTCCGGATTGGAGTCTGCAACTCGACTCCATGAAGTCGGAA<br>TCGCTAGTAATCGTGGATCAGAATGCCACGGTGAATACGTTCCCGGGCCTTGTACACACCGCC<br>CGTCACACCATGGGAGTGGGCTGCAAAAGAAGTAGG
YZOS-05	*Grimontia sp.*	96.05%, 1340bp	AGCGGCGGACGGGTGAGTAATGGCTGGGAACCTGCCTGGTAGAGGGGGATAACCACTGGAA<br>ACGGTGGCTAATACCGCATGATGTCTWYGGACCAAAGAGGGGGAYCTTCGGRCCTCTCGCTA<br>CCGGATGGGCCCAGTTGGGATTAGCTAGTTGGTGRGGTAAAGGCTCACCAAGGCGACGATCC<br>CTAGCTGGTTTGAGAGGATGATCAGCCACACTGGAACTGAGACACGGTCCAGACTCCTACGG<br>GAGGCAGCAGTGGGGAATATTGCACAATGGGCGCAAGCCTGATGCAGCCATGCCGCGTGTGT<br>GAAGAAGGCCTTCGGGTTGTAAAGCACTTTCAGCCGTGAGGAAGGCATTGTATTTTATACGTG<br>CAGTGTTTGACGTTAGCGGCAGAAGAAGCACCGGCTAACTCCGTGCCAGCAGCCGCGGTAAT<br>ACGGAGGGTGCGAGCGTTAATCGGAATTACTGGGCGTAAAGCGCATGCAGGCGGTCTGTTAA

			GCAAGATGTGAAAGCCCCGGGCTTAACCTGGGAACCGCATTTTGAACTGGCAGGCTAGAGTC<br>TTGTAGAGGGGGGTAGAATTTCAGGTGTAGCGGTGAAATGCGTAGAGATCTGAAGGAATACC<br>GGTGGCGAAGGCGGCCCCCTGGACAAAGACTGACGCTCAGATGCGAAAGCGTGGGGAGCAA<br>ACAGGATTAGATACCCTGGTAGTCCACGCCGTAAACGATGTCTACTTGGGAGGCTGTAACCTT<br>GAGTTGTGGCTTTCGGAGCTAACGCGTTAAGTAGACCGCCTGGGGAGTACGGTCGCAAGATT<br>AAAACTCAAATGAATTGACGGGGGCCCGCACAAGCGGTGGAGCATGTGGTTTAATTCGATGC<br>AACGCGAAGAACCTTACCTACTCTTGACATCCAGAGAASYTTSMAGAGATKSRWKKGTGCCT<br>TCGGGAACTCTGAGACAGGTGCTGCATGGCTGTCGTCAGCTCGTGTTGTGAAATGTTGGGTTA<br>AGTCCCGCAACGAGCGCAACCCTTATCCTTGTTTGCCAGCRMGTAATGKYGGGAACTCCAGG<br>GAGACTGCCGGTGATAAACCGGAGGAAGGTGGGGACGACGTCAAGTCATCATGGCCCTTACG<br>AGTAGGGCTACACACGTGCTACAATGGCATATACAGAGGGCAGCGAGACAGCGATGTGAAG<br>CGAATCCCAGAAAGTATGTCGTAGTCCGGATTGGAGTCTGCAACTCGACTCCATGAAGTCGG<br>AATCGCTAGTAATCGTGGATCAGAATGCCACGGTGAATACGTTCCCGGGCCTTGTACACACC<br>GCCCGTCACACCATGGGAGTGGGCTGCACCAGAAGTAG
YZOS-06	*Alteromonas macleodii*	100%, 1332bp	LC624726
YZOS-07	*Alteromonas macleodii*	99.70%, 1322bp	LC624771
YZOS-08	*Pseudoalteromonas arabiensis*	99.77%,1324bp	LC624727

Supplementary Table. 2

Isolation of intestinal bacteria from the gastrointestinal tract of *Entomacrodus stellifer*

HZOF-, HZOS, HLBF- and HLBS means the isolated strains from *E. stellifer*. ZO stands for the bacterial isolates on Zobell medium and LB stands for LB media.

Strain	Species	Identity of closed species and length of determined DNA	16SrDNA (DDBJ #)
HLBF-01	*Vibrio owensii*	99.93% 1363bp	AGCGGCGGACGGGTGAGTAATGCCTAGGAAATTGCCCTGATGTGGGGGATAACCATTGGAAA<br>CGATGGCTAATACCGCATAATGCCTACGGGCCAAAGAGGGGGACCTTCGGGCCTCTCGCGTC<br>AGGATATGCCTAGGTGGGATTAGCTAGTTGGTGAGGTAATGGCTCACCAAGGCGACGATCCC<br>TAGCTGGTCTGAGAGGATGATCAGCCACACTGGAACTGAGACACGGTCCAGACTCCTACGGG<br>AGGCAGCAGTGGGGAATATTGCACAATGGGCGCAAGCCTGATGCAGCCATGCCGCGTGTGTG<br>AAGAAGGCCTTCGGGTTGTAAAGCACTTTCAGTCGTGAGGAAGGTRGTGTAGTTAATAGCTG<br>CATTAYTTGACGTTAGCGACAGAAGAAGCACCGGCTAACTCCGTGCCAGCAGCCGCGGTAAT<br>ACGGAGGGTGCGAGCGTTAATCGGAATTACTGGGCGTAAAGCGCATGCAGGTGGTTTGTTAA<br>GTCAGATGTGAAAGCCCGGGGCTCAACCTCGGAATAGCATTTGAAACTGGCAGACTAGAGTA<br>CTGTAGAGGGGGGTAGAATTTCAGGTGTAGCGGTGAAATGCGTAGAGATCTGAAGGAATACC<br>GGTGGCGAAGGCGGCCCCCTGGACAGATACTGACACTCAGATGCGAAAGCGTGGGGAGCAA<br>ACAGGATTAGATACCCTGGTAGTCCACGCCGTAAACGATGTCTACTTGGAGGTTGTGGCCTTG<br>AGCCGTGGCTTTCGGAGCTAACGCGTTAAGTAGACCGCCTGGGGAGTACGGTCGCAAGATTA<br>AAACTCAAATGAATTGACGGGGGCCCGCACAAGCGGTGGAGCATGTGGTTTAATTCGATGCA<br>ACGCGAAGAACCTTACCTACTCTTGACATCCAGAGAACTTTYCAGAGATGRATTGGTGCCTTC<br>GGGAACTCTGAGACAGGTGCTGCATGGCTGTCGTCAGCTCGTGTTGTGAAATGTTGGGTTAAG<br>TCCCGCAACGAGCGCAACCCTTATCCTTGTTTGCCAGCGAGTAATGTCGGGAACTCCAGGGA<br>GACTGCCGGTGATAAACCGGAGGAAGGTGGGGACGACGTCAAGTCATCATGGCCCTTACGAG<br>TAGGGCTACACACGTGCTACAATGGCGCATACAGAGGGCAGCAAGCTAGCGATAGTGAGCG<br>AATCCCAAAAAGTGCGTCGTAGTCCGGATTGGAGTCTGCAACTCGACTCCATGAAGTCGGAA<br>TCGCTAGTAATCGTGGATCAGAATGCCACGGTGAATACGTTCCCGGGCCTTGTACACACCGCC<br>CGTCACACCATGGGAGTGGGCTGCAAAAGAAGTAGGTAGTTTAACCTTCGGGAGGACGC
HLBF-02	*Bacillus vietnamensis*	99.19% 1365bp	LC624772

HLBF-03	*Vibrio harveyi*	99.78% 1362bp	AGCGGCGGACGGGTGAGTAATGCCTAGGAAATTGCCCTGATGTGGGGGATAACCATTGGAAA<br>CGATGGCTAATACCGCATAATACCTWCGGGTCAAAGAGGGGGACCTTCGGGCCTCTCGCGTC<br>AGGATATGCCTAGGTGGGATTAGCTAGTTGGTGAGGTAATGGCTCACCAAGGCGACGATCCC<br>TAGCTGGTCTGAGAGGATGATCAGCCACACTGGAACTGAGACACGGTCCAGACTCCTACGGG<br>AGGCAGCAGTGGGGAATATTGCACAATGGGCGCAAGCCTGATGCAGCCATGCCGCGTGTGTG<br>AAGAAGGCCTTCGGGTTGTAAAGCACTTTCAGTCGTGAGGAAGGTRGTRKWGTTAATAGCWS<br>YATYATTTGACGTTAGCGACAGAAGAAGCACCGGCTAACTCCGTGCCAGCAGCCGCGGTAAT<br>ACGGAGGGTGCGAGCGTTAATCGGAATTACTGGGCGTAAAGCGCATGCAGGTGGTTTGTTAA<br>GTCAGATGTGAAAGCCCGGGGCTCAACCTCGGAATAGCATTTGAAACTGGCAGACTAGAGTA<br>CTGTAGAGGGGGGTAGAATTTCAGGTGTAGCGGTGAAATGCGTAGAGATCTGAAGGAATACC<br>GGTGGCGAAGGCGGCCCCCTGGACAGATACTGACACTCAGATGCGAAAGCGTGGGGAGCAA<br>ACAGGATTAGATACCCTGGTAGTCCACGCCGTAAACGATGTCTACTTGGAGGTTGTGGCCTTG<br>AGCCGTGGCTTTCGGAGCTAACGCGTTAAGTAGACCGCCTGGGGAGTACGGTCGCAAGATTA<br>AAACTCAAATGAATTGACGGGGGCCCGCACAAGCGGTGGAGCATGTGGTTTAATTCGATGCA<br>ACGCGAAGAACCTTACCTACTCTTGACATCCAGAGAACTTTCCAGAGATGGATTGGTGCCTTC<br>GGGAACTCTGAGACAGGTGCTGCATGGCTGTCGTCAGCTCGTGTTGTGAAATGTTGGGTTAAG<br>TCCCGCAACGAGCGCAACCCTTATCCTTGTTTGCCAGCACTTCGGGTGGGAACTCCAGGGAGA<br>CTGCCGGTGATAAACCGGAGGAAGGTGGGGACGACGTCAAGTCATCATGGCCCTTACGAGTA<br>GGGCTACACACGTGCTACAATGGCGCATACAGAGGGCAGCCAACTTGCGAGAGTGAGCGAAT<br>CCCAAAAAGTGCGTCGTAGTCCGGATCGGAGTCTGCAACTCGACTCCGTGAAGTCGGAATCG<br>CTAGTAATCGTGGATCAGAATGCCACGGTGAATACGTTCCCGGGCCTTGTACACACCGCCCGT<br>CACACCATGGGAGTGGGCTGCAAAAGAAGTAGGTAGTTTAACCTTCGGGAGGACGC
HLBF-04	*Vibrio harveyi*	99.93% 1362bp	AGCGGCGGACGGGTGAGTAATGCCTAGGAAATTGCCCTGATGTGGGGGATAACCATTGGAAA<br>CGATGGCTAATACCGCATAATRCCTWCGGGYCAAAGAGGGGGACCTTCGGGCCTCTCGCGTC<br>AGGATATGCCTAGGTGGGATTAGCTAGTTGGTGAGGTAATGGCTCACCAAGGCGACGATCCC<br>TAGCTGGTCTGAGAGGATGATCAGCCACACTGGAACTGAGACACGGTCCAGACTCCTACGGG<br>AGGCAGCAGTGGGGAATATTGCACAATGGGCGCAAGCCTGATGCAGCCATGCCGCGTGTGTG<br>AAGAAGGCCTTCGGGTTGTAAAGCACTTTCAGTCGTGAGGAAGGTRGTRKRGTTAATAGCWS<br>YATYATTTGACGTTAGCGACAGAAGAAGCACCGGCTAACTCCGTGCCAGCAGCCGCGGTAAT<br>ACGGAGGGTGCGAGCGTTAATCGGAATTACTGGGCGTAAAGCGCATGCAGGTGGTTTGTTAA<br>GTCAGATGTGAAAGCCCGGGGCTCAACCTCGGAATAGCATTTGAAACTGGCAGACTAGAGTA<br>CTGTAGAGGGGGGTAGAATTTCAGGTGTAGCGGTGAAATGCGTAGAGATCTGAAGGAATACC

			GGTGGCGAAGGCGGCCCCCTGGACAGATACTGACACTCAGATGCGAAAGCGTGGGGAGCAA<br>ACAGGATTAGATACCCTGGTAGTCCACGCCGTAAACGATGTCTACTTGGAGGTTGTGGCCTTG<br>AGCCGTGGCTTTCGGAGCTAACGCGTTAAGTAGACCGCCTGGGGAGTACGGTCGCAAGATTA<br>AAACTCAAATGAATTGACGGGGGCCCGCACAAGCGGTGGAGCATGTGGTTTAATTCGATGCA<br>ACGCGAAGAACCTTACCTACTCTTGACATCCAGAGAAYWTTCCAGAGATGGATTRGTGCCTT<br>CGGGAACTCTGAGACAGGTGCTGCATGGCTGTCGTCAGCTCGTGTTGTGAAATGTTGGGTTAA<br>GTCCCGCAACGAGCGCAACCCTTATCCTTGTTTGCCAGCACTTCGGGTGGGAACTCCAGGGAG<br>ACTGCCGGTGATAAACCGGAGGAAGGTGGGGACGACGTCAAGTCATCATGGCCCTTACGAGT<br>AGGGCTACACACGTGCTACAATGGCGCATACAGAGGGCRGCCAACTTGCGAGAGTGAGCGA<br>ATCCCAAAAAGTGCGTCGTAGTCCGGATCGGAGTCTGCAACTCGACTCCGTGAAGTCGGAAT<br>CGCTAGTAATCGTGGATCAGAATGCCACGGTGAATACGTTCCCGGGCCTTGTACACACCGCCC<br>GTCACACCATGGGAGTGGGCTGCAAAAGAAGTAGGTAGTTTAACCTTCGGGAGGACGC
HLBF-05	*Bacillus vietnamensis*	99.48% 1343bp	CCATCAGTCAGCGGCGGACGGGTGAGAAACACGTGGGTAACCTGCCTGTAAGACTGGGATAA<br>CTCCGGGAAACCGGGGCTAATACCGGATAACTCATTTCCTCGCATGAGGAAATGTTGAAAGG<br>TGGCTTTTAGCTATCACTTACAGATGGACCCGCGGCGCATTAGCTAGTTGGTGAGRTAACGGC<br>TCACCAAGGCGACGATGCGTAGCCGACCTGAGAGGGTGATCGGCCACACTGGGACTGAGACA<br>CGGCCCAGACTCCTACGGGAGGCAGCAGTAGGGAATCTTCCGCAATGGACGAAAGTCTGACG<br>GAGCAACGCCGCGTGAGTGATGAAGGTTTTCGGATCGTAAAACTCTGTTGTTAGGGAAGAAC<br>AAGTACCGTTCGAATAGGGCGGTACCTTGACGGTACCTAACCAGAAAGCCACGGCTAACTAC<br>GTGCCAGCAGCCGCGGTAATACGTAGGTGGCAAGCGTTGTCCGGAATTATTGGGCGTAAAGC<br>GCGCGCAGGTGGTTCCTTAAGTCTGATGTGAAAGCCCACGGCTCAACCGTGGAGGGTCATTG<br>GAAACTGGGGRACTTGAGTGCAGAAGAGGAAAGTGGAATTCCAAGTGTAGCGGTGAAATGC<br>GTAGATATTTGGAGGAACACCAGTGGCGAAGGCGACTTTCTGGTCTGTAACTGACACTGAGG<br>CGCGAAAGCGTGGGGAGCAAACAGGATTAGATACCCTGGTAGTCCACGCCGTAAACGATGA<br>GTGCTAAGTGTTAGGGGGTTTCCGCCCCTTAGTGCTGCAGCTAACGCATTAAGCACTCCGCCT<br>GGGGAGTACGGTCGCAAGACTGAAACTCAAAGGAATTGACGGGGGCCCGCACAAGCGGTGG<br>AGCATGTGGTTTAATTCGAAGCAACGCGAAGAACCTTACCAGGTCTTGACATCCTCTGACAAC<br>CCTAGAGATAGGGCTTTCCCCTTCGGGGGACAGAGTGACAGGTGGTGCATGGTTGTCGTCAG<br>CTCGTGTCGTGAGATGTTGGGTTAAGTCCCGCAACGAGCGCAACCCTTGATCTTAGTTGCCAG<br>CATTCAGTTGGGCACTCTAAGATGACTGCCGGTGACAAACCGGAGGAAGGTGGGGATGACGT<br>CAAATCATCATGCCCCTTATGACCTGGGCTACACACGTGCTACAATGGACGGTACAAAGGGC<br>AGCAAGACCGCGAGGTTTAGCCAATCCCATAAAACCGTTCTCAGTTCGGATTGTAGGCTGCA

			ACTCGCCTACATGAAGCTGGAATCGCTAGTAATCGCGGATCAGCATGCCGCGGTGAATACGT<br>TCCCGGGCCTTGTACACACCGCCCGTCACACCACGAGAGT
HLBF-07	*Bacillus firmus*	99.93% 1342bp	CCCTGAAGTCAGCGGCGGACGGGTGAGAAACACGTGGGCAACCTGCCTGTAAGACTGGGATA<br>ACTCCGGGAAACCGGGGCTAATACCGGATAACTCTTTYCCTCACATGAGGRAAAGCTGAAAG<br>ATGGTTTCGGCTATCACTTACAGATGGGCCCGCGGCGCATTAGCTAGTTGGTGAGGTAACGGC<br>TCACCAAGGCCACGATGCGTAGCCGACCTGAGAGGGTGATCGGCCACACTGGGACTGAGACA<br>CGGCCCAGACTCCTACGGGAGGCAGCAGTAGGGAATCTTCCGCAATGGACGAAAGTCTGACG<br>GAGCAACGCCGCGTGAGTGATGAAGGTTTTCGGATCGTAAAACTCTGTTGTCAGGGAAGAAC<br>AAGTACCGGAGTAACTGCCGGTACCTTGACGGTACCTGACCAGAAAGCCACGGCTAACTACG<br>TGCCAGCAGCCGCGGTAATACGTAGGTGGCAAGCGTTGTCCGGAATTATTGGGCGTAAAGCG<br>CGCGCAGGCGGTTCCTTAAGTCTGATGTGAAAGCCCCCGGCTCAACCGGGGAGGGTCATTGG<br>AAACTGGGGAACTTGAGTGCAGAAGAGAAGAGTGGAATTCCACGTGTAGCGGTGAAATGCG<br>TAGAGATGTGGAGGAACACCAGTGGCGAAGGCGACTCTTTGGTCTGTAACTGACGCTGAGGC<br>GCGAAAGCGTGGGGAGCAAACAGGATTAGATACCCTGGTAGTCCACGCCGTAAACGATGAGT<br>GCTAAGTGTTAGAGGGTTTCCGCCCTTTAGTGCTGCAGCAAACGCATTAAGCACTCCGCCTGG<br>GGAGTACGGCCGCAAGGCTGAAACTCAAAGGAATTGACGGGGGCCCGCACAAGCGGTGGAG<br>CATGTGGTTTAATTCGAAGCAACGCGAAGAACCTTACCAGGTCTTGACATCTCCTGACAACCC<br>TAGAGATAGGGCGTTCCCCTTCGGGGGACAGGATGACAGGTGGTGCATGGTTGTCGTCAGCT<br>CGTGTCGTGAGATGTTGGGTTAAGTCCCGCAACGAGCGCAACCCTTGATCTTAGTTGCCAGCA<br>TTCAGTTGGGCACTCTAAGGTGACTGCCGGTGACAAACCGGAGGAAGGTGGGGATGACGTCA<br>AATCATCATGCCCCTTATGACCTGGGCTACACACGTGCTACAATGGATGGTACAAAGGGCTG<br>CRAGACCGCGAGGTTAAGCGAATCCCATAAAACCATTCTCAGTTCGGATTGCAGGCTGCAAC<br>TCGCCTGCATGAAGCCGGAATCGCTAGTAATCGCGGATCAGCATGCCGCGGTGAATACGTTC<br>CCGGGCCTTGTACACACCGCCCGTCACACCACGAGAGT
HLBF-08	*Vibrio owensii*	100% 1363bp	AGTGGCGGACGGGTGAGTAATGCCTAGGAAATTGCCCTGATGTGGGGGATAACCATTGGAAA<br>CGATGGCTAATACCGCATAATGCCTACGGGCCAAAGAGGGGGACCTTCGGGCCTCTCGCGTC<br>AGGATATGCCTAGGTGGGATTAGCTAGTTGGTGAGGTAATGGCTCACCAAGGCGACGATCCC<br>TAGCTGGTCTGAGAGGATGATCAGCCACACTGGAACTGAGACACGGTCCAGACTCCTACGGG<br>AGGCAGCAGTGGGGAATATTGCACAATGGGCGCAAGCCTGATGCAGCCATGCCGCGTGTGTG<br>AAGAAGGCCTTCGGGTTGTAAAGCACTTTCAGTCGTGAGGAAGGTRGTGTAGTKAATAGCTG<br>CATTAYTTGACGTTAGCGACAGAAGAAGCACCGGCTAACTCCGTGCCAGCAGCCGCGGTAAT<br>ACGGAGGGTGCGAGCGTTAATCGGAATTACTGGGCGTAAAGCGCATGCAGGTGGTTTGTTAA

			GTCAGATGTGAAAGCCCGGGGCTCAACCTCGGAATAGCATTTGAAACTGGCAGACTAGAGTA<br>CTGTAGAGGGGGGTAGAATTTCAGGTGTAGCGGTGAAATGCGTAGAGATCTGAAGGAATACC<br>GGTGGCGAAGGCGGCCCCCTGGACAGATACTGACACTCAGATGCGAAAGCGTGGGGAGCAA<br>ACAGGATTAGATACCCTGGTAGTCCACGCCGTAAACGATGTCTACTTGGAGGTTGTGGCCTTG<br>AGCCGTGGCTTTCGGAGCTAACGCGTTAAGTAGACCGCCTGGGGAGTACGGTCGCAAGATTA<br>AAACTCAAATGAATTGACGGGGGCCCGCACAAGCGGTGGAGCATGTGGTTTAATTCGATGCA<br>ACGCGAAGAACCTTACCTACTCTTGACATCCAGAGAACTTTCCAGAGATGGATTGGTGCCTTC<br>GGGAACTCTGAGACAGGTGCTGCATGGCTGTCGTCAGCTCGTGTTGTGAAATGTTGGGTTAAG<br>TCCCGCAACGAGCGCAACCCTTATCCTTGTTTGCCAGCGAGTAATGTCGGGAACTCCAGGGA<br>GACTGCCGGTGATAAACCGGAGGAAGGTGGGGACGACGTCAAGTCATCATGGCCCTTACGAG<br>TAGGGCTACACACGTGCTACAATGGCGCATACAGAGGGCAGCAAGCTAGCGATAGTGAGCG<br>AATCCCAAAAAGTGYGTCGTAGTCCGGATTGGAGTCTGCAACTCGACTCCATGAAGTCGGAA<br>TCGCTAGTAATCGTGGATCAGAATGCCACGGTGAATACGTTCCCGGGCCTTGTACACACCGCC<br>CGTCACACCATGGGAGTGGGCTGCAAAAGAAGTAGGTAGTTTAACCTTCGGGAGGACGC
HLBF-09	*Vibrio harveyi*	99.85% 1361bp	AGCGGCGGACGGGTGAGTAATGCCTAGGAAATTGCCCTGACGTGGGGGATAACCATTGGAAA<br>CGATGGCTAATACCGCATAAYRCCTWCGGGYCAAAGAGGGGGACCTTCGGGCCTCTCGCGTC<br>AGGATATGCCTAGGTGGGATTAGCTAGTTGGTGAGGTAATGGCTCACCAAGGCGACGATCCC<br>TAGCTGGTCTGAGAGGATGATCAGCCACACTGGAACTGAGACACGGTCCAGACTCCTACGGG<br>AGGCAGCAGTGGGGAATATTGCACAATGGGCGCAAGCCTGATGCAGCCATGCCGCGTGTGTG<br>AAGAAGGCCTTCGGGTTGTAAAGCACTTTCAGTCGTGAGGAAGGTRGTRKWGTTAATAGCWS<br>YATYATTTGACGTTAGCGACAGAAGAAGCACCGGCTAACTCCGTGCCAGCAGCCGCGGTAAT<br>ACGGAGGGTGCGAGCGTTAATCGGAATTACTGGGCGTAAAGCGCATGCAGGTGGTTTGTTAA<br>GTCAGATGTGAAAGCCCGGGGCTCAACCTCGGAATAGCATTTGAAACTGGCAGACTAGAGTA<br>CTGTAGAGGGGGGTAGAATTTCAGGTGTAGCGGTGAAATGCGTAGAGATCTGAAGGAATACC<br>GGTGGCGAAGGCGGCCCCCTGGACAGATACTGACACTCAGATGCGAAAGCGTGGGGAGCAA<br>ACAGGATTAGATACCCTGGTAGTCCACGCCGTAAACGATGTCTACTTGGAGGTTGTGGCCTTG<br>AGCCGTGGCTTTCGGAGCTAACGCGTTAAGTAGACCGCCTGGGGAGTACGGTCGCAAGATTA<br>AAACTCAAATGAATTGACGGGGGCCCGCACAAGCGGTGGAGCATGTGGTTTAATTCGATGCA<br>ACGCGAAGAACCTTACCTACTCTTGACATCCAGAGAACWTTCCAGAGATGGATTRGTGCCTT<br>CGGGAACTCTGAGACAGGTGCTGCATGGCTGTCGTCAGCTCGTGTTGTGAAATGTTGGGTTAA<br>GTCCCGCAACGAGCGCAACCCTTATCCTTGTTTGCCAGCACTTCGGGTGGGAACTCCAGGGAG<br>ACTGCCGGTGATAAACCGGAGGAAGGTGGGGACGACGTCAAGTCATCATGGCCCTTACGAGT

			AGGGCTACACACGTGCTACAATGGCGCATACAGAGGGCRGCCAACTTGCGAGAGTGAGCGA<br>ATCCCAAAAAGTGCGTCGTAGTCCGGATCGGAGTCTGCAACTCGACTCCGTGAAGTCGGAAT<br>CGCTAGTAATCGTGGATCAGAATGCCACGGTGAATACGTTCCCGGGCCTTGTACACACCGCCC<br>GTCACACCATGGGAGTGGGCTGCAAAAGAAGTAGGTAGTTTAACCTTCGGGAGGACG
HLBF-10	*Vibrio owensii*	99.93% 1363bp	AGCGGCGGACGGGTGAGTAATGCCTAGGAAATTGCCCTGATGTGGGGGATAACCATTGGAAA<br>CGATGGCTAATACCGCATAATGCCTACGGGCCAAAGAGGGGGACCTTCGGGCCTCTCGCGTC<br>AGGATATGCCTAGGTGGGATTAGCTAGTTGGTGAGGTAATGGCTCACCAAGGCGACGATCCC<br>TAGCTGGTCTGAGAGGATGATCAGCCACACTGGAACTGAGACACGGTCCAGACTCCTACGGG<br>AGGCAGCAGTGGGGAATATTGCACAATGGGCGCAAGCCTGATGCAGCCATGCCGCGTGTGTG<br>AAGAAGGCCTTCGGGTTGTAAAGCACTTTCAGTCGTGAGGAAGGTRGTRKWGTTAATAGCWS<br>YATYAYTTGACGTTAGCGACAGAAGAAGCACCGGCTAACTCCGTGCCAGCAGCCGCGGTAAT<br>ACGGAGGGTGCGAGCGTTAATCGGAATTACTGGGCGTAAAGCGCATGCAGGTGGTTTGTTAA<br>GTCAGATGTGAAAGCCCGGGGCTCAACCTCGGAATAGCATTTGAAACTGGCAGACTAGAGTA<br>CTGTAGAGGGGGGTAGAATTTCAGGTGTAGCGGTGAAATGCGTAGAGATCTGAAGGAATACC<br>GGTGGCGAAGGCGGCCCCCTGGACAGATACTGACACTCAGATGCGAAAGCGTGGGGAGCAA<br>ACAGGATTAGATACCCTGGTAGTCCACGCCGTAAACGATGTCTACTTGGAGGTTGTGGCCTTG<br>AGCCGTGGCTTTCGGAGCTAACGCGTTAAGTAGACCGCCTGGGGAGTACGGTCGCAAGATTA<br>AAACTCAAATGAATTGACGGGGGCCCGCACAAGCGGTGGAGCATGTGGTTTAATTCGATGCA<br>ACGCGAAGAACCTTACCTACTCTTGACATCCAGAGAAYTTTYCAGAGATGGATTRGTGCCTTC<br>GGGAACTCTGAGACAGGTGCTGCATGGCTGTCGTCAGCTCGTGTTGTGAAATGTTGGGTTAAG<br>TCCCGCAACGAGCGCAACCCTTATCCTTGTTTGCCAGCGAGTAATGTCGGGAACTCCAGGGA<br>GACTGCCGGTGATAAACCGGAGGAAGGTGGGGACGACGTCAAGTCATCATGGCCCTTACGAG<br>TAGGGCTACACACGTGCTACAATGGCGCATACAGAGGGCAGCAAGCTAGCGATAGTGAGCG<br>AATCCCAAAAAGTGCGTCGTAGTCCGGATTGGAGTCTGCAACTCGACTCCATGAAGTCGGAA<br>TCGCTAGTAATCGTGGATCAGAATGCCACGGTGAATACGTTCCCGGGCCTTGTACACACCGCC<br>CTTCACACCATGGGAGTGGGCTGCAAAAGAAGTAGGTAGTTTAACCTTCGGGAGGACGC
HLBF-11	*Vibrio owensii*	99.63% 1368bp	AGCGGCGGACGGGTGAGTAATGCCTAGGAAATTGCCCTGATGTGGGGGATAACCATTGGAAA<br>CGATGGCTAATACCGCATRATGCCTAYGGGCCAAAGAGGGGGACCTTCGGGCCTCTCGCGTC<br>AGGATATGCCTAGGTGGGATTAGCTAGTTGGTGAGGTAATGGCTCACCAAGGCGACGATCCC<br>TAGCTGGTCTGAGAGGATGATCAGCCACACTGGAACTGAGACACGGTCCAGACTCCTACGGG<br>AGGCAGCAGTGGGGAATATTGCACAATGGGCGCAAGCCTGATGCAGCCATGCCGCGTGTGTG<br>AAGAAGGCCTTCGGGTTGTAAAGCACTTTCAGTCGTGAGGAAGGTRGTRKWGTTAATAGCWS

			YATYAYTTGACGTTAGCGACAGAAGAAGCACCGGCTAACTCCGTGCCAGCAGCCGCGGTAATACGGAGGGTGCGAGCGTTAATCGGAATTACTGGGCGTAAAGCGCATGCAGGTGGTTTGTTAAGTCAGATGTGAAAGCCCGGGGCTCAACCTCGGAATAGCATTTGAAACTGGCAGACTAGAGTACTGTAGAGGGGGGTAGAATTTCAGGTGTAGCGGTGAAATGCGTAGAGATCTGAAGGAATACCGGTGGCGAAGGCGGCCCCCTGGACAGATACTGACACTCAGATGCGAAAGCGTGGGGAGCAAACAGGATTAGATACCCTGGTAGTCCACGCCGTAAACGATGTCTACTTGGAGGTTGTGGCCTTGAGCCGTGGCTTTCGGAGCTAACGCGTTAAGTAGACCGCCTGGGGAGTACGGTCGCAAGATTAAAACTCAAATGAATTGACGGGGGCCCGCACAAGCGGTGGAGCATGTGGTTTAATTCGATGCAACGCGAAGAACCTTACCTACTCTTGACATCCAGAGAAYTTTYCAGAGATGRATTRGTGCCTTCGGGAACTCTGAGACAGGTGCTGCATGGCTGTCGTCAGCTCGTGTTGTGAAATGTTGGGTTAAGTCCCGCAACGAGCGCAACCCTTATCCTTGTTTGCCAGCGAGTAATGTCGGGAACTCCAGGGAGACTGCCGGTGATAAACCGGAGGAAGGTGGGGACGACGTCAAGTCATCATGGCCCTTACGAGTAGGGCTACACACGTGCTACAATGGCGCATACAGAGGGCAGCAAGCTAGCGATAGTGAGCGAATCCCAAAAAGTGCGTCGTAGTCCGGATTGGAGTCTGCAACTCGACTCCATGAAGTCGGAATCGCTAGTAATCGTGGATCAGAATGCCACGGTGAATACGTTCCCGGGCCTTGTACACACCGCCCGNCACACCATGGGAGTGGGCTGCAAAAGAAGTAGGTAGTTTAACCTTCGGGAGGACGCTTACC
HLBF-13	*Vibrio owensii*	99.85% 1363bp	AGCGGCGGACGGGTGAGTAATGCCTAGGAAATTGCCCTGATGTGGGGGATAACCATTGGAAACGATGGCTAATACCGCATAATGCCTACGGGCCAAAGAGGGGGACCTTCGGGCCTCTCGCGTCAGGATATGCCTAGGTGGGATTAGCTAGTTGGTGAGGTAATGGCTCACCAAGGCGACGATCCCTAGCTGGTCTGAGAGGATGATCAGCCACACTGGAACTGAGACACGGTCCAGACTCCTACGGGAGGCAGCAGTGGGGAATATTGCACAATGGGCGCAAGCCTGATGCAGCCATGCCGCGTGTGTGAAGAAGGCCTTCGGGTTGTAAAGCACTTTCAGTCGTGAGGAAGGTRGTGTAGTTAATAGCTGCATTAYTTGACGTTAGCGACAGAAGAAGCACCGGCTAACTCCGTGCCAGCAGCCGCGGTAATACGGAGGGTGCGAGCGTTAATCGGAATTACTGGGCGTAAAGCGCATGCAGGTGGTTTGTTAAGTCAGATGTGAAAGCCCGGGGCTCAACCTCGGAATAGCATTTGAAACTGGCAGACTAGAGTACTGTAGAGGGGGGTAGAATTTCAGGTGTAGCGGTGAAATGCGTAGAGATCTGAAGGAATACCGGTGGCGAAGGCGGCCCCCTGGACAGATACTGACACTCAGATGCGAAAGCGTGGGGAGCAAACAGGATTAGATACCCTGGTAGTCCACGCCGTAAACGATGTCTACTTGGAGGTTGTGGCCTTGAGCCGTGGCTTTCGGAGCTAACGCGTTAAGTAGACCGCCTGGGGAGTACGGTCGCAAGATTAAAACTCAAATGAATTGACGGGGGCCCGCACAAGCGGTGGAGCATGTGGTTTAATTCGATGCAACGCGAAGAACCTTACCTACTCTTGACATCCAGAGAACTTTYCAGAGATGRATTGGTGCCTTC

			GGGAACTCTGAGACAGGTGCTGCATGGCTGTCGTCAGCTCGTGTTGTGAAATGTTGGGTTAAG<br>TCCCGCAACGAGCGCAACCCTTATCCTTGTTTGCCAGCGAGTAATGTCGGGAACTCCAGGGA<br>GACTGCCGGTGATAAACCGGAGGAAGGTGGGGACGACGTCAAGTCATCATGGCCCTTACGAG<br>TAGGGCTACACACGTGCTACAATGGCGCATACAGAGGGCAGCAAGCTAGCGATAGTGAGCG<br>AATCCCAAAAAGTGCGTCGTAGTCCGGATTGGAGTCTGCAACTCGACTCCATGAAGTCGGAA<br>TCGCTAGTAATCGTGGATCAGAATGCCACGGTGAATACGTTCCCGGGCCTTGTACACACCGCC<br>CGTCACACCATGGGAGTGGGCTGCAAAAGAAGTAGGTAGTTTAACCTTCGGGAGGACGC
HLBF-14	*Vibrio harveyi*	99.78% 1366bp	AGCGGCGGACGGGTGAGTAATGCCTAGGAAATTGCCCTGATGTGGGGGATAACCATTGGAAA<br>CGATGGCTAATACCGCATAAYRCCTWCGGGYCAAAGAGGGGGACCTTCGGGCCTCTCGCGTC<br>AGGATATGCCTAGGTGGGATTAGCTRGTTGGTGAGGTAATGGCTCACCAAGGCGACGATCCC<br>TAGCTGGTCTGAGAGGATGATCAGCCACACTGGAACTGAGACACGGTCCAGACTCCTACGGG<br>AGGCAGCAGTGGGGAATATTGCACAATGGGCGCAAGCCTGATGCAGCCATGCCGCGTGTGTG<br>AAGAAGGCCTTCGGGTTGTAAAGCACTTTCAGTCGTGAGGAAGGTRGTRTAGTTAATAGCWS<br>YATYATTTGACGTTAGCGACAGAAGAAGCACCGGCTAACTCCGTGCCAGCAGCCGCGGTAAT<br>ACGGAGGGTGCGAGCGTTAATCGGAATTACTGGGCGTAAAGCGCATGCAGGTGGTTTGTTAA<br>GTCAGATGTGAAAGCCCGGGGCTCAACCTCGGAATAGCATTTGAAACTGGCAGACTAGAGTA<br>CTGTAGAGGGGGGTAGAATTTCAGGTGTAGCGGTGAAATGCGTAGAGATCTGAAGGAATACC<br>GGTGGCGAAGGCGGCCCCCTGGACAGATACTGACACTCAGATGCGAAAGCGTGGGGAGCAA<br>ACAGGATTAGATACCCTGGTAGTCCACGCCGTAAACGATGTCTACTTGGAGGTTGTGGCCTTG<br>AGCCGTGGCTTTCGGAGCTAACGCGTTAAGTAGACCGCCTGGGGAGTACGGTCGCAAGATTA<br>AAACTCAAATGAATTGACGGGGGCCCGCACAAGCGGTGGAGCATGTGGTTTAATTCGATGCA<br>ACGCGAAGAACCTTACCTACTCTTGACATCCAGAGAACTTTCCAGAGATGGATTGGTGCCTTC<br>GGGAACTCTGAGACAGGTGCTGCATGGCTGTCGTCAGCTCGTGTTGTGAAATGTTGGGTTAAG<br>TCCCGCAACGAGCGCAACCCTTATCCTTGTTTGCCAGCACTTCGGGTGGGAACTCCAGGGAGA<br>CTGCCGGTGATAAACCGGAGGAAGGTGGGGACGACGTCAAGTCATCATGGCCCTTACGAGTA<br>GGGCTACACACGTGCTACAATGGCGCATACAGAGGGCRGCCAACTTGCGAGAGTGAGCGAAT<br>CCCAAAAAGTGCGTCGTAGTCCGGATCGGAGTCTGCAACTCGACTCCGTGAAGTCGGAATCG<br>CTAGTAATCGTGGATCAGAATGCCACGGTGAATACGTTCCCGGGCCTTGTACACACCGCCCGT<br>CACACCATGGGAGTGGGCTGCAAAAGAAGTAGGTAGTTTAACCTTCGGGAGGACGCTACC
HLBF-17	*Vibrio alginolyticus*	100% 1364bp	AGCGGCGGACGGGTGAGTAATGCCTAGGAAATTGCCCTGATGTGGGGGATAACCATTGGAAA<br>CGATGGCTAATACCGCATGATGCCTACGGGCCAAAGAGGGGGACCTTCGGGCCTCTCGCGTC<br>AGGATATGCCTAGGTGGGATTAGCTAGTTGGTGAGGTAAGGGCTCACCAAGGCGACGATCCC

			TAGCTGGTCTGAGAGGATGATCAGCCACACTGGAACTGAGACACGGTCCAGACTCCTACGGG AGGCAGCAGTGGGGAATATTGCACAATGGGCGCAAGCCTGATGCAGCCATGCCGCGTGTGTG AAGAAGGCCTTCGGGTTGTAAAGCACTTTCAGTCGTGAGGAAGGTRGTRTAGTTAATAGCWS YATTATTTGACGTTAGCGACAGAAGAAGCACCGGCTAACTCCGTGCCAGCAGCCGCGGTAAT ACGGAGGGTGCGAGCGTTAATCGGAATTACTGGGCGTAAAGCGCATGCAGGTGGTTTGTTAA GTCAGATGTGAAAGCCCGGGGCTCAACCTCGGAATAGCATTTGAAACTGGCAGACTAGAGTA CTGTAGAGGGGGGTAGAATTTCAGGTGTAGCGGTGAAATGCGTAGAGATCTGAAGGAATACC GGTGGCGAAGGCGGCCCCCTGGACAGATACTGACACTCAGATGCGAAAGCGTGGGGAGCAA ACAGGATTAGATACCCTGGTAGTCCACGCCGTAAACGATGTCTACTTGGAGGTTGTGGCCTTG AGCCGTGGCTTTCGGAGCTAACGCGTTAAGTAGACCGCCTGGGGAGTACGGTCGCAAGATTA AAACTCAAATGAATTGACGGGGGCCCGCACAAGCGGTGGAGCATGTGGTTTAATTCGATGCA ACGCGAAGAACCTTACCTACTCTTGACATCCAGAGAACTTTCCAGAGATGGATTGGTGCCTTC GGGAACTCTGAGACAGGTGCTGCATGGCTGTCGTCAGCTCGTGTTGTGAAATGTTGGGTTAAG TCCCGCAACGAGCGCAACCCTTATCCTTGTTTGCCAGCGAGTAATGTCGGGAACTCCAGGGA GACTGCCGGTGATAAACCGGAGGAAGGTGGGGACGACGTCAAGTCATCATGGCCCTTACGAG TAGGGCTACACACGTGCTACAATGGCGCATACAGAGGGCGGCCAACTTGCGAAAGTGAGCGA ATCCCAAAAAGTGCGTCGTAGTCCGGATTGGAGTCTGCAACTCGACTCCATGAAGTCGGAAT CGCTAGTAATCGTGGATCAGAATGCCACGGTGAATACGTTCCCGGGCCTTGTACACACCGCCC GTCACACCATGGGAGTGGGCTGCAAAAGAAGTAGGTAGTTTAACCTTCGGGAGGACGCT
HLBF-18	*Vibrio owensii*	99.56%,1363bp	AGCGGCGGACGGGTGAGTAATGCCTAGGAAATTGCCCTGATGTGGGGGATAACCATTGGAAA CGATGGCTAATACCGCATAATGCCTACGGGCCAAAGAGGGGGACCTTCGGGCCTCTCGCGTC AGGATATGCCTAGGTGGGATTAGCTAGTTGGTGAGGTAATGGCTCACCAAGGCGACGATCCC TAGCTGGTCTGAGAGGATGATCAGCCACACTGGAACTGAGACACGGTCCAGACTCCTACGGG AGGCAGCAGYGGGGAATATTGCACAATGGGCGCAAGCCTGATGCAGCCATGCCGCGTGTGTG AAGAAGGCCTTCGGGTTGTAAAGCACTTTCAGTCGTGAGGAAGGTRGTGTAGTTAATAGCTG CATTACTTGACGTTAGCGACAGAAGAAGCACCGGCTAACTCCGTGCCAGCAGCCGCGGTAAT ACGGAGGGTGCGAGCGTTAATCGGAATTACTGGGCGTAAAGCGCATGCAGGTGGTTTGTTAA GTCAGATGTGAAAGCCCGGGGCTCAACCTCGGAATAGCATTTGAAACTGGCAGACTAGAGTA CTGTAGAGGGGGGTAGAATTTCAGGTGTAGCGGTGAAATGCGTAGAGATCTGAAGGAATACC GGTGGCGAAGGCGGCCCCCTGGACAGATACTGACACTCAGATGCGAAAGCGTGGGGAGCAA ACAGGATTAGATACCCTGGTAGTCCACGCCGTAAACGATGTCTACTTGGAGGTTGTGGCCTTG AGCCGTGGCTTTCGGAGCTAACGCGTTAAGTAGACCGCCTGGGGAGTACGGTCGCAAGATTA

			AAACTCAAATGAATTGACGGGGGCCCGCACAAGCGGTGGAGCATGTGGTTTAATTCGATGCA<br>ACGCGAAGAACCTTACCTACTCTTGACATCCAGAGAACTTTTCAGAGATGGATTGGTGCCTTC<br>GGGAACTCTGAGACAGGTGCTGCATGGCTGTCGTCAGCTCGTGTTGTGAAATGTTGGGTTAAG<br>TCCCGCAACGAGCGCAACCCTTATCCTTGTTTGCCAGCGAGTAATGTCGGGAACTCCAGGGA<br>GACTGCCGGTGATAAACCGGAGGAAGGTGGGGACGACGTCAAGTCATCATGGCCCTTACGAG<br>TAGGGCTACACACGTGCTACAATGGCGCATACAGAGGGCAGCAAGCTAGCGATAGTGAGCG<br>AATCCCAAAAAGTGCGTCGTAGTCCGGATTGGAGTCTGCAACTCGACTCCATGAAGTCGGAA<br>TCGCTAGTAATCGTGGATCAGAATGCCACGGTGAATACGTTCCCGGGCCTTGTACACACCGCC<br>CGTCACACCATGGGAGTGGGCTGCAAAAGAAGTAGGTAGTTTAACCTTCGGGAGGACGC
HLBF-19	*Bacillus aquimaris*	100%,1310bp	LC624728
HZOF-03	*Photobacterium aquae*	99.85% 1326bp	AGCGGCGGACGGGTGAGTAATGCCTGGGAACATGCCTTGGTGTGGGGGATAACCATTGGAAA<br>CGATGGCTAATACCGCATAACGTCTACGGACCAAAGAGGGGGACCTTCGGGCCTCTCGCGCC<br>AAGATTGGCCCAGGTGGGATTAGCTAGTAGGTGGGGTAACGGCTCACCTAGGCGACGATCCC<br>TAGCTGGTCTGAGAGGATGATCAGCCACACTGGAACTGAGACACGGTCCAGACTCCTACGGG<br>AGGCAGCAGTGGGGAATATTGCACAATGGGGGAAACCCTGATGCAGCCATGCCGCGTGTGTG<br>AAGAAGGCCTTCGGGTTGTAAAGCACTTTCAGTAGGGAGGAAGGCAGTGTCGTTAATAGCGG<br>CATTGTTTGACGTTACCTACAGAAGAAGCACCGGCTAACTCCGTGCCAGCAGCCGCGGTAAT<br>ACGGAGGGTGCGAGCGTTAATCGGAATTACTGGGCGTAAAGCGCATGCAGGCGGCTTGTTAA<br>GCCAGATGTGAAAGCCCGGGGCTCAACCTCGGAATAGCATTTGGAACTGGCAGGCTAGAGTC<br>TTGTAGAGGGGGGTAGAATTTCAGGTGTAGCGGTGAAATGCGTAGAGATCTGAAGGAATACC<br>GGTGGCGAAGGCGGCCCCCTGGACAAAGACTGACGCTCAGATGCGAAAGCGTGGGGAGCAA<br>ACAGGATTAGATACCCTGGTAGTCCACGCCGTAAACGATGTCTACTTGGAGGCTGTTCCCTTG<br>AGGAGTGGCTTTCGGAGCTAACGCGTTAAGTAGACCGCCTGGGGAGTACGGTCGCAAGATTA<br>AAACTCAAATGAATTGACGGGGGCCCGCACAAGCGGTGGAGCATGTGGTTTAATTCGATGCA<br>ACGCGAAGAACCTTACCTACTCTTGACATCCAGAGAACTTAGCAGAGATGCTTTGGTGCCTTC<br>GGGAACTCTGAGACAGGTGCTGCATGGCTGTCGTCAGCTCGTGTTGTGAAATGTTGGGTTAAG<br>TCCCGCAACGAGCGCAACCCCTATCCTTGTTTGCCAGCRCGTAATGGYGGGAACTCCAGGGA<br>GACTGCCGGTGATAAACCGGAGGAAGGTGGGGACGACGTCAAGTCATCATGGCCCTTACGAG<br>TAGGGCTACACACGTGCTACAATGGCGTATACAGAGGGCGGCCAACCAGCGATGGTGAGCGA<br>ATCCCACAAAGTACGTCGTAGTCCGGATTGGAGTCTGCAACTCGACTCCATGAAGTCGGAAT

			CGCTAGTAATCGTGGATCAGAATGCCACGGTGAATACGTTCCCGGGCCTTGTACACACCGCCC<br>GTCACACCATGGGAGTGGGCT
HZOF-04	*Vibrio alfacsensis*	99.17%,1328bp	AGCGGCGGACGGGTGAGTAATGCCTAGGAAATTGCCCTGATGTGGGGGATAACCATTGGAAA<br>CGATGGCTAATACCGCATGATGCCTACGGGCCAAAGAGGGGGACCTTCGGGCCTCTCGCGTC<br>AGGATATGCCTAGGTGGGATTAGCTAGTTGGTGAGGTAAGGGCTCACCAAGGCGACGATCCC<br>TAGCTGGTCTGAGAGGATGATCAGCCACACTGGAACTGAGACACGGTCCAGACTCCTACGGG<br>AGGCAGCAGTGGGGAATATTGCACAATGGGCGCAAGCCTGATGCAGCCATGCCGCGTGTGTG<br>AAGAAGGCCTTCGGGTTGTAAAGCACTTTCAGTAGGGAGGAAGGTTCATGCGTTAATAGCGT<br>ATGGATTTGACGTTACCTACAGAAGAAGCACCGGCTAACTCCGTGCCAGCAGCCGCGGTAAT<br>ACGGAGGGTGCGAGCGTTAATCGGAATTACTGGGCGTAAAGCGCATGCAGGTGGTTTGTTAA<br>GTCAGATGTGAAAGCCCGGGGCTCAACCTCGGAATTGCATTTGAAACTGGCAGACTAGAGTA<br>CTGTAGAGGGGGGTAGAATTTCAGGTGTAGCGGTGAAATGCGTAGAGATCTGAAGGAATACC<br>GGTGGCGAAGGCGGCCCCCTGGACAGATACTGACACTCAGATGCGAAAGCGTGGGGAGCAA<br>ACAGGATTAGATACCCTGGTAGTCCACGCCGTAAACGATGTCTACTTGGAGGTTGTGGCCTTG<br>AGCCGTGGCTTTCGGAGCTAACGCGTTAAGTAGACCGCCTGGGGAGTACGGTCGCAAGATTA<br>AAACTCAAATGAATTGACGGGGGCCCGCACAAGCGGTGGAGCATGTGGTTTAATTCGATGCA<br>ACGCGAAGAACCTTACCTACTCTTGACATCCAGAGAAGCCAGYAGAGATRCAGGTGTGCCTT<br>CGGGARCTCTGAGACAGGTGCTGCATGGCTGTCGTCAGCTCGTGTTGTGAAATGTTGGGTTAA<br>GTCCCGCAACGAGCGCAACCCTTATCCTTGTTTGCCAGCGAGTAATGTCGGGAACTCCAGGG<br>AGACTGCCGGTGATAAACCGGAGGAAGGTGGGGACGACGTCAAGTCATCATGGCCCTTACGA<br>GTAGGGCTACACACGTGCTACAATGGCGCATACAGAGGGCAGCCAACTTGCGAAAGTGAGCG<br>AATCCCAAAAAGTGCGTCGTAGTCCGGATTGGAGTCTGCAACTCGACTCCATGAAGTCGGAA<br>TCGCTAGTAATCGTGGATCAGAATGCCACGGTGAATACGTTCCCGGGCCTTGTACACACCGCC<br>CGTCACACCATGGGAGTGGGCTGC
HZOF-05	*Vibrio owensii*	99.92%,1327bp	AGCGGCGGACGGGTGAGTAATGCCTAGGAAATTGCCCTGATGTGGGGGATAACCATTGGAAA<br>CGATGGCTAATACCGCATAATGCCTACGGGCCAAAGAGGGGGACCTTCGGGCCTCTCGCGTC<br>AGGATATGCCTAGGTGGGATTAGCTAGTTGGTGAGGTAATGGCTCACCAAGGCGACGATCCC<br>TAGCTGGTCTGAGAGGATGATCAGCCACACTGGAACTGAGACACGGTCCAGACTCCTACGGG<br>AGGCAGCAGTGGGGAATATTGCACAATGGGCGCAAGCCTGATGCAGCCATGCCGCGTGTGTG<br>AAGAAGGCCTTCGGGTTGTAAAGCACTTTCAGTCGTGAGGAAGGTRGTGTAGTTAATAGCTG<br>CATTRYTTGACGTTAGCGACAGAAGAAGCACCGGCTAACTCCGTGCCAGCAGCCGCGGTAAT<br>ACGGAGGGTGCGAGCGTTAATCGGAATTACTGGGCGTAAAGCGCATGCAGGTGGTTTGTTAA

			GTCAGATGTGAAAGCCCGGGGCTCAACCTCGGAATAGCATTTGAAACTGGCAGACTAGAGTA<br>CTGTAGAGGGGGGTAGAATTTCAGGTGTAGCGGTGAAATGCGTAGAGATCTGAAGGAATACC<br>GGTGGCGAAGGCGGCCCCCTGGACAGATACTGACACTCAGATGCGAAAGCGTGGGGAGCAA<br>ACAGGATTAGATACCCTGGTAGTCCACGCCGTAAACGATGTCTACTTGGAGGTTGTGGCCTTG<br>AGCCGTGGCTTTCGGAGCTAACGCGTTAAGTAGACCGCCTGGGGAGTACGGTCGCAAGATTA<br>AAACTCAAATGAATTGACGGGGGCCCGCACAAGCGGTGGAGCATGTGGTTTAATTCGATGCA<br>ACGCGAAGAACCTTACCTACTCTTGACATCCAGAGAACTTTCCAGAGATGGATTGGTGCCTTC<br>GGGAACTCTGAGACAGGTGCTGCATGGCTGTCGTCAGCTCGTGTTGTGAAATGTTGGGTTAAG<br>TCCCGCAACGAGCGCAACCCTTATCCTTGTTTGCCAGCGAGTAATGTCGGGAACTCCAGGGA<br>GACTGCCGGTGATAAACCGGAGGAAGGTGGGGACGACGTCAAGTCATCATGGCCCTTACGAG<br>TAGGGCTACACACGTGCTACAATGGCGCATACAGAGGGCAGCAAGCTAGCGATAGTGAGCG<br>AATCCCAAAAAGTGCGTCGTAGTCCGGATTGGAGTCTGCAACTCGACTCCATGAAGTCGGAA<br>TCGCTAGTAATCGTGGATCAGAATGCCACGGTGAATACGTTCCCGGGCCTTGTACACACCGCC<br>CGTCACACCATGGGAGTGGGCTG
HZOF-08	*Vibrio tubiashii*	99.32%,1324bp	LC624729
HZOF-09	*Bacillus vietnamensis*	99.86%,1387bp	LC624773
HZOF-10	*Vibrio alginolyticus*	99.93%,1343bp	AGCGGCGGACGGGTGAGTAATGCCTAGGAAATTGCCCTGATGTGGGGGATAACCATTGGAAA<br>CGATGGCTAATACCGCATAATGCCTACGGGCCAAAGAGGGGGACCTTCGGGCCTCTCGCGTC<br>AGGATATGCCTAGGTGGGATTAGCTAGTTGGTGAGGTAATGGCTCACCAAGGCGACGATCCC<br>TAGCTGGTCTGAGAGGATGATCAGCCACACTGGAACTGAGACACGGTCCAGACTCCTACGGG<br>AGGCAGCAGTGGGGAATATTGCACAATGGGCGCAAGCCTGATGCAGCCATGCCGCGTGTGTG<br>AAGAAGGCCTTCGGGTTGTAAAGCACTTTCAGTYGTGAGGAAGGTGGTRKWGTTAATAGCWS<br>YATYAYTTGACGTTAGCGACAGAAGAAGCACCGGCTAACTCCGTGCCAGCAGCCGCGGTAAT<br>ACGGAGGGTGCGAGCGTTAATCGGAATTACTGGGCGTAAAGCGCATGCAGGTGGTTTGTTAA<br>GTCAGATGTGAAAGCCCGGGGCTCAACCTCGGAATAGCATTTGAAACTGGCAGACTAGAGTA<br>CTGTAGAGGGGGGTAGAATTTCAGGTGTAGCGGTGAAATGCGTAGAGATCTGAAGGAATACC<br>GGTGGCGAAGGCGGCCCCCTGGACAGATACTGACACTCAGATGCGAAAGCGTGGGGAGCAA<br>ACAGGATTAGATACCCTGGTAGTCCACGCCGTAAACGATGTCTACTTGGAGGTTGTGGCCTTG<br>AGCCGTGGCTTTCGGAGCTAACGCGTTAAGTAGACCGCCTGGGGAGTACGGTCGCAAGATTA<br>AAACTCAAATGAATTGACGGGGGCCCGCACAAGCGGTGGAGCATGTGGTTTAATTCGATGCA<br>ACGCGAAGAACCTTACCTACTCTTGACATCCAGAGAAYTTTYCAGAGATGRATTRGTGCCTTC

			GGGAACTCTGAGACAGGTGCTGCATGGCTGTCGTCAGCTCGTGTTGTGAAATGTTGGGTTAAG<br>TCCCGCAACGAGCGCAACCCTTATCCTTGTTTGCCAGCGAGTAATGTCGGGAACTCCAGGGA<br>GACTGCCGGTGATAAACCGGAGGAAGGTGGGGACGACGTCAAGTCATCATGGCCCTTACGAG<br>TAGGGCTACACACGTGCTACAATGGCGCATACAGAGGGCAGCAAGCTAGCGATAGTGAGCG<br>AATCCCAAAAAGTGCGTCGTAGTCCGGATTGGAGTCTGCAACTCGACTCCATGAAGTCGGAA<br>TCGCTAGTAATCGTGGATCAGAATGCCACGGTGAATACGTTCCCGGGCCTTGTACACACCGCC<br>CGTCACACCATGGGAGTGGGCTGCAAAAGAAGTAGGTAG
HZOF-11	*Vibrio ponticus*	99.79%,1404bp	CAGCGACAACATTGAACCTTCGGGGGATTTGTTGGGCGGCGAGCGGCGGACGGGTGAGTAAT<br>GCCTAGGAAATTGCCCTGATGTGGGGGATAACCATTGGAAACGATGGCTAATACCGCATGAT<br>GCCTACGGGCCAAAGAGGGGGACCTTCGGGCCTCTCGCGTCAGGATATGCCTAGGTGGGATT<br>AGCTAGTTGGTGAGGTAAGGGCTCACCAAGGCGACGATCCCTAGCTGGTCTGAGAGGATGAT<br>CAGCCACACTGGAACTGAGACACGGTCCAGACTCCTACGGGAGGCAGCAGTGGGGAATATTG<br>CACAATGGGCGCAAGCCTGATGCAGCCATGCCGCGTGTATGAAGAAGGCCTTCGGGTTGTAA<br>AGTACTTTCAGTAGGGAGGAAGGTTCATRCGTTAATAGCGTATGGATTTGACGTTACCTACAG<br>AAGAAGCACCGGCTAACTCCGTGCCAGCAGCCGCGGTAATACGGAGGGTGCGAGCGTTAATC<br>GGAATTACTGGGCGTAAAGCGCATGCAGGTGGTTAGTTAAGTCAGATGTGAAAGCCCGGGGC<br>TCAACCTCGGAATTGCATTTGAAACTGGCTGACTAGAGTACTGTAGAGGGGGGTAGAATTTC<br>AGGTGTAGCGGTGAAATGCGTAGAGATCTGAAGGAATACCGGTGGCGAAGGCGGCCCCCTG<br>GACAGATACTGACACTCAGATGCGAAAGCGTGGGGAGCAAACAGGATTAGATACCCTGGTA<br>GTCCACGCCGTAAACGATGTCTACTTGGAGGTTGTGGCCTTGAGCCGTGGCTTTCGGAGCTAA<br>CGCGTTAAGTAGACCGCCTGGGGAGTACGGTCGCAAGATTAAAACTCAAATGAATTGACGGG<br>GGCCCGCACAAGCGGTGGAGCATGTGGTTTAATTCGATGCAACGCGAAGAACCTTACCTACT<br>CTTGACATCCATAGAACTTAGCAGAGATGCTTTGGTGCCTTCGGGAACTATGAGACAGGTGCT<br>GCATGGCTGTCGTCAGCTCGTGTTGTGAAATGTTGGGTTAAGTCCCGCAACGAGCGCAACCCT<br>TATCCTTGTTTGCCAGCGAGTAATGTCGGGAACTCCAGGGAGACTGCCGGTGATAAACCGGA<br>GGAAGGTGGGGACGACGTCAAGTCATCATGGCCCTTACGAGTAGGGCTACACACGTGCTACA<br>ATGGCGYATACAGAGGGCTGCCAAYCAGCGATGGTGAGCGAATCCCAAAAAGTRCGTCGTA<br>GTCCGGATTGGAGTCTGCAACTCGACTCCATGAAGTCGGAATCGCTAGTAATCGTGGATCAG<br>AATGCCACGGTGAATACGTTCCCGGGCCTTGTACACACCGCCCGTCACACCATGGGAGTGGG<br>CTGCAAAAGAAGTGGGTAGTTTAACCTTCGGGAGGACGC
HZOF-12	*Vibrio alfacsensis*	98.97%,1349bp	AGCGGCGGACGGGTGAGTAATGCCTAGGAAATTGCCCTGATGTGGGGGATAACCATTGGAAA<br>CGATGGCTAATACCGCATGATGCCTACGGGCCAAAGAGGGGGACCTTCGGGCCTCTCGCGTC

			AGGATATGCCTAGGTGGGATTAGCTAGTTGGTGAGGTAAGGGCTCACCAAGGCGACGATCCC<br>TAGCTGGTCTGAGAGGATGATCAGCCACACTGGAACTGAGACACGGTCCAGACTCCTACGGG<br>AGGCAGCAGTGGGGAATATTGCACAATGGGCGCAAGCCTGATGCAGCCATGCCGCGTGTGTG<br>AAGAAGGCCTTCGGGTTGTAAAGCACTTTCAGTAGGGAGGAAGGTTCATGCGTTAATAGCGT<br>ATGGATTTGACGTTACCTACAGAAGAAGCACCGGCTAACTCCGTGCCAGCAGCCGCGGTAAT<br>ACGGAGGGTGCGAGCGTTAATCGGAATTACTGGGCGTAAAGCGCATGCAGGTGGTTTGTTAA<br>GTCAGATGTGAAAGCCCGGGGCTCAACCTCGGAATTGCATTTGAAACTGGCAGACTAGAGTA<br>CTGTAGAGGGGGGTAGAATTTCAGGTGTAGCGGTGAAATGCGTAGAGATCTGAAGGAATACC<br>GGTGGCGAAGGCGGCCCCCTGGACAGATACTGACACTCAGATGCGAAAGCGTGGGGAGCAA<br>ACAGGATTAGATACCCTGGTAGTCCACGCCGTAAACGATGTCTACTTGGAGGTTGTGGCCTTG<br>AGCCGTGGCTTTCGGAGCTAACGCGTTAAGTAGACCGCCTGGGGAGTACGGTCGCAAGATTA<br>AAACTCAAATGAATTGACGGGGGCCCGCACAAGCGGTGGAGCATGTGGTTTAATTCGATGCA<br>ACGCGAAGAACCTTACCTACTCTTGACATCCAGAGAAGCCAGYAGAGATRCAGGTGTGCCTT<br>CGGGAACTCTGAGACAGGTGCTGCATGGCTGTCGTCAGCTCGTGTTGTGAAATGTTGGGTTAA<br>GTCCCGCAACGAGCGCAACCCTTATCCTTGTTTGCCAGCGAGTAATGTCGGGAACTCCAGGG<br>AGACTGCCGGTGATAAACCGGAGGAAGGTGGGGACGACGTCAAGTCATCATGGCCCTTACGA<br>GTAGGGCTACACACGTGCTACAATGGCGCATACAGAGGGCAGCCAACTTGCGAAAGTGAGCG<br>AATCCCAAAAAGTGCGTCGTAGTCCGGATTGGAGTCTGCAACTCGACTCCATGAAGTCGGAA<br>TCGCTAGTAATCGTGGATCAGAATGCCACGGTGAATACGTTCCCGGGCCTTGTACACACCGCC<br>CGTCACACCATGGGAGTGGGCTGCAAAAGAAGTAGGTAGTTTAAC
HZOF-13	*Vibrio ponticus*	99.78%,1364bp	AGCGGCGGACGGGTGAGTAATGCCTAGGAAATTGCCCTGATGTGGGGGATAACCATTGGAAA<br>CGATGGCTAATACCGCATGATGCCTACGGGCCAAAGAGGGGGACCTTCGGGCCTCTCGCGTC<br>AGGATATGCCTAGGTGGGATTAGCTAGTTGGTGAGGTAAGGGCTCACCAAGGCGACGATCCC<br>TAGCTGGTCTGAGAGGATGATCAGCCACACTGGAACTGAGACACGGTCCAGACTCCTACGGG<br>AGGCAGCAGTGGGGAATATTGCACAATGGGCGCAAGCCTGATGCAGCCATGCCGCGTGTATG<br>AAGAAGGCCTTCGGGTTGTAAAGTACTTTCAGTAGGGAGGAAGGTTCATRCGTTAATAGCGT<br>ATGGATTTGACGTTACCTACAGAAGAAGCACCGGCTAACTCCGTGCCAGCAGCCGCGGTAAT<br>ACGGAGGGTGCGAGCGTTAATCGGAATTACTGGGCGTAAAGCGCATGCAGGTGGTTAGTTAA<br>GTCAGATGTGAAAGCCCGGGGCTCAACCTCGGAATTGCATTTGAAACTGGCTGACTAGAGTA<br>CTGTAGAGGGGGGTAGAATTTCAGGTGTAGCGGTGAAATGCGTAGAGATCTGAAGGAATACC<br>GGTGGCGAAGGCGGCCCCCTGGACAGATACTGACACTCAGATGCGAAAGCGTGGGGAGCAA<br>ACAGGATTAGATACCCTGGTAGTCCACGCCGTAAACGATGTCTACTTGGAGGTTGTGGCCTTG

			AGCCGTGGCTTTCGGAGCTAACGCGTTAAGTAGACCGCCTGGGGAGTACGGTCGCAAGATTA<br>AAACTCAAATGAATTGACGGGGGCCCGCACAAGCGGTGGAGCATGTGGTTTAATTCGATGCA<br>ACGCGAAGAACCTTACCTACTCTTGACATCCATAGAACTTAGCAGAGATGCTTTGGTGCCTTC<br>GGGAACTATGAGACAGGTGCTGCATGGCTGTCGTCAGCTCGTGTTGTGAAATGTTGGGTTAA<br>GTCCCGCAACGAGCGCAACCCTTATCCTTGTTTGCCAGCGAGTAATGTCGGGAACTCCAGGG<br>AGACTGCCGGTGATAAACCGGAGGAAGGTGGGGACGACGTCAAGTCATCATGGCCCTTACGA<br>GTAGGGCTACACACGTGCTACAATGGCGYATACAGAGGGCTGCCAAYCAGCGATGGTGAGC<br>GAATCCCAAAAAGTRCGTCGTAGTCCGGATTGGAGTCTGCAACTCGACTCCATGAAGTCGGA<br>ATCGCTAGTAATCGTGGATCAGAATGCCACGGTGAATACGTTCCCGGGCCTTGTACACACCGC<br>CCGTCACACCATGGGAGTGGGCTGCAAAAGAAGTGGGTAGTTTAACCTTCGGGAGGACGCT
HZOF-14	*Vibrio alginolyticus*	99.85%,1364bp	AGCGGCGGACGGGTGAGTAATGCCTAGGAAATTGCCCTGATGTGGGGGATAACCATTGGAAA<br>CGATGGCTAATACCGCATAATGCCTACGGGCCAAAGAGGGGGACCTTCGGGCCTCTCGCGTC<br>AGGATATGCCTAGGTGGGATTAGCTAGTTGGTGAGGTAATGGCTCACCAAGGCGACGATCCC<br>TAGCTGGTCTGAGAGGATGATCAGCCACACTGGAACTGAGACACGGTCCAGACTCCTACGGG<br>AGGCAGCAGTGGGGAATATTGCACAATGGGCGCAAGCCTGATGCAGCCATGCCGCGTGTGTG<br>AAGAAGGCCTTCGGGTTGTAAAGCACTTTCAGTCGTGAGGAAGGTRGTGTAGTTAATAGCTG<br>CATTAYTTGACGTTAGCGACAGAAGAAGCACCGGCTAACTCCGTGCCAGCAGCCGCGGTAAT<br>ACGGAGGGTGCGAGCGTTAATCGGAATTACTGGGCGTAAAGCGCATGCAGGTGGTTTGTTAA<br>GTCAGATGTGAAAGCCCGGGGCTCAACCTCGGAATAGCATTTGAAACTGGCAGACTAGAGTA<br>CTGTAGAGGGGGGTAGAATTTCAGGTGTAGCGGTGAAATGCGTAGAGATCTGAAGGAATACC<br>GGTGGCGAAGGCGGCCCCCTGGACAGATACTGACACTCAGATGCGAAAGCGTGGGGAGCAA<br>ACAGGATTAGATACCCTGGTAGTCCACGCCGTAAACGATGTCTACTTGGAGGTTGTGGCCTTG<br>AGCCGTGGCTTTCGGAGCTAACGCGTTAAGTAGACCGCCTGGGGAGTACGGTCGCAAGATTA<br>AAACTCAAATGAATTGACGGGGGCCCGCACAAGCGGTGGAGCATGTGGTTTAATTCGATGCA<br>ACGCGAAGAACCTTACCTACTCTTGACATCCAGAGAACTTTYCAGAGATGRATTGGTGCCTTC<br>GGGAACTCTGAGACAGGTGCTGCATGGCTGTCGTCAGCTCGTGTTGTGAAATGTTGGGTTAAG<br>TCCCGCAACGAGCGCAACCCTTATCCTTGTTTGCCAGCGAGTAATGTCGGGAACTCCAGGGA<br>GACTGCCGGTGATAAACCGGAGGAAGGTGGGGACGACGTCAAGTCATCATGGCCCTTACGAG<br>TAGGGCTACACACGTGCTACAATGGCGCATACAGAGGGCAGCAAGCTAGCGATAGTGAGCG<br>AATCCCAAAAAGTGCGTCGTAGTCCGGATTGGAGTCTGCAACTCGACTCCATGAAGTCGGAA<br>TCGCTAGTAATCGTGGATCAGAATGCCACGGTGAATACGTTCCCGGGCCTTGTACACACCGCC<br>CGTGCACACCATGGGAGTGGGCTGCAAAAGAAGTAGGTAGTTTAACCTTCGGGAGGACGC

HLBS-01	*Vibrio alginolyticus*	98.23%,1308bp	AGCGGCGGACGGGTGAGTAATGCCTAGGAAATTGCCCTGATGTGGGGGATAACCATTGGAAA<br>CGATGGCTAATACCGCATGATGCCTACGGGCYAAAGAGGGGGACCTTCGGGCCTCTCGCGTC<br>AGGATATGCCTAGGTGGGATTAGCTAGTTGGTGAGGTAAGGGCTCACCAAGGCRACGATCCC<br>TAGCTGGTCTGAGAGGATGATCAGCCACACTGGAACTGAGACACGGTCCAGACTCCTACGGG<br>AGGCAGCAGTGGGGAATATTGCACAATGGGCGCAAGCCTGATGCAGCCATGCCGCGTGTRTG<br>AAGAAGGCCTTCGGGTTGTAAAGYACTTTCAGTCGTGAGGAAGGYRGTGTMGTTAATAGCTG<br>CATYRTTTGACGTTAGCGACAGAAGAAGCACCGGCTAACTCCGTGCCAGCAGCCGCGGTAAT<br>ACGGAGGGTGCGAGCGTTAATCGGAATTACTGGGCGTAAAGCGCATGCAGGTGGTTTGTTAA<br>GTCAGATGTGAAAGCCCGGGGCTCAACCTCGGAATAGCATTTGAAACTGGCAGACTAGAGTA<br>CTGTAGAGGGGGGTAGAATTTCAGGTGTAGCGGTGAAATGCGTAGAGATCTGAAGGAATACC<br>GGTGGCGAAGGCGGCCCCCTGGACAGATACTGACACTCAGATGCGAAAGCGTGGGGAGCAA<br>ACAGGATTAGATACCCTGGTAGTCCACGCCGTAAACGATGTCTACTTGGAGGTTGTGGCCTTG<br>AGCCGTGGCTTTCGGAGCTAACGCGTTAAGTAGACCGCCTGGGGAGTACGGTCGCAAGATTA<br>AAACTCAAATGAATTGACGGGGGCCCGCACAAGCGGTGGAGCATGTGGTTTAATTCGATGCA<br>ACGCGAAGAACCTTACCTACTCTTGACATCCAGAGAACTTTCCAGAGATGGATTGGTGCCTTC<br>GGGAACTCTGAGACAGGTGCTGCATGGCTGTCGTCAGCTCGTGTTGTGAAATGTTGGGTTAAG<br>TCCCGCAACGAGCGCAACCCTTATCCTTGTTTGCCAGCGAGTAATGTCGGGAACTCCAGGGA<br>GACTGCCGGTGATAAACCGGAGGAAGGTGGGGACGACGTCAAGTCATCATGGCCCTTACGAG<br>TAGGGCTACACACGTGCTACAATGGCGCATACAGAGGGCGGCCAACTTGCGAAAGTGAGCGA<br>ATCCCAAAAAGTGCGTCGTAGTCCGGATTGGAGTCTGCAACTCGACTCCATGAAGTCGGAAT<br>CGCTAGTAATCGTGGATCAGAATGCCACGGTGAATACGTTCCCGGGCCTTGTACACACCGCCC<br>GTC
HLBS-02	*Vibrio alginolyticus*	100%,1363bp	AGCGGCGGACGGGTGAGTAATGCCTAGGAAATTGCCCTGATGTGGGGGATAACCATTGGAAA<br>CGATGGCTAATACCGCATRATGCCTACGGGCCAAAGAGGGGGACCTTCGGGCCTCTCGCGTC<br>AGGATATGCCTAGGTGGGATTAGCTAGTTGGTGAGGTAAGGGCTCACCAAGGCGACGATCCC<br>TAGCTGGTCTGAGAGGATGATCAGCCACACTGGAACTGAGACACGGTCCAGACTCCTACGGG<br>AGGCAGCAGTGGGGAATATTGCACAATGGGCGCAAGCCTGATGCAGCCATGCCGCGTGTRTG<br>AAGAAGGCCTTCGGGTTGTAAAGYACTTTCAGTCGTGAGGAAGGTRGTGTAGTTAATAGCTG<br>CATTATTTGACGTTAGCGACAGAAGAAGCACCGGCTAACTCCGTGCCAGCAGCCGCGGTAAT<br>ACGGAGGGTGCGAGCGTTAATCGGAATTACTGGGCGTAAAGCGCATGCAGGTGGTTTGTTAA<br>GTCAGATGTGAAAGCCCGGGGCTCAACCTCGGAATAGCATTTGAAACTGGCAGACTAGAGTA<br>CTGTAGAGGGGGGTAGAATTTCAGGTGTAGCGGTGAAATGCGTAGAGATCTGAAGGAATACC

			GGTGGCGAAGGCGGCCCCCTGGACAGATACTGACACTCAGATGCGAAAGCGTGGGGAGCAA<br>ACAGGATTAGATACCCTGGTAGTCCACGCCGTAAACGATGTCTACTTGGAGGTTGTGGCCTTG<br>AGCCGTGGCTTTCGGAGCTAACGCGTTAAGTAGACCGCCTGGGGAGTACGGTCGCAAGATTA<br>AAACTCAAATGAATTGACGGGGGCCCGCACAAGCGGTGGAGCATGTGGTTTAATTCGATGCA<br>ACGCGAAGAACCTTACCTACTCTTGACATCCAGAGAACTTTCCAGAGATGGATTGGTGCCTTC<br>GGGAACTCTGAGACAGGTGCTGCATGGCTGTCGTCAGCTCGTGTTGTGAAATGTTGGGTTAAG<br>TCCCGCAACGAGCGCAACCCTTATCCTTGTTTGCCAGCGAGTAATGTCGGGAACTCCAGGGA<br>GACTGCCGGTGATAAACCGGAGGAAGGTGGGGACGACGTCAAGTCATCATGGCCCTTACGAG<br>TAGGGCTACACACGTGCTACAATGGCGCATACAGAGGGCGGCCAACTTGCGAAAGTGAGCGA<br>ATCCCAAAAAGTGCGTCGTAGTCCGGATTGGAGTCTGCAACTCGACTCCATGAAGTCGGAAT<br>CGCTAGTAATCGTGGATCAGAATGCCACGGTGAATACGTTCCCGGGCCTTGTACACACCGCCC<br>GTCACACCATGGGAGTGGGCTGCAAAAGAAGTAGGTAGTTTAACCTTCGGGGGGACGC
HLBS-03	*Vibrio alginolyticus*	99.55%,1355bp	AGCGGCGGACGGGTGAGTAATGCCTAGGAAATTGCCCTGATGTGGGGGATAACCATTGGAAA<br>CGATGGCTAATACCGCATRATGCCTACGGGCCAAAGAGGGGGACCTTCGGGCCTCTCGCGTC<br>AGGATATGCCTAGGTGGGATTAGCTAGTTGGTGAGGTAAGGGCTCACCAAGGCGACGATCCC<br>TAGCTGGTCTGAGAGGATGATCAGCCACACTGGAACTGAGACACGGTCCAGACTCCTACGGG<br>AGGCAGCAGTGGGGAATATTGCACAATGGGCGCAAGCCTGATGCAGCCATGCCGCGTGTRTG<br>AAGAAGGCCTTCGGGTTGTAAAGYACTTTCAGTCGTGAGGAAGGTRGTGTAGTTAATAGCTG<br>CATTATTTGACGTTAGCGACAGAAGAAGCACCGGCTAACTCCGTGCCAGCAGCCGCGGTAAT<br>ACGGAGGGTGCGAGCGTTAATCGGAATTACTGGGCGTAAAGCGCATGCAGGTGGTTTGTTAA<br>GTCAGATGTGAAAGCCCGGGGCTCAACCTCGGAATAGCATTTGAAACTGGCAGACTAGAGTA<br>CTGTAGAGGGGGGTAGAATTTCAGGTGTAGCGGTGAAATGCGTAGAGATCTGAAGGAATACC<br>GGTGGCGAAGGCGGCCCCCTGGACAGATACTGACACTCAGATGCGAAAGCGTGGGGAGCAA<br>ACAGGATTAGATACCCTGGTAGTCCACGCCGTAAACGATGTCTACTTGGAGGTTGTGGCCTTG<br>AGCCGTGGCTTTCGGAGCTAACGCGTTAAGTAGACCGCCTGGGGAGTACGGTCGCAAGATTA<br>AAACTCAAATGAATTGACGGGGGCCCGCACAAGCGGTGGAGCATGTGGTTTAATTCGATGCA<br>ACGCGAAGAACCTTACCTACTCTTGACATCCAGAGAACTTTCCAGAGATGGATTGGTGCCTTC<br>GGGAACTCTGAGACAGGTGCTGCATGGCTGTCGTCAGCTCGTGTTGTGAAATGTTGGGTTAAG<br>TCCCGCAACGAGCGCAACCCTTATCCTTGTTTGCCAGCGAGTAATGTCGGGAACTCCAGGGA<br>GACTGCCGGTGATAAACCGGAGGAAGGTGGGGACGACGTCAAGTCATCATGGCCCTTACGAG<br>TAGGGCTACACACGTGCTACAATGGCGCATACAGAGGGCGGCCAACTTGCGAAAGTGAGCGA<br>ATCCCAAAAAGTGCGTCGTAGTCCGGATTGGAGTCTGCAACTCGACTCCATGAAGTCGGAAT

			CGCTAGTAATCGTGGATCAGAATGCCACGGTGAATACGTTCCCGGGCCTTGTACACACCGCCC<br>GTCACACCATGGGAGTGGGCTGCAAAAGAAGTAGGTAGTTTAACCTTCGG
HLBS-04	*Vibrio alginolyticus*	99.86%,1411bp	AGCGGCGGACGGGTGAGTAATGCCTAGGAAATTGCCCTGATGTGGGGGATAACCATTGGAAA<br>CGATGGCTAATACCGCATAATGCCTACGGGCCAAAGAGGGGGACCTTCGGGCCTCTCGCGTC<br>AGGATATGCCTAGGTGGGATTAGCTAGTTGGTGAGGTAATGGCTCACCAAGGCGACGATCCC<br>TAGCTGGTCTGAGAGGATGATCAGCCACACTGGAACTGAGACACGGTCCAGACTCCTACGGG<br>AGGCAGCAGTGGGGAATATTGCACAATGGGCGCAAGCCTGATGCAGCCATGCCGCGTGTGTG<br>AAGAAGGCCTTCGGGTTGTAAAGCACTTTCAGTCGTGAGGAAGGTRGTRKWGTTAATAGCWS<br>YATYAYTTGACGTTAGCGACAGAAGAAGCACCGGCTAACTCCGTGCCAGCAGCCGCGGTAAT<br>ACGGAGGGTGCGAGCGTTAATCGGAATTACTGGGCGTAAAGCGCATGCAGGTGGTTTGTTAA<br>GTCAGATGTGAAAGCCCGGGGCTCAACCTCGGAATAGCATTTGAAACTGGCAGACTAGAGTA<br>CTGTAGAGGGGGGTAGAATTTCAGGTGTAGCGGTGAAATGCGTAGAGATCTGAAGGAATACC<br>GGTGGCGAAGGCGGCCCCCTGGACAGATACTGACACTCAGATGCGAAAGCGTGGGGAGCAA<br>ACAGGATTAGATACCCTGGTAGTCCACGCCGTAAACGATGTCTACTTGGAGGTTGTGGCCTTG<br>AGCCGTGGCTTTCGGAGCTAACGCGTTAAGTAGACCGCCTGGGGAGTACGGTCGCAAGATTA<br>AAACTCAAATGAATTGACGGGGGCCCGCACAAGCGGTGGAGCATGTGGTTTAATTCGATGCA<br>ACGCGAAGAACCTTACCTACTCTTGACATCCAGAGTAACTTTCCAGAGATGGATTGGTGCCTT<br>CGGGAACTCTGAGACAGGTGCTGCATGGCTGTCGTCAGCTCGTGTTGTGAAATGTTGGGTTAA<br>GTCCCGCAACGAGCGCAACCCTTATCCTTGTTTGCCAGCGAGTAATGTCGGGAACTCCAGGG<br>AGACTGCCGGTGATAAACCGGAGGAAGGTGGGGACGACGTCAAGTCATCATGGCCCTTACGA<br>GTAGGGCTACACACGTGCTACAATGGCGCATACAGAGGGCAGCAAGCTAGCGATAGTGAGC<br>GAATCCCAAAAAGTGCGTCGTAGTCCGGATTGGAGTCTGCAACTCGACTCCATGAAGTCGGA<br>ATCGCTAGTAATCGTGGATCAGAATGCCACGGTGAATACGTTCCCGGGCCTTGTACACACCGC<br>CCGTCACACCATGGGAGTGGGCTGCAAAAGAAGTAGGTAGTTTAACCTTCGGGAGGACGCTT<br>ACCACTTTGTGGTTCATGACTGGGGTGAAGTCGTAACAAGGTAAC
HLBS-05	*Photobacterium damselae*	99.65%,1410bp	LC624730
HLBS-07	*Vibrio alginolyticus*	100%,1363bp	Genome analysis<br>AP023187 AP023188
HLBS-08	*Vibrio alginolyticus*	99.93%,1363bp	AGCGGCGGACGGGTGAGTAATGCCTAGGAAATTGCCCTGATGTGGGGGATAACCATTGGAAA<br>CGATGGCTAATACCGCATRATGCCTACGGGCCAAAGAGGGGGACCTTCGGGCCTCTCGCGTC

			AGGATATGCCTAGGTGGGATTAGCTAGTTGGTGAGGTAAGGGCTCACCAAGGCGACGATCCC<br>TAGCTGGTCTGAGAGGATGATCAGCCACACTGGAACTGAGACACGGTCCAGACTCCTACGGG<br>AGGCAGCAGTGGGGAATATTGCACAATGGGCGCAAGCCTGATGCAGCCATGCCGCGTGTRTG<br>AAGAAGGCCTTCGGGTTGTAAAGYACTTTCAGTCGTGAGGAAGGTRGTGTAGTTAATAGCTG<br>CATTATTTGACGTTAGCGACAGAAGAAGCACCGGCTAACTCCGTGCCAGCAGCCGCGGTAAT<br>ACGGAGGGTGCGAGCGTTAATCGGAATTACTGGGCGTAAAGCGCATGCAGGTGGTTTGTTAA<br>GTCAGATGTGAAAGCCCGGGGCTCAACCTCGGAATAGCATTTGAAACTGGCAGACTAGAGTA<br>CTGTAGAGGGGGGTAGAATTTCAGGTGTAGCGGTGAAATGCGTAGAGATCTGAAGGAATACC<br>GGTGGCGAAGGCGGCCCCCTGGACAGATACTGACACTCAGATGCGAAAGCGTGGGGAGCAA<br>ACAGGATTAGATACCCTGGTAGTCCACGCCGTAAACGATGTCTACTTGGAGGTTGTGGCCTTG<br>AGCCGTGGCTTTCGGAGCTAACGCGTTAAGTAGACCGCCTGGGGAGTACGGTCGCAAGATTA<br>AAACTCAAATGAATTGACGGGGGCCCGCACAAGCGGTGGAGCATGTGGTTTAATTCGATGCA<br>ACGCGAAGAACCTTACCTACTCTTGACATCCAGAGAACTTTCCAGAGATGGATTGGTGCCTTC<br>GGGAACTCTGAGACAGGTGCTGCATGGCTGTCGTCAGCTCGTGTTGTGAAATGTTGGGTTAAG<br>TCCCGCAACGAGCGCAACCCTTATCCTTGTTTGCCAGCGAGTAATGTCGGGAACTCCAGGGA<br>GACTGCCGGTGATAAACCGGAGGAAGGTGGGGACGACGTCAAGTCATCATGGCCCTTACGAG<br>TAGGGCTACACACGTGCTACAATGGCGCATACAGAGGGCRGCCAACTTGCGARAGTGAGCGA<br>ATCCCAAAAAGTGCGTCGTAGTCCGGATTGGAGTCTGCAACTCGACTCCATGAAGTCGGAAT<br>CGCTAGTAATCGTGGATCAGAATGCCACGGTGAATACGTTCCCGGGCCTTGTACACACCGCCC<br>GTCACACCATGGGAGTGGGCTGCAAAAGAAGTAGGTAGTTTAACCTTCGGGGGGACGC
HZOS-01	*Vibrio alginolyticus*	99.71% 1367bp	AGCGGCGGACGGGTGAGTAATGCCTAGGAAATTGCCCTGATGTGGGGGATAACCATTGGAAA<br>CGATGGCTAATACCGCATGATGCCTACGGGCCAAAGAGGGGGACCTTCGGGCCTCTCGCGTC<br>AGGATATGCCTAGGTGGGATTAGCTAGTTGGTGAGGTAAGGGCTCACCAAGGCGACGATCCC<br>TAGCTGGTCTGAGAGGATGATCAGCCACACTGGAACTGAGACACGGTCCAGACTCCTACGGG<br>AGGCAGCAGTGGGGAATATTGCACAATGGGCGCAAGCCTGATGCAGCCATGCCGCGTGTGTG<br>AAGAAGGCCTTCGGGTTGTAAAGCACTTTCAGTCGTGAGGAAGGTRGTRWMGTTAATAGCNN<br>NWTYRYTTGACGTTAGCGACAGAAGAAGCACCGGCTAACTCCGTGCCAGCAGCCGCGGTAAT<br>ACGGAGGGTGCGAGCGTTAATCGGAATTACTGGGCGTAAAGCGCATGCAGGTGGTTTGTTAA<br>GTCAGATGTGAAAGCCCGGGGCTCAACCTCGGAATWGCATTTGAAACTGGCAGACTAGAGTA<br>CTGTAGAGGGGGGTAGAATTTCAGGTGTAGCGGTGAAATGCGTAGAGATCTGAAGGAATACC<br>GGTGGCGAAGGCGGCCCCCTGGACAGATACTGACACTCAGATGCGAAAGCGTGGGGAGCAA<br>ACAGGATTAGATACCCTGGTAGTCCACGCCGTAAACGATGTCTACTTGGAGGTTGTGGCCTTG

			AGCCGTGGCTTTCGGAGCTAACGCGTTAAGTAGACCGCCTGGGGAGTACGGTCGCAAGATTA<br>AAACTCAAATGAATTGACGGGGGCCCGCACAAGCGGTGGAGCATGTGGTTTAATTCGATGCA<br>ACGCGAAGAACCTTACCTACTCTTGACATCCAGAGAACTTTCCAGAGATGGATTGGTGCCTTC<br>GGGAACTCTGAGACAGGTGCTGCATGGCTGTCGTCAGCTCGTGTTGTGAAATGTTGGGTTAAG<br>TCCCGCAACGAGCGCAACCCTTATCCTTGTTTGCCAGCGAGTAATGTCGGGAACTCCAGGGA<br>GACTGCCGGTGATAAACCGGAGGAAGGTGGGGACGACGTCAAGTCATCATGGCCCTTACGAG<br>TAGGGCTACACACGTGCTACAATGGCGCATACAGAGGGCGGCCAACTTGCGAAAGTGAGCGA<br>ATCCCAAAAAGTGCGTCGTAGTCCGGATTGGAGTCTGCAACTCGACTCCATGAAGTCGGAAT<br>CGCTAGTAATCGTGGATCAGAATGCCACGGTGAATACGTTCCCGGGCCTTGTACACCCCGCCC<br>GTCACACCATGGGAGTGGGCTGCAAAAGAAGTAGGTAGTTTAACCTTCGGGGGGACGCTACC
HZOS-02	*Photobacterium lutimaris*	99.04% 1361bp	AGCGGCGGACGGGTGAGTAATGCCTGGGAATATGCCCTGATGTGGGGGATAACCATTGGAAA<br>CGATGGCTAATACCGCATAATCTCTTCGGAGCAAAGAGGGGGACCTTCGGGCCTCTCGCGTC<br>AGGATTAGCCCAGGTGAGATTAGCTTGTTGGTGAGGTAAGAGCTCACCAAGGCAACGATCTC<br>TAGCTGGTCTGAGAGGATGATCAGCCACACTGGAACTGAGACACGGTCCAGACTCCTACGGG<br>AGGCAGCAGTGGGGAATATTGCACAATGGGGGAAACCCTGATGCAGCCATGCCGCGTGTATG<br>AAGAAGGCCTTCGGGTTGTAAAGTACTTTCAGTTGTGAGGAAGGCGTATRCGTTAATAGCGT<br>ATGTGTTTGACGTTAGCAACAGAAGAAGCACCGGCTAACTCCGTGCCAGCAGCCGCGGTAAT<br>ACGGAGGGTGCGAGCGTTAATCGGAATTACTGGGCGTAAAGCGCATGCAGGCGGCTTGTTAA<br>GCCAGATGTGAAAGCCCGGGGCTCAACCTCGGAATAGCATTTGGAACTGGCAGGCTAGAGTC<br>TTGTAGAGGGGGGTAGAATTTCAGGTGTAGCGGTGAAATGCGTAGAGATCTGAAGGAATACC<br>GGTGGCGAAGGCGGCCCCCTGGACAAAGACTGACGCTCAGATGCGAAAGCGTGGGGAGCAA<br>ACAGGATTAGATACCCTGGTAGTCCACGCCGTAAACGATGTCTACTTGGAGGCTGGAACCTT<br>GAGTTCTGGCTTTCGGAGCTAACGCGTTAAGTAGACCGCCTGGGGAGTACGGTCGCAAGATT<br>AAAACTCAAATGAATTGACGGGGGCCCGCACAAGCGGTGGAGCATGTGGTTTAATTCGATGC<br>AACGCGAAGAACCTTACCTACTCTTGACATCCAGAGAACTTTCCAGAGATGGATTGGTGCCTT<br>CGGGAACTCTGAGACAGGTGCTGCATGGCTGTCGTCAGCTCGTGTTGTGAAATGTTGGGTTAA<br>GTCCCGCAACGAGCGCAACCCCTATCCTTGTTTGCCAGCRCGTAATGGYGGGAACTCCAGGG<br>AGACTGCCGGTGATAAACCGGAGGAAGGTGGGGACGACGTCAAGTCATCATGGCCCTTACGA<br>GTAGGGCTACACACGTGCTACAATGGCATATACAGAGGGCTGCMARCYAGCGATRGTGAGC<br>GAATCCCACAAAGTATGTCGTAGTCCGGATTGGAGTCTGCAACTCGACTCCATGAAGTCGGA<br>ATCGCTAGTAATCGTAGATCAGAATGCTACGGTGAATACGTTCCCGGGCCTTGTACACACCGC<br>CCTCCACACCATGGGAGTGGGCTGCACCAGAAGTAGATAGCTTAACCTTCGGGGGGGC

HZOS-03	*Vibrio campbellii*	99.85% 1358bp	AGCGGCGGACGGGTGAGTAATGCCTAGGAAATTGCCCTGATGTGGGGGATAACCATTGGAAA<br>CGATGGCTAATACCGCATAAYRCCTWYGGGYCAAAGAGGGGGACCTTCGGGCCTCTCGCGTC<br>AGGATATGCCTAGGTGGGATTAGCTAGTTGGTGAGGTAATGGCTCACCAAGGCGACGATCCC<br>TAGCTGGTCTGAGAGGATGATCAGCCACACTGGAACTGAGACACGGTCCAGACTCCTACGGG<br>AGGCAGCAGTGGGGAATATTGCACAATGGGCGCAAGCCTGATGCAGCCATGCCGCGTGTGTG<br>AAGAAGGCCTTCGGGTTGTAAAGCACTTTCAGTCGTGAGGAAGGTRGTGTAGTTAATAGCTG<br>CATTAYTTGACGTTAGCGACAGAAGAAGCACCGGCTAACTCCGTGCCAGCAGCCGCGGTAAT<br>ACGGAGGGTGCGAGCGTTAATCGGAATTACTGGGCGTAAAGCGCATGCAGGTGGTTTGTTAA<br>GTCAGATGTGAAAGCCCGGGGCTCAACCTCGGAATTGCATTTGAAACTGGCAGACTAGAGTA<br>CTGTAGAGGGGGGTAGAATTTCAGGTGTAGCGGTGAAATGCGTAGAGATCTGAAGGAATACC<br>GGTGGCGAAGGCGGCCCCCTGGACAGATACTGACACTCAGATGCGAAAGCGTGGGGAGCAA<br>ACAGGATTAGATACCCTGGTAGTCCACGCCGTAAACGATGTCTACTTGGAGGTTGTGGCCTTG<br>AGCCGTGGCTTTCGGAGCTAACGCGTTAAGTAGACCGCCTGGGGAGTACGGTCGCAAGATTA<br>AAACTCAAATGAATTGACGGGGGCCCGCACAAGCGGTGGAGCATGTGGTTTAATTCGATGCA<br>ACGCGAAGAACCTTACCTACTCTTGACATCCAGAGAACTTTTCAGAGATGAATTGGTGCCTTC<br>GGGAACTCTGAGACAGGTGCTGCATGGCTGTCGTCAGCTCGTGTTGTGAAATGTTGGGTTAAG<br>TCCCGCAACGAGCGCAACCCTTATCCTTGTTTGCCAGCGAGTAATGTCGGGAACTCCAGGGA<br>GACTGCCGGTGATAAACCGGAGGAAGGTGGGGACGACGTCAAGTCATCATGGCCCTTACGAG<br>TAGGGCTACACACGTGCTACAATGGCGCATACAGAGGGCRGCCAACTTGCGAGAGTGAGCGA<br>ATCCCAAAAAGTGCGTCGTAGTCCGGATCGGAGTCTGCAACTCGACTCCGTGAAGTCGGAAT<br>CGCTAGTAATCGTGGATCAGAATGCCACGGTGAATACGTTCCCGGGCCTTGTACACACCGCCC<br>GTCACACCATGGGAGTGGGCTGCAAAAGAAGTAGGTAGTTTAACCTTCGGGAG
HZOS-04	*Vibrio alginolyticus*	99.78% 1368bp	AGCGGCGGACGGGTGAGTAATGCCTAGGAAATTGCCCTGATGTGGGGGATAACCATTGGAAA<br>CGATGGCTAATACCGCATGATGCCTACGGGCCAAAGAGGGGGACCTTCGGGCCTCTCGCGTC<br>AGGATATGCCTAGGTGGGATTAGCTAGTTGGTGAGGTAAGGGCTCACCAAGGCGACGATCCC<br>TAGCTGGTCTGAGAGGATGATCAGCCACACTGGAACTGAGACACGGTCCAGACTCCTACGGG<br>AGGCAGCAGTGGGGAATATTGCACAATGGGCGCAAGCCTGATGCAGCCATGCCGCGTGTGTG<br>AAGAAGGCCTTCGGGTTGTAAAGCACTTTCAGTCGTGAGGAAGGYRGTNNNGTTAATAGCNN<br>NNTYRYTTGACGTTAGCGACAGAAGAAGCACCGGCTAACTCCGTGCCAGCAGCCGCGGTAAT<br>ACGGAGGGTGCGAGCGTTAATCGGAATTACTGGGCGTAAAGCGCATGCAGGTGGTTTGTTAA<br>GTCAGATGTGAAAGCCCGGGGCTCAACCTCGGAATAGCATTTGAAACTGGCAGACTAGAGTA<br>CTGTAGAGGGGGGTAGAATTTCAGGTGTAGCGGTGAAATGCGTAGAGATCTGAAGGAATACC

			GGTGGCGAAGGCGGCCCCCTGGACAGATACTGACACTCAGATGCGAAAGCGTGGGGAGCAA<br>ACAGGATTAGATACCCTGGTAGTCCACGCCGTAAACGATGTCTACTTGGAGGTTGTGGCCTTG<br>AGCCGTGGCTTTCGGAGCTAACGCGTTAAGTAGACCGCCTGGGGAGTACGGTCGCAAGATTA<br>AAACTCAAATGAATTGACGGGGGCCCGCACAAGCGGTGGAGCATGTGGTTTAATTCGATGCA<br>ACGCGAAGAACCTTACCTACTCTTGACATCCAGAGAACTTTCCAGAGATGGATTGGTGCCTTC<br>GGGAACTCTGAGACAGGTGCTGCATGGCTGTCGTCAGCTCGTGTTGTGAAATGTTGGGTTAAG<br>TCCCGCAACGAGCGCAACCCTTATCCTTGTTTGCCAGCGAGTAATGTCGGGAACTCCAGGGA<br>GACTGCCGGTGATAAACCGGAGGAAGGTGGGGACGACGTCAAGTCATCATGGCCCTTACGAG<br>TAGGGCTACACACGTGCTACAATGGCGCATACAGAGGGCGGCCAACTTGCGAAAGTGAGCGA<br>ATCCCAAAAAGTGCGTCGTAGTCCGGATTGGAGTCTGCAACTCGACTCCATGAAGTCGGAAT<br>CGCTAGTAATCGTGGATCAGAATGCCACGGTGAATACGTTCCCGGGCCTTGTACACACCGCCC<br>GTCACACCATGGGAGTGGGCTGCAAAAGAAGTAGGTAGTTTAACCTTCGGGGGGACGCTACC<br>A
HZOS-05	*Vibrio alginolyticus*	99.85% 1367bp	AGCGGCGGACGGGTGAGTAATGCCTAGGAAATTGCCCTGATGTGGGGGATAACCATTGGAAA<br>CGATGGCTAATACCGCATGATGCCTACGGGCCAAAGAGGGGGACCTTCGGGCCTCTCGCGTC<br>AGGATATGCCTAGGTGGGATTAGCTAGTTGGTGAGGTAAKGGCTCACCAAGGCGACGATCCC<br>TAGCTGGTCTGAGAGGATGATCAGCCACACTGGAACTGAGACACGGTCCAGACTCCTACGGG<br>AGGCAGCAGTGGGGAATATTGCACAATGGGCGCAAGCCTGATGCAGCCATGCCGCGTGTGTG<br>AAGAAGGCCTTCGGGTTGTAAAGCACTTTCAGTCGTGAGGAAGGTRGTGTAGTTAATAGCTG<br>CATYAYTTGACGTTAGCGACAGAAGAAGCACCGGCTAACTCCGTGCCAGCAGCCGCGGTAAT<br>ACGGAGGGTGCGAGCGTTAATCGGAATTACTGGGCGTAAAGCGCATGCAGGTGGTTTGTTAA<br>GTCAGATGTGAAAGCCCGGGGCTCAACCTCGGAATAGCATTTGAAACTGGCAGACTAGAGTA<br>CTGTAGAGGGGGGTAGAATTTCAGGTGTAGCGGTGAAATGCGTAGAGATCTGAAGGAATACC<br>GGTGGCGAAGGCGGCCCCCTGGACAGATACTGACACTCAGATGCGAAAGCGTGGGGAGCAA<br>ACAGGATTAGATACCCTGGTAGTCCACGCCGTAAACGATGTCTACTTGGAGGTTGTGGCCTTG<br>AGCCGTGGCTTTCGGAGCTAACGCGTTAAGTAGACCGCCTGGGGAGTACGGTCGCAAGATTA<br>AAACTCAAATGAATTGACGGGGGCCCGCACAAGCGGTGGAGCATGTGGTTTAATTCGATGCA<br>ACGCGAAGAACCTTACCTACTCTTGACATCCAGAGAACTTTCCAGAGATGGATTGGTGCCTTC<br>GGGAACTCTGAGACAGGTGCTGCATGGCTGTCGTCAGCTCGTGTTGTGAAATGTTGGGTTAAG<br>TCCCGCAACGAGCGCAACCCTTATCCTTGTTTGCCAGCGAGTAATGTCGGGAACTCCAGGGA<br>GACTGCCGGTGATAAACCGGAGGAAGGTGGGGACGACGTCAAGTCATCATGGCCCTTACGAG<br>TAGGGCTACACACGTGCTACAATGGCGCATACAGAGGGCRGCCAACTTGCGARAGTGAGCGA

			ATCCCAAAAAGTGCGTCGTAGTCCGGATTGGAGTCTGCAACTCGACTCCATGAAGTCGGAAT<br>CGCTAGTAATCGTGGATCAGAATGCCACGGTGAATACGTTCCCGGGCCTTGTACACACCGCCC<br>GTCACACCATGGGAGTGGGCTGCAAAAGAAGTAGGTAGTTTAACCTTCGGGGGGACGCTACC
HZOS-06	*Vibrio alginolyticus*	99.64% 1371bp	AGCGGCGGACGGGTGAGTAATGCCTAGGAAATTGCCCTGATGTGGGGGATAACCATTGGAAA<br>CGATGGCTAATACCGCATRATGCCTACGGGCCAAAGAGGGGGACCTTCGGGCCTCTCGCGTC<br>AGGATATGCCTAGGTGGGATTAGCTAGTTGGTGAGGTAAKGGCTCACCAAGGCGACGATCCC<br>TAGCTGGTCTGAGAGGATGATCAGCCACACTGGAACTGAGACACGGTCCAGACTCCTACGGG<br>AGGCAGCAGTGGGGAATATTGCACAATGGGCGCAAGCCTGATGCAGCCATGCCGCGTGTGTG<br>AAGAAGGCCTTCGGGTTGTAAAGYACTTTCAGTCGTGAGGAAGRYRGNNNNGTTAATAGCNN<br>NNTYRTTTGACGTTAGCGACAGAAGAAGCACCGGCTAACTCCGTGCCAGCAGCCGCGGTAAT<br>ACGGAGGGTGCGAGCGTTAATCGGAATTACTGGGCGTAAAGCGCATGCAGGTGGTTTGTTAA<br>GTCAGATGTGAAAGCCCGGGGCTCAACCTCGGAATAGCATTTGAAACTGGCAGACTAGAGTA<br>CTGTAGAGGGGGGTAGAATTTCAGGTGTAGCGGTGAAATGCGTAGAGATCTGAAGGAATACC<br>GGTGGCGAAGGCGGCCCCCTGGACAGATACTGACACTCAGATGCGAAAGCGTGGGGAGCAA<br>ACAGGATTAGATACCCTGGTAGTCCACGCCGTAAACGATGTCTACTTGGAGGTTGTGGCCTTG<br>AGCCGTGGCTTTCGGAGCTAACGCGTTAAGTAGACCGCCTGGGGAGTACGGTCGCAAGATTA<br>AAACTCAAATGAATTGACGGGGGCCCGCACAAGCGGTGGAGCATGTGGTTTAATTCGATGCA<br>ACGCGAAGAACCTTACCTACTCTTGACATCCAGAGAACTTTCCAGAGATGGATTGGTGCCTTC<br>GGGAACTCTGAGACAGGTGCTGCATGGCTGTCGTCAGCTCGTGTTGTGAAATGTTGGGTTAAG<br>TCCCGCAACGAGCGCAACCCTTATCCTTGTTTGCCAGCGAGTAATGTCGGGAACTCCAGGGA<br>GACTGCCGGTGATAAACCGGAGGAAGGTGGGGACGACGTCAAGTCATCATGGCCCTTACGAG<br>TAGGGCTACACACGTGCTACAATGGCGCATACAGAGGGCRGCCAACTTGCGARAGTGAGCGA<br>ATCCCAAAAAGTGCGTCGTAGTCCGGATTGGAGTCTGCAACTCGACTCCATGAAGTCGGAAT<br>CGCTAGTAATCGTGGATCAGAATGCCACGGTGAATACGTTCCCGGGCCTTGTACACACCGCCC<br>GTCACACCATGGGAGTGGGCTGCAAAAGAAGTAGGTAGTTTAACCTTCGGGGGGACGCTACC<br>ACTT
HZOS-07	*Vibrio alginolyticus*	99.93% 1367bp	AGCGGCGGACGGGTGAGTAATGCCTAGGAAATTGCCCTGATGTGGGGGATAACCATTGGAAA<br>CGATGGCTAATACCGCATGATGCCTACGGGCCAAAGAGGGGGACCTTCGGGCCTCTCGCGTC<br>AGGATATGCCTAGGTGGGATTAGCTAGTTGGTGAGGTAAGGGCTCACCAAGGCGACGATCCC<br>TAGCTGGTCTGAGAGGATGATCAGCCACACTGGAACTGAGACACGGTCCAGACTCCTACGGG<br>AGGCAGCAGTGGGGAATATTGCACAATGGGCGCAAGCCTGATGCAGCCATGCCGCGTGTGTG<br>AAGAAGGCCTTCGGGTTGTAAAGCACTTTCAGTCGTGAGGAAGGTRGTGTAGTTAATAGCTG

				CATTATTTGACGTTAGCGACANAAGAAGCACCGGCTAACTCCGTGCCAGCAGCCGCGGTAAT<br>ACGGAGGGTGCGAGCGTTAATCGGAATTACTGGGCGTAAAGCGCATGCAGGTGGTTTGTTAA<br>GTCAGATGTGAAAGCCCGGGGCTCAACCTCGGAATAGCATTTGAAACTGGCAGACTAGAGTA<br>CTGTAGAGGGGGGTAGAATTTCAGGTGTAGCGGTGAAATGCGTAGAGATCTGAAGGAATACC<br>GGTGGCGAAGGCGGCCCCCTGGACAGATACTGACACTCAGATGCGAAAGCGTGGGGAGCAA<br>ACAGGATTAGATACCCTGGTAGTCCACGCCGTAAACGATGTCTACTTGGAGGTTGTGGCCTTG<br>AGCCGTGGCTTTCGGAGCTAACGCGTTAAGTAGACCGCCTGGGGAGTACGGTCGCAAGATTA<br>AAACTCAAATGAATTGACGGGGGCCCGCACAAGCGGTGGAGCATGTGGTTTAATTCGATGCA<br>ACGCGAAGAACCTTACCTACTCTTGACATCCAGAGAACTTTCCAGAGATGGATTGGTGCCTTC<br>GGGAACTCTGAGACAGGTGCTGCATGGCTGTCGTCAGCTCGTGTTGTGAAATGTTGGGTTAAG<br>TCCCGCAACGAGCGCAACCCTTATCCTTGTTTGCCAGCGAGTAATGTCGGGAACTCCAGGGA<br>GACTGCCGGTGATAAACCGGAGGAAGGTGGGGACGACGTCAAGTCATCATGGCCCTTACGAG<br>TAGGGCTACACACGTGCTACAATGGCGCATACAGAGGGCRGCCAACTTGCGARAGTGAGCGA<br>ATCCCAAAAAGTGCGTCGTAGTCCGGATTGGAGTCTGCAACTCGACTCCATGAAGTCGGAAT<br>CGCTAGTAATCGTGGATCAGAATGCCACGGTGAATACGTTCCCGGGCCTTGTACACACCGCCC<br>GTCACACCATGGGAGTGGGCTGCAAAAGAAGTAGGTAGTTTAACCTTCGGGGGGACGCTACC
